# Genome-Wide Identification of *WRKY* Gene Family in Pitaya Reveals the Involvement of *HmoWRKY42* in Betalain Biosynthesis

**DOI:** 10.3390/ijms231810568

**Published:** 2022-09-12

**Authors:** Canbin Chen, Fangfang Xie, Kamran Shah, Qingzhu Hua, Jiayi Chen, Zhike Zhang, Jietang Zhao, Guibing Hu, Yonghua Qin

**Affiliations:** Guangdong Provincial Key Laboratory of Postharvest Science of Fruits and Vegetables/Key Laboratory of Biology and Genetic Improvement of Horticultural Crops (South China), Ministry of Agriculture and Rural Affairs, College of Horticulture, South China Agricultural University, Guangzhou 510642, China

**Keywords:** pitaya, WRKY transcription factors, genome-wide identification, betalain biosynthesis

## Abstract

The *WRKY* gene family is a plant-specific transcription factor (TF) that regulates many physiological processes and (a) biotic stress responses. Despite this, little is known about the molecular properties and roles of *WRKY* TFs in pitaya betalain biosynthesis. Here we report the identification of 70 *WRKY* in *Hylocereus undatus,* their gene structure, locations on each chromosome, systematic phylogenetic analysis, conserved motif analysis, and synteny of *HuWRKY* genes. HmoWRKY42 is a Group IIb WRKY protein and contains a coiled-coil motif, a WRKY domain and a C_2_H_2_ zinc-finger motif (CX_5_CX_23_HXH). Results from yeast one-hybrid and transient dual-luciferase assays showed that HmoWRKY42 was a transcriptional repressor and could repress *HmocDOPA5GT1* expression by binding to its promoter. Yeast two-hybrid assays showed that HmoWRKY42 could interact with itself to form homodimers. Knocking out the coiled-coil motif of HmoWRKY42 prevented its self-interaction and prevented it from binding to the *HmocDOPA5GT1* promoter. Knocking out the WRKY domain and C_2_H_2_ zinc-finger motif sequence of HmoWRKY42 also prevented it from binding to the *HmocDOPA5GT1* promoter. The coiled-coil motif, the WRKY domain and the C_2_H_2_ zinc finger motif are key motifs for the binding of HmoWRKY42 to the *HmocDOPA5GT1* promoter. HmoWRKY42 is localized in the nucleus and possesses trans-activation ability responsible for pitaya betalain biosynthesis by repressing the transcription of *HmocDOPA5GT1*. As far as we know, no reports are available on the role of HmoWRKY42 in pitaya betalain biosynthesis. The results provide an important foundation for future analyses of the regulation and functions of the *HuWRKY* gene family.

## 1. Introduction

Pitaya is a perennial, climbing, and tropical fruit crop belonging to the genus *Hylocereus* (Cactaceae) under the order Caryophyllales, which originated in Mexico, Central America, and South America. Pitaya is considered to be a potential economic crop for harsh environmental conditions such as high temperatures, and relatively dry and poor soil [1,2]. Pitaya is also a fast-returning fruit crop, with production starting in the same year after planting and full production within 2–3 years. Pitaya fruits contain betalains, antioxidants, vitamins, soluble dietary fiber, phytalbumin, and minerals, which have positive effects on multiple health benefits and disease prevention [3,4,5,6,7,8]. Betalains are one of the major plant pigments [9] that play an important role in our lives in terms of natural food colorants [10], high nutritional value, and treating diseases with high antioxidant and anti-inflammatory capabilities [11,12,13]. Higher plants also rely on betalains for essential functions such as defense against environmental stresses including drought, ultraviolet radiation, high saline soil, and diseases [14,15,16]. Up to date, five key genes, i.e., arogenate dehydrogenase (ADH), tyrosinase (TYR), cytochrome P450 (CYP), 4,5-dihydroxy-phenylalanine (DOPA)-dioxygenase (DOD), glucosyltransferases (GTs) [13,17] and two types of TFs, i.e., MYBs [17,18,19] and WRKYs [20,21], have been characterized as key players in betalain biosynthesis. Therefore, the identification and characterization of new genes related to betalain may contribute significantly to the genetic improvement of pitaya as well as understanding the real action mechanism of betalain biosynthesis. To date, only three WRKY TFs, i.e., HmoWRKY44, HmoWRKY3, and HmoWRKY40, have been identified from pitaya transcriptome data [20,21,22]. HmoWRKY44 and HmoWRKY40 are involved in betalain accumulation by transactivating the *HmoCYP76AD1* promoter [20,21], while HmoWRKY3 is responsible for sugar accumulation through regulating the sucrose metabolic genes *HpINV2* and *HpSuSy1* [22]. Those results suggest that WRKY TFs play key roles in the secondary metabolite biosynthesis of pitaya.

WRKY family is one of the largest TF families in higher plants. WRKY proteins, which can identify and bind to W-box cis-regulatory elements, are approximately 60 amino acids with one or two highly conserved WRKYGQK motifs and no or partial/complete zinc-finger structure (C_2_H_2_ or C_2_HC) [23,24,25]. According to the number of WRKY domains and variation in zinc finger motifs, WRKY proteins are classified into four major groups [24,26,27]. Group I members contain two WRKY domains along with a C_2_H_2_ zinc-finger motif (CX_4-5_CX_22-23_HXH). Group II members have only one WRKY domain followed by a C_2_H_2_ zinc-finger motif and can be further classified into five subgroups (IIa, IIb, IIc, IId, and IIe) based on phylogenetic analysis. Group III members have one WRKY domain and a C_2_HC zinc-finger motif (CX_7_CX_23_HXC) [26]. Group IV members contain the WRKY domain but lack a complete zinc-finger structure [24].

With the increase in the available whole genome sequences, many members of the WRKY TF family have been identified in various plant species, including 81 from *Solanum lycopersicum* [27], 97 from *Actinidia* spp. [28], 116 from *Gossypium raimondii* [29], 59 from *Vitis vinifera* [24], 58 from *Prunus persica* [30], 176 from *Glycine max* [31], 147 from *Musa acuminata* and 132 from *M. Balbisiana* [32], 58 from *Beta vulgaris* [33], 97 from *Pennisetum glaucum* [25], and 94 from sorghum [34]. WRKYs have been shown to regulate various biological processes and control gene expression through a combination of positive and negative regulation, such as biotic stresses [28], salinity stresses [25,31], alkaline stresses [33], drought tolerance [35,36], cold tolerance [24,37], secondary metabolite biosynthesis [20,21], senescence [38], pollen, seed, and fruit development [30,39]. WRKY proteins function as transcriptional regulators through binding to their elements such as the W-box (C/T)TGAC(C/T), PRE4 (TGCGCTT), SURE (TAAAGATTACTAATAGGAA) or SURE-like element, and the WK box (TTTTCCAC) in the promoter regions of down-stream genes [26,40]. Although the *WRKY* genes have been widely studied in various plants, the structure and function of the *WRKY* family in pitaya remain unknown. In this study, the genome-wide identification and characterization of pitaya *WRKY* (*HuWRKYs*) genes were performed using available genomic information.

*HmoADH1*, *HmoCYP76AD1*, *HmoDODAα1,* and *HmocDOPA5GT1* are key structural genes involved in betalain biosynthesis in pitaya [17,41]. Studies have shown that the HuMYB1 TF regulates betalain biosynthesis by specifically binding to the promoters of key genes of *HmoADH1*, *HmoCYP76AD1* and *HmoDODAα1* responsible for pitaya betalain biosynthesis [17]. HmoWRKY44 and HmoWRKY40 are involved in betalain accumulation by transactivating the *HmoCYP76AD1* promoter [20,21]. However, it is still unknown whether there are other TFs that regulate the *HmocDOPA5GT1* promoter and participate in betalain biosynthesis in pitaya. In this study, a genome-wide screening was performed to identify and analyze the WRKY TFs involved in betalain biosynthesis in pitaya (HmoWRKY). The ability of HmoWRKYs to activate the promoter of *HmocDOPA5GT1* related to betalain biosynthesis was determined using yeast one-hybrid assays in yeast and transient dual-luciferase assays in *Nicotiana benthamiana* leaves. The aim of the present study was to identify WRKY TFs involved in pitaya betalain biosynthesis and candidate *HmoWRKY* genes that can be used for the genetic improvement of cultivated pitaya.

## 2. Materials and Methods

### 2.1. Plant Materials

‘Guanhuahong’ (red peel with red pulp, *H. monacanthus*), ‘Guanhuabai’ (red peel with white pulp, *H. undatus*) pitayas, ‘SCAU-YXW’ (red peel with white pulp, *H. undatus*), and *N. benthamiana* were used as materials. Pitayas were grown in the orchard of Jinsuinong (Zhongluotan Village, Guangzhou, China). Peels and pulps of ‘Guanhuahong’ and ‘Guanhuabai’ pitayas were collected on the 17th, 23rd, 25th, and 32nd days after artificial pollination (DAAP) for gene cloning and expression analyses. Scales of ‘Guanhuahong’ pitaya on the 17th DAAP and pulps of ‘SCAU-YXW’ (red peel with white pulp, *H. undatus*) pitaya on the 17th DAAP were used for virus-induced gene silencing (VIGS). Three uniformly sized fruits from every stage were sampled as three replicates. *N. benthamiana* was grown in a greenhouse with a condition of 16 h/8 h day/night at 25 °C and used for transient trans-activation assays in vivo. All samples were immediately frozen in liquid nitrogen and stored at −80 °C before use.

### 2.2. Identification and Cloning of WRKY Gene Family in Pitaya

To identify the pitaya WRKY family genes, the Arabidopsis WRKY proteins were downloaded from the Arabidopsis information resource (TAIR) (https://www.arabidopsis.org/ (accessed on 25 July 2022)), and used queries in performing two-way basic local alignment search tool (BLAST) searches with TBtools software [42] against the pitaya genome database (PRJNA691451). Candidate HuWRKY proteins were further validated via searching for WRKY conserved domains of WRKY proteins using NCBI-CDD (https://www.ncbi.nlm.nih.gov/cdd (accessed on 25 July 2022)).

Total RNA was extracted using the EASYspin Plus polysaccharide polyphenol complex plant RNA rapid extraction kit (RN53) (Aidlab, Beijing, China), and the first-strand cDNA was synthesized using the Scientific RevertAid First Strand cDNA Synthesis Kit (Thermo Fisher Scientific, Waltham, MA, USA) according to the manufacturer’s instructions. The coding sequences (CDS) of *HmoWRKY* genes were amplified from cDNA using gene-specific primers (Supplementary Text S1). Phanta Max Super-Fidelity DNA Polymerase (Vazyme, Nanjing, China) was used for PCR amplification. The purified PCR products were ligated into the pEASY-Blunt Cloning Vector (TransGen, Beijing, China) for sequencing.

### 2.3. Sequence Analysis

Multiple sequence alignments of the amino acid sequences were generated using DNAMAN software (Version 8). Each WRKY protein sequence of *H. undatus* was analyzed for its physiochemical characteristics in terms of the number of amino acids, molecular weights (MWs), and theoretical isoelectric point (pI) on the ExPASy website (http://web.expasy.org/potparam/ (accessed on 25 July 2022)).

### 2.4. Phylogenetic Analyses

The full-length amino acid sequences of WRKYs for phylogenetic trees were downloaded from *Arabidopsis thaliana* [43], *Beta vulgaris* [44], and *H. undatus* [45]. Phylogenetic tree was constructed by MEGA7 software using the neighbor-joining (NJ) algorithm with 1000 bootstraps [46]. The phylogenetic tree was annotated with EVOLVIEW (http://http://www.evolgenius.info/evolview (accessed on 25 July 2022)) [47].

### 2.5. Gene Structure Analysis and Identification of Conserved Motifs

The exon-intron structures were analyzed by TBtools software [48], and the conserved protein motif was analyzed using MEME (http://meme-suite.org/ (accessed on 25 July 2022)) website.

### 2.6. Chromosomal Distribution and Gene Synteny Analysis

The location information of *HuWRKY* genes was obtained from the pitaya genome database (PRJNA691451) and the synteny information of WRKY genes was obtained from the *A.thaliana* [43], *B. vulgaris* [44], and *H. undatus* [45]. The gene location map and the synteny analysis were constructed using TBtools software [48].

### 2.7. Measurement of Betacyanin Contents

Betacyanins were extracted according to the method of Hua, et al. [49]. The absorbance of the betacyanins was measured at wavelengths of 538 nm using a Multiskan Spectrum (Infinite M200, Tecan (Shanghai, China) Co., Ltd.). Betacyanin content was calculated as described by our previous study [49]. All determinations were performed in triplicate.

### 2.8. RT-qPCR Analysis

The expression profile for the *HmoWRKY* genes was analyzed by RT-qPCR using various tissues from VIGS and pulps of different fruit developmental stages from ‘Guanhuahong’ and ‘Guanhuabai’ pitayas. Primer sequences for RT-qPCR were designed by Primer 5 (Supplementary Text S1). The primers for *HmoCYP76AD1*, *HmoDODAα1,* and *HmocDOPA5GT1* for RT-qPCR were downloaded from our previous study [49]. RT-qPCR expression analysis was constructed following the procedure of [50]. Three biological replicates were performed for each sample.

### 2.9. Promoter Analyses

Total genomic DNA was extracted using the CTAB genomic DNA Extraction Kit (DN14) (Aidlab, Beijing, China) and RNA was removed with Ribonuclease A (RNase A) (TaKaRa Biomedical Technology Co., Ltd., Beijing, China). *HmocDOPA5GT1* was cloned from ‘Guanhuahong’ pitaya using specific primer pairs (Supplementary Text S2).

### 2.10. Yeast One-Hybrid Assay

Yeast one-hybrid assay was performed using the Matchmaker Gold Yeast One-Hybrid System (Clontech, Mountain View, CA, USA) (www.clontech.com (accessed on 25 July 2022)). The promoters of *HmocDOPA5GT1* were inserted into the reporter plasmid pAbAi (primers are listed in Table S1). The recombinant pAbAi vectors were linearized using the restriction enzyme *Bst*B I and transformed into yeast strain Y1HGold. Self-activation analyses of the promoter were performed SD/-Ura medium with a concentration gradient of 0–1000 ng/mL AbA for 3 d at 30 °C, respectively. The p53-AbAi vector was used as control.

To construct the cassette expressing the effector, the full-length CDS of *HmoWRKYs* were cloned into pGADT7 (primers are listed in Table S1). The vector was then introduced into reporter strains. The transformed reporter strains were grown on SD/-Leu media containing 300 ng/mL AbA that completely suppressed the growth of reporter strain for 3 d at 30 °C to test the possible interaction.

### 2.11. Dual-Luciferase Transient Expression Assay

The dual-luciferase transient expression assay was used to measure the effect of TFs on the transcriptional levels of downstream target genes and the transcriptional activities of TFs in tobacco leaves [51]. Transient trans-activation assay was performed as described in our previous studies [22]. The full length of *HmoWRKY30, HmoWRKY35, HmoWRKY42,* and *HmoWRKY70* was ligated into the pGreenII 62-SK vector as effectors (primers are listed in Table S1). The promoter sequence of *HmocDOPA5GT1* was ligated to the pGReenII 0800-LUC vector to construct corresponding recombinant dual luciferase reporter vectors (primers are listed in Table S1). The effector and reporter vectors were separately transformed into *Agrobacterium tumefaciens* strain GV3101, and infiltrated into *N. benthamiana* leaves in a proportion of 9:1. Three days after infiltration, leaves were determined with a Luminoskan Ascent Microplate Luminometer (Thermo) according to the method of the dual-luciferase assay kit (Promega, Madison, WI, USA).

To assess the transactivation activity of HmoWRKY42, the full-length CDS was inserted into the pGreen II BD-62-SK as an effector (primers are listed in Table S1). The reporter vector was modified from the pGreenII 0800-LUC vector [17]. The dual-luciferase transient expression system was conducted according to [17]. The trans-activation ability of HmoWRKY42 was defined by the ratio of LUC/REN using a dual-luciferase assay kit (Promega, Madison, WI, USA) after 3 d.

### 2.12. Transcriptional Activation Analyses in Yeast Cells

Full-length of *HmoWRKY42* was inserted into the pGBKT7 vector (primers are listed in Table S1). pGBKT7 and pGBKT7-53 + pGADT7-T were used as negative and positive controls, respectively. The yeast cells of strain Y2HGold separately harboring the pGBKT7-HmoWRKY42, positive and negative controls were grown on medium plates without tryptophan (SD/-Trp) or without tryptophan, histidine, and adenine (SD/-Trp-His-Ade). The transactivation activity of HmoWRKY42 protein was evaluated according to their growth status after 3 d at 30 °C and confirmed by incubating with x-α-galactosidase (X-α-Gal) for 3 h.

### 2.13. Subcellular Localization

Full-length CDS of *HmoWRKY42* was inserted into pC18-GFP (primers are listed in Table S1). Cells of the *A. tumefaciens* strain GV3101-pSoup-p19 carrying 35S-HuWRKY42-GFP and pC18-GFP (positive control) were separately infiltrated into *N. benthamiana* leaves with At1g22590-RFP (for nuclear positioning) in a ratio of 1:1. Transient expression of GFP and RFP signals were observed using a laser confocal microscope (ZEISS LCM-800, Carl Zeiss, Oberkochen, Germany) after 2 d of infiltration.

### 2.14. Gene Silence Assay

The HmoWRKY42 fragment with the conserved WRKY domain was ligated to the pTRV2 vector. pTRV1, pTRV2, and pTRV2-HmoWRKY42 were transformed into *A. tumefaciens* strain GV3101, respectively (primers are listed in Table S1). The bacterial cells were resuspended to an OD_600_ of 0.4 using MMA buffer (10 mM MES, 10 mM MgCl_2_, 100 μM acetosyringone). pTRV2 (negative control) and pTRV2-HmoWRKY42 were separately infiltrated into pitaya scales and pulps with pTRV1 in a ratio of 1:1. The experiment was carried out in the culture room with a condition of 16 h/8 h day/night at 25 °C and the results were observed after 14 d of injection.

### 2.15. Yeast Two-Hybrid (Y2H) Assay

Co-transform the constructed pGBKT7-HmoWRKY42 and mixed plasmid of pGADT7-HmoWRKYs into yeast and using Y2H experiments to find HmoWRKYs proteins that bind to HmoWRKY42. These vectors were transformed into Y2H cells according to the instructions of the Yeastmaker™ Yeast Transformation System (Clontech, Mountain View, CA, USA) (www.clontech.com (accessed on 25 July 2022)). Yeasts were grown in selective media lacking Leu and Trp (-Leu/-Trp; Clontech) and lacking Ade, His, Leu, and Trp (-Ade/-His/-Leu/-Trp; Clontech). The substrate X-α-gal was added to the media (-Ade/-His/-Leu/-Trp) for the detection of β-galactosidase activity after 3 d at 30 °C.

To search for the specific motifs that HmoWRKY42 can interact with itself to form homodimers. The fragments of *HmoWRKY42* were cloned according to the motif and ligated into pGADT7 (primers are listed in Table S1). The recombinant plasmids pGADT7-HmoWRKY42-1, pGADT7-HmoWRKY42-2, pGADT7-HmoWRKY42-3, and pGADT7-HmoWRKY42-4 were independently transformed into Y2H cells according to the experimental method described above.

### 2.16. Statistical Analysis

Data were subjected to analysis of variance and the means were compared using Student’s *t*-test at the 5% significance level using Graphpad PRISM version 9.1.1 for Mac (Graphpad Software, San Diego, CA, USA, www.graphpad.com (accessed on 25 July 2022)). The results were presented as mean standard error mean, with significance values as follows: * *p* 0.05; ** 0.01; *** *p* 0.001; **** *p* 0.0001; and non-significant (ns) (*p* > 0.05).

## 3. Results

### 3.1. Identification and Phylogenetic Analysis of WRKY Genes in Pitaya

**Figure 1:**
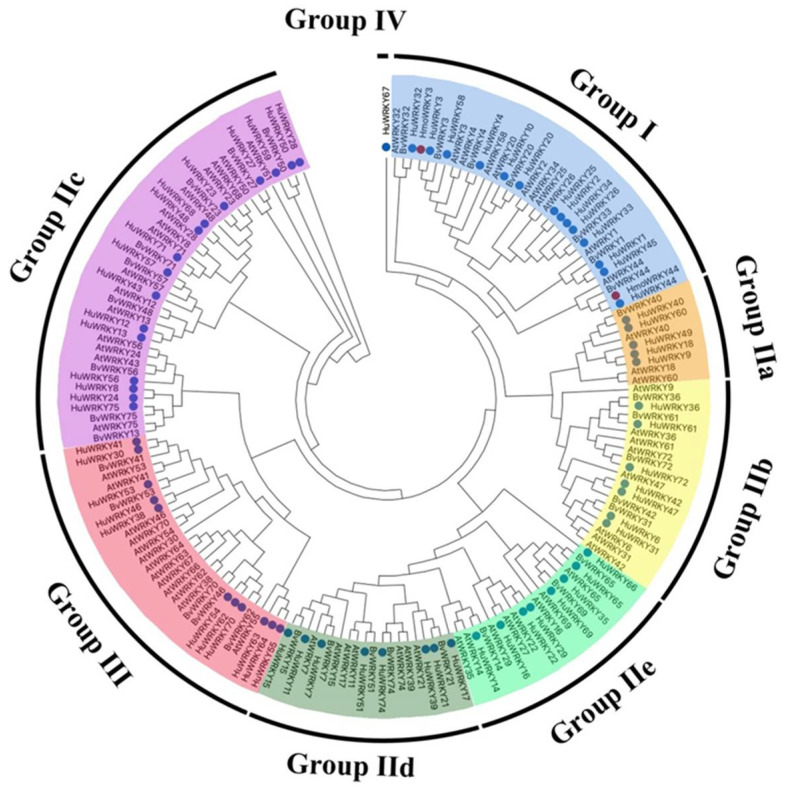
Neighbor-joining phylogenetic tree of WRKY proteins from pitaya, sugar beet, and *Arabidopsis thaliana.* WRKYs are divided into four major groups and eight sub-families. HuWRKYs are indicated by blue circles. The phylogenetic tree was constructed with MEGA7.

A total of 79 transcripts in the *H. undatus* genome sequences were identified as candidate members of the WRKY family. The 79 putative WRKY proteins were identified using a Hidden Markov Model (HMM) search program with an HMM (PF03106). After removing the redundant sequences and the incomplete sequences without the conserved WRKY domain, a total of 70 sequences were eventually identified as the pitaya *WRKY* genes and named *HuWRKY1* to *HuWRKY75* (except for *HuWRKY5*, *HuWRKY19*, *HuWRKY37*, *HuWRKY52,* and *HuWRKY73*) according to homology (Figure 1). The HuWRKY proteins with high similarity to AtWRKYs [43] or BvWRKYs [44] adopted the same name and the results are shown in Table S2.

A phylogenetic tree was constructed based on multiple sequence alignment between full-length protein sequences of 65 AtWRKYs, 35 BvWRKYs, and 70 HuWRKYs, using the NJ method in MEGAX (Figure 1). According to the classification from Arabidopsis [43], HuWRKYs were classified into three major groups: groups I, II, and III, with 14, 44, and 11 members, respectively. The remaining WRKY protein belonged to group IV following the classification of Wang, et al. [24]. A heatmap was drawn according to the FPKM values of the *HuWRKY* genes in the transcriptome database at different pulp coloration stages of ‘Guanhuahong’ and ‘Guanhuabai’ pitayas (Figure S1). The classification results were different from those in Figure 1, indicating that WRKY genes were not classified by their functions. As shown in Figure S1, eight *HuWRKYs* (*HuWRKY7*, *HuWRKY34*, *HuWRKY38*, *HuWRKY44*, *HuWRKY51*, *HuWRKY53*, *HuWRKY59*, and *HuWRKY60*) showed relatively higher expression in ‘Guanhuahong’ pitaya pulps. Among them, *HuWRKY38*, *HuWRKY53*, and *HuWRKY59* showed relatively lower expression in pulps of ‘Guanhuabai’ pitaya. However, *HuWRKY38*, *HuWRKY53*, and *HuWRKY59* were not classified into one category.

The sequence analyses of *HuWRKY* genes showed that CDS ranged from 153 bp (*HuWRKY67*) to 2367 bp (*HuWRKY25*) and predicted proteins ranged from 50 to 788 amino acids (aa) in length, with an average length of 376.3 aa. The molecular weights (MW) and isoelectric point (pI) values ranged from 5.73 kDa (*HuWRKY67*) to 85.71 kDa (*HuWRKY25*) and from 5.19 (*HuWRKY59*) to 10.12 (*HuWRKY67*), respectively (Table S2).

### 3.2. Gene Structure and Conserved Motif Analyses of HuWRKYs

**Figure 2:**
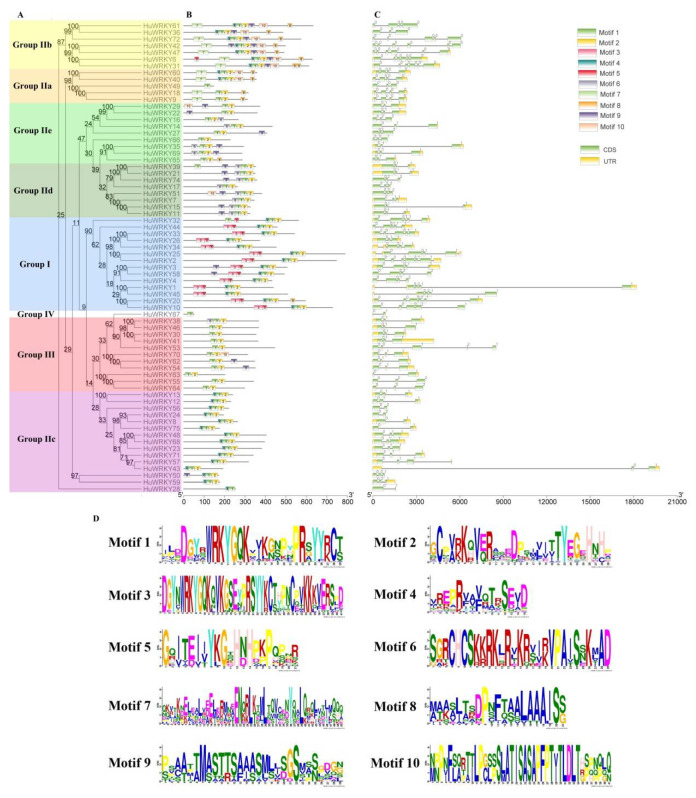
Phylogenetic relationship, gene structure, and conserved motif analysis of *HuWRKY* genes. (**A**) The NJ phylogenetic tree was constructed with MEGA7 using amino acid sequences of HuWRKYs, and the bootstrap test replicate was set to 1000 times. (**B**) The motif composition of HuWRKY proteins. Ten motifs were displayed in different colored rectangles. (**C**) Exon-intron structure of 70 *HuWRKY* genes. Green round rectangles represent exons and black lines of the same length represent introns. The yellow rectangles indicate the UTR region. Numerals such as 0, 1, and 2 represent the number of bases skipped to reach the next codon in CDS. (**D**) The amino acid sequences of 10 motifs of HuWRKY proteins.

To examine the structural characteristics of the *WRKY* genes in pitaya, the exon-intron structures and conserved motifs of HuWRKY proteins were predicted using the MEME program and further visualized by TBtools software (Figure 2B,C). A total of 10 putatively conserved motifs were identified in the HuWRKY proteins motifs, ranging from 15 to 50 aa in length (Figure 2D). NJ phylogenetic tree was individually constructed based on full-length protein sequences of 70 HuWRKY (Figure 2A), which was similar to the result in Figure 1. Group I contained 14 HuWRKY members with two conserved WRKY motifs (Motif 1 or 3). Group II had 44 members and was divided into five subgroups: group II a (5), group II b (7), group II c (15), group II d (8), and group II e (9). HuWRKY39 had two conserved WRKY motifs with similar conserved motifs with group II d. Groups III and IV each had 11 and one HuWRKY members (Figure 2A,B).

Motif 1 (WRKY motif) was widely distributed among all members of the HuWRKY family, while motif 3 (WRKY motif) was only present in group I members. Similarly, motif 7 (coiled-coil motif, CC motif) was unique to groups IIa and IIb. The motifs 8, 9, and 10 were unevenly distributed in 70 protein sequences of HuWRKY. It is interesting that motif 4 (in groups I, IIa, IIb, and IIc) and motif 6 (in groups IIe, IId, and III) do not co-exist in one HuWRKY protein. This may be related to the evolution of the *WRKY* gene [52]. The intact zinc finger motif (Motif 1 and 2) was not observed in members of HuWRKY67 (Group IV; uncharacterized). Although the proteins encoded by HuWRKY49 and HuWRKY28 did not have an intact zinc-finger motif, they belonged to groups IIa and IIc on the phylogenetic tree, respectively. Those results indicated that the sequences of HuWRKY49 and HuWRKY28 were incompletely assembled in the genome. The open reading frame (ORF) of *HmoWRKY28* was cloned from ‘Guanhuahong’ pitaya. The complete C_2_H_2_-type zinc-finger structure (CX_4-5_CX_22-23_HX_1_H) was identified in the ORF of *HmoWRKY28* (sequences were listed in Supplementary Text S1). The complete C_2_H_2_ zinc-finger structure had motifs 3 and 5, while HuWRKY6 only had a motif 5 structure. Likewise, motif 6 usually appeared before motif 1, but HuWRKY16 was different from the other proteins, suggesting that the sequences of HuWRKY6 and HuWRKY16 may have splicing errors in the genome. Some variants in the WRKYGQK domain were reported in the previous studies [53,54]. For example, the amino acids of GQK were substituted by GKK. In the present study, GQK was replaced by GKK in HuWRKY50 and HuWRKY59 (Figure 2D, Supplementary Text S1).

As shown in Figure 2C, the varying patterns of total exonic and intronic regions were detected in 70 *HuWRKYs*. The number of exons in *HuWRKYs* ranged from two to seven. A total of 37 *HuWRKY* genes (52.9%) contained the typical splicing of three exons and two introns, accounting for the largest proportion. *HuWRKY* genes in the same group had a similar number of exons, which is consistent with the other plants [33,55]. For example, genes in Group I contained 3–7 exons, covering the broadest range. For members of group II, most genes in the II c subgroup possessed two or three exons, the II d, and II e subgroups had three exons, and most genes in the II a subgroup contained four exons, while subgroup II b genes had 3–6 exons. Group III contained 11 members, and nine genes had three exons. Those results showed that the exon-intron structure was related to the phylogenetic relationship, which further supported the classification of the *HuWRKY* gene family by structure.

### 3.3. Chromosomal Localization and Synteny Analyses of HuWRKYs

**Figure 3:**
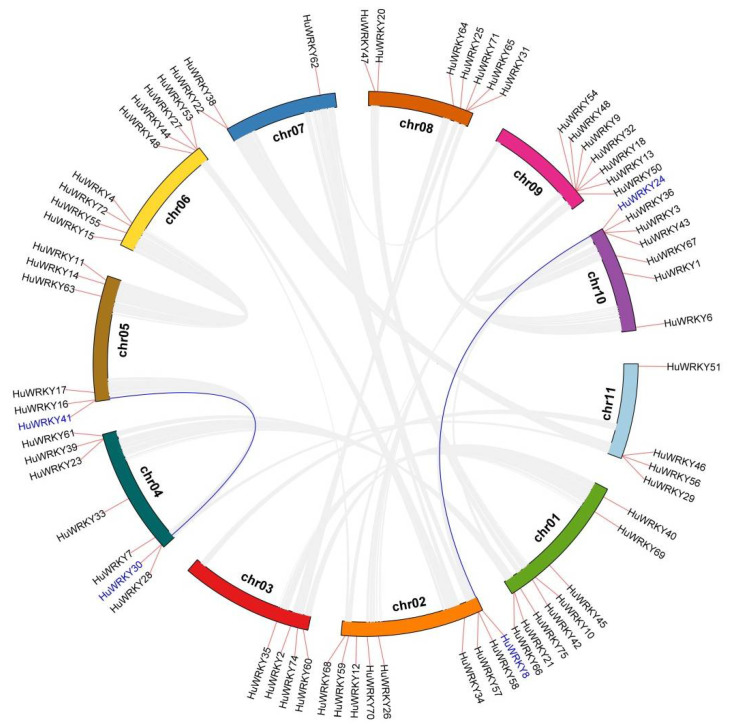
The synteny analyses of *HuWRKY* genes. Eleven chromosomes are drawn in different colors. The chromosome location of *HuWRKY* genes is shown by short red lines on the circle. Gray lines indicate all synteny blocks in the pitaya genome, and the blue lines indicate the duplication of *HuWRKY* gene pairs.

According to the gene loci information, the 70 *HuWRKY* genes were unevenly distributed on 11 chromosomes, and the detailed chromosomal locations were shown in Figure 3 and Figure S2. Most of the *HuWRKYs* were abundant on Chr 2 (9 genes; 12.9%), followed by Chr 1 (8 genes; 11.4%) and Chr 6 (8 genes; 11.4%). However, there were only three *HuWRKYs* (*HuWRKY22*, *HuWRKY38*, and *HuWRKY62*) on Chr 7. Most of the *HuWRKY* genes were located in the telomere region of chromosomes, while *HuWRKY1* and *HuWRKY33* were traced to the centromere region of chromosomes (Figure 3 and Figure S2).

To investigate the role of gene duplication in the *HuWRKY* family, segmental and tandem duplications were detected throughout the *H. undatus* genome assembly (Figure 3). There were two pairs of segmentally duplicated events. *HuWRKY8/24* and *HuWRKY30/41* may be generated by fragment duplication. A total of 26 *HuWRKY* genes were clustered into 11 tandem duplication events, indicating that tandem duplication events acted as a major force in driving the evolution of the *HuWRKY* gene family.

**Figure 4:**
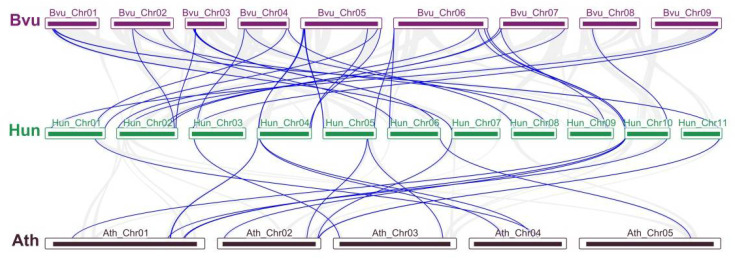
The synteny analyses of WRKY genes between *H. undatus*, *A. thaliana*, and *B. vulgaris*. Gray lines indicate all synteny blocks in the genome, and the blue lines indicate the duplication of gene pairs.

To identify the duplication events, synteny relationships were analyzed among the WRKYs of *H. undatus*, *A. thaliana,* and *B. vulgaris*. A total of 25 chromosomes (11 from *H. undatus*, 5 from *A. thaliana*, and 9 from *B. vulgaris*) were used to map the synteny relationships (Figure 4). In the genome, the synteny blocks of pitaya and sugar beet were more than those of pitaya and *A. thaliana* (gray lines), while the duplication of *WRKY* gene pairs was similar (blue lines). In total, 33 pairs of segmentally duplicated events appeared unevenly in 20 chromosomes of pitaya and sugar beet (Supplementary Table S3). Sixteen pairs of segmental duplicated events were found between pitaya and *A. thaliana*, and the segmental duplicated events of *WRKY* genes did not occur on chromosomes 2, 8, and 9 in pitaya (Supplementary Table S4). Those results suggested that pitaya was more closely related to sugar beet than Arabidopsis.

### 3.4. HmoWRKY30, HmoWRKY35, HmoWRKY42, and HmoWRKY70 could Bind the Promoter of HmocDOPA5GT1

WRKY TFs are involved in betalain biosynthesis [20,21]. Our previous studies showed that *HmocDOPA5GT1* plays a key role in the betalain biosynthesis of pitaya [41,49]. The promoter of *HmocDOPA5GT1* was cloned from ‘Guanhuahong’ pitaya. The typical W-box core sequences (C/T)TGAC(C/T) were identified in the promoters of *HmocDOPA5GT1* (sequences were listed in Supplementary Text S2). The W-box is a cognate binding site for WRKY TFs, suggesting the possible involvement of WRKY TFs in regulating *HmocDOPA5GT1*.

**Figure 5:**
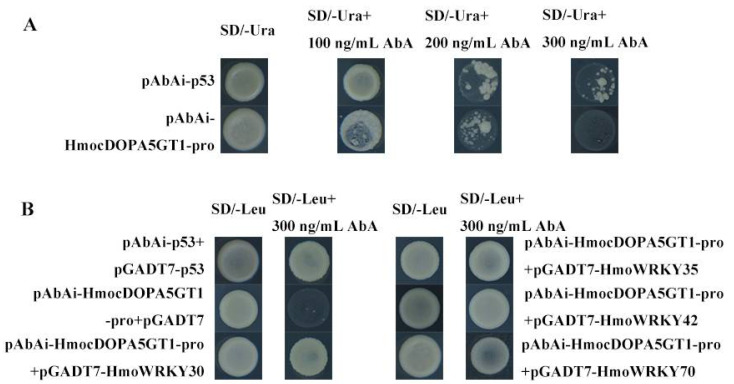
Yeast one-hybrid assays between 53 HmoWRKYs and the *HmocDOPA5GT1* promoter. (**A**) Self-activation of the *HmocDOPA5GT1* promoter. (**B**) Yeast one-hybrid assays between HmoWRKY30/35/42/70 and the *HmocDOPA5GT1* promoter.

The ORFs of *HmoWRKYs* were cloned from pulps of ‘Guanhuahong’ pitaya (Supplementary Text S1). Among them, 17 *HmoWRKYs* (*HmoWRKY2*, *HmoWRKY4*, *HmoWRKY6*, *HmoWRKY10*, *HmoWRKY22*, *HmoWRKY28*, *HmoWRKY29*, *HmoWRKY36*, *HmoWRKY47*, *HmoWRKY53*, *HmoWRKY55*, *HmoWRKY61*, *HmoWRKY62*, *HmoWRKY63*, *HmoWRKY64*, *HmoWRKY65*, *HmoWRKY72*) had a low abundance in the pulps of pitayas and have not been cloned. The interaction between 53 HmoWRKYs and the promoter of *HmocDOPA5GT1* was analyzed using a yeast one-hybrid assay. Yeast cells harboring pABAi-HmocDOPA5GT1-pro could not grow on an SD/-Ura medium supplemented with 300 ng/mL AbA (Figure 5A). The p53-AbAi control has a minimal inhibitory concentration of 100 ng/mL AbA. Yeast cells containing pABAi-HmocDOPA5GT1-pro+pGADT7-HmoWRKY30/pGADT7-HmoWRKY35/pGADT7-HmoWRKY42/pGADT7-HmoWRKY70 grew normally on SD/-Leu medium with the addition of 300 ng/mL AbA (Figure 5B). These results indicated that the HmoWRKY30/35/42/70 proteins could bind to the promoter of *HmocDOPA5GT1,* suggesting that HmoWRKY30/35/42/70 proteins were likely involved in the betalain biosynthesis of pitaya.

### 3.5. HmoWRKY42 Is a Nucleus Localized Transcription Repressor

**Figure 6:**
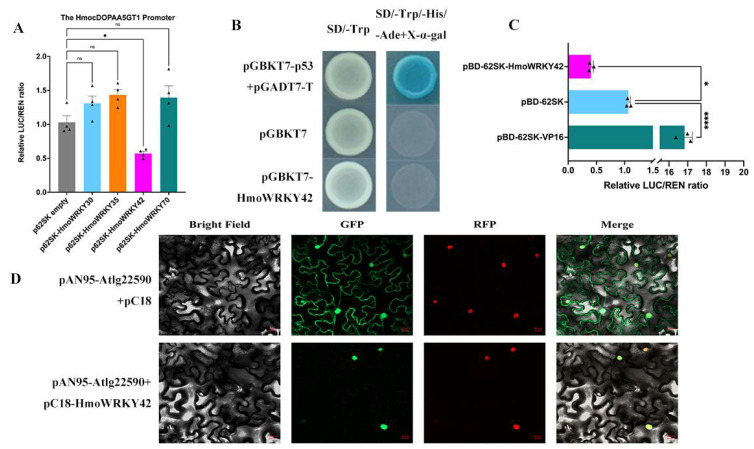
HmoWRKY42 is a nucleus-localized transcription repressor. (**A**) HmoWRKY42 inhibited the transcription of *HmoCDOPA5GT1* by dual-luciferase transient expression assay in *Nicotiana benthamiana* leaves. (**B**) Analysis of the transcriptional activity of HmoWRKY42 in yeast cells. (**C**) Transcriptional activation of HmoWRKY42 in *N. benthamiana* leaves. The LUC/REN ratio of the empty BD-62SK vector was used as a calibrator (set as 1). BD-62SK-VP16 was used as a positive control. (**D**) Subcellular localization of HmoWRKY42 in the leaves of *N. benthamiana*. Bars = 20 μm. Small triangles represent the distribution of data for each biological replicate. Data represent mean values from three biological replicates (±S.D.). Non- significant (ns), *, and **** indicates significant differences at *p*-value > 0.05, <0.05, and 0.0001 using two-tailed *t*-test, respectively.

The abilities of HmoWRKY30/35/42/70 to activate/repress the transcription of the *HmocDOPA5GT1* promoter were performed in *N. benthamiana* leaves (Figure 6A). No significant difference in the LUC/REN ratios was detected when HmoWRKY30/35/70 were co-expressed with the *HmocDOPA5GT1* promoter. Compared to the empty control, co-expression of HmoWRKY42 with *HmocDOPA5GT1* promoters significantly decreased LUC/REN ratios. HmoWRKY42 could repress the transcription of the *HmocDOPA5GT1* promoter according to transient dual-luciferase assays. These results suggest that HmoWRKY42 plays a functional role in the betalain biosynthesis of pitaya.

CDS of HmoWRKY42 was cloned into the pGBKT7 vector to study its transcriptional activation abilities. As shown in Figure 6B, the transformed yeast cells of positive control (pGBKT7-p53 + pGADT7-T) grew well in SD/-Trp-His-Ade and showed x-α-galactosidase (X-α-Gal) activity. While yeast cells containing pGBKT7 (negative control) and pGBKT7-HmoWRKY42 did not, suggesting that HmoWRKY42 had no transactivation activities in yeast cells and probably functions as transcriptional repressors in the regulation of gene expression. The transcriptional activation ability of HmoWRKY42 was further confirmed in *N. benthamiana* leaves using the dual-luciferase reporter system. Compared to the ratio of positive control (pBD-62SK-VP16) and negative control (BD-62SK), co-transformation of the pBD-62SK-HuWRKY42 with the reporter apparently decreased luciferase (LUC)/renilla luciferase (REN) ratios (Figure 6C). These results demonstrated that HmoWRKY42 was a transcriptional repressor.

To investigate the subcellular localization of *HmoWRKY42*, the full-length CDS was fused into the pC18-GFP vector. As shown in Figure 6D, *HmoWRKY42* was detected exclusively in the nucleus, while the fluorescence of the GFP-positive control was observed in both the nucleus and cytoplasm.

### 3.6. Betalain Contents and Expression Profiles of HmoWRKY42 during the Pulp Coloration of Pitayas

**Figure 7:**
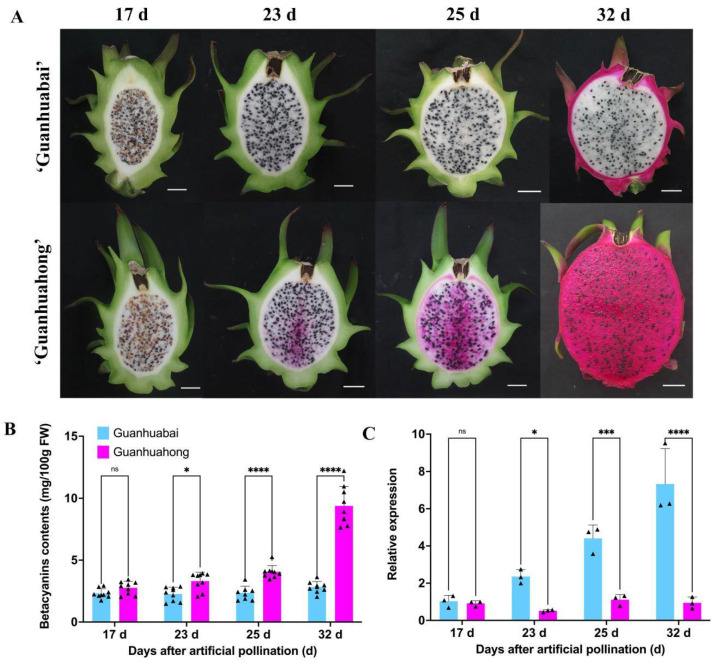
Betalain content and expression profiles of *HmoWRKY42* during different pulp coloration stages of ‘Guanhuahong’ and ‘Guanhuabai’ pitayas. (**A**) Fruit developmental stages of ‘Guanhuahong’ and ‘Guanhuabai’ pitayas. Bars = 2 cm. (**B**) The contents of betacyanin during fruit developmental stages in ‘Guanhuahong’ and ‘Guanhuabai’ pitayas. (**C**) The expression analysis of *HmoWRKY42*. Small triangles represent the distribution of data for each biological replicate. Data represent mean values from three biological replicates (±S.D.). Non- significant (ns), *, ***, and **** indicates significant differences at *p*-value > 0.05, <0.05, 0.001, and 0.0001 using two-tailed *t*-test, respectively.

The ORF of *HmoWRKY42* was cloned from pulps of ‘Guanhuahong’ and ‘Guanhuabai’ pitayas (Supplementary Text S1), respectively. *HmoWRKY42* shared 99.2% of its identity between the two cultivars (Supplementary Text S1). As shown in Figure 7A, the pulps of ‘Guanhuahong’ and ‘Guanhuabai’ pitayas had different coloring times. The pulp of the ‘Guanhuahong’ pitaya began to turn red on the 23rd DAF and gradually deepened during the fruit development stage, while the pulp of the ‘Guanhuabai’ pitaya remained white. In the pulps of ‘Guanhuahong, the betacyanin content increased significantly during fruit coloration, compared to being relatively stable in the pulps of ‘Guanhuabai’ pitaya (Figure 7B). The contents of betacyanins in pulps of ‘Guanhuahong’ were significantly higher than those of ‘Guanhuabai’ pitaya during pulp coloration. HmoWRKY42 showed significantly up-regulated expression in pulps of ‘Guanhuabai’ pitaya during fruit development. However, no significant difference was detected in the pulps of the ‘Guanhuahong’ pitaya during fruit development (Figure 7C). Those results indicated that the expression difference of HmoWRKY42 may be responsible for the different pulp colors between ‘Guanhuahong’ and ‘Guanhuabai’ pitayas.

### 3.7. Identification of HmoWRKY42 Involved in Pitaya Betalain Biosynthesis

**Figure 8:**
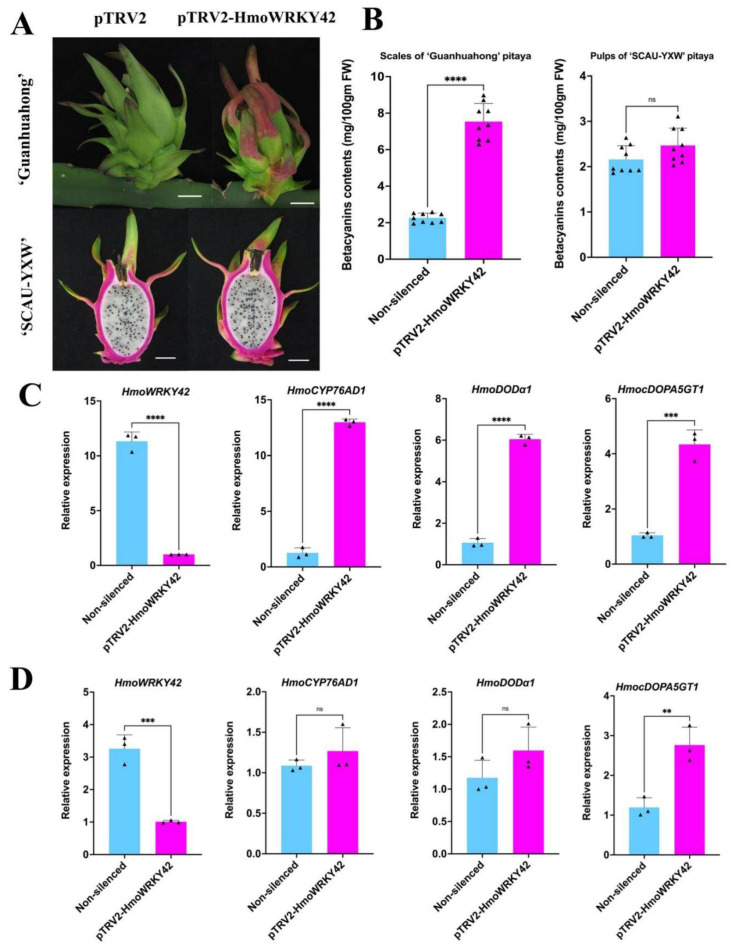
Silence of *HmoWRKY42* promotes betalain production. (**A**) Virus-induced gene silencing of *HmoWRKY42* in green scales and white pulps. Bars = 3 cm. (**B**) Betacyanin contents in pitaya scales and pulps after virus-induced silencing of *HmoWRKY42*. (**C**) RT-qPCR analyses after virus-induced silencing of *HmoWRKY42* in scales of ‘Guanhuahong’ pitaya. (**D**) RT-qPCR analyses after virus-induced silencing of *HmoWRKY42* in pulps of ‘SCAU-YXW’ pitaya. Small triangles represent the distribution of data for each biological replicate. Data represent mean values from three biological replicates (±S.D.). Non- significant (ns), **, ***, and **** indicates significant differences at *p*-value > 0.05, <0.01, 0.001, and 0.0001 using two-tailed *t*-test, respectively.

Gene silencing assay was performed to further elucidate the function of *HmoWRKY42*. Compared to control, silencing of *HmoWRKY42* exhibited earlier red pigmentation in scales of ‘Guanhuahong’ pitaya (*H. monacanthus*) and resulted in an increase in betacyanin accumulation (Figure 8A,B). Results from RT-qPCR analyses confirmed that *HmoWRKY42* was silenced while betalain biosynthesis-related genes such as *HmoCYP76AD1*, *HmoDODAα1,* and *HmocDOPA5GT1* were significantly up-regulated in scales of ‘Guanhuahong’ pitaya (Figure 8C). However, no pigmentation was observed when *HmoWRKY42* alone was silenced in pulps of ‘SCAU-YXW’ pitaya (*H. undatus*) (Figure 8A,B). Expression levels of *HmocDOPA5GT1* in pulps of ‘SCAU-YXW’ pitaya were significantly higher than that of control (Figure 8D) compared to no significant difference was detected in the expression of *HmoCYP76AD1* and *HmoDODAα1*. These results indicated that *HmoWRKY42* plays an important role in pitaya betalain biosynthesis. Silencing of *HmoWRKY42* could promote the expression of *HmocDOPA5GT1,* resulting in earlier red pigmentation in the scales of the ‘Guanhuahong’ pitaya.

### 3.8. Determination of Binding Motif

**Figure 9:**
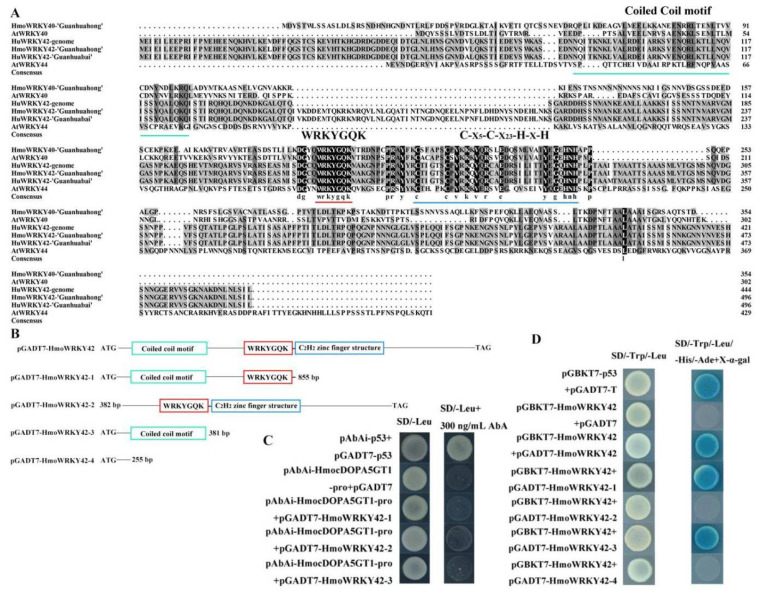
HmoWRKY42 could bind a coiled coil motif and interact with itself to form homodimers. (**A**) The alignment analyses of HmoWRKY42. Coiled coil motif, WRKY domains, and zinc-finger structures are labeled in green, red, and blue underlines, respectively. (**B**) Diagrams of the pGADT7-HmoWRKY42-1, pGADT7-HmoWRKY42-2, pGADT7-HmoWRKY42-3, and pGADT7-HmoWRKY42-4 sequences. (**C**) Yeast one-hybrid assays between the *HmocDOPA5GT1* promoter and the motif of HmoWRKY42. (**D**) Yeast two-hybrid assays between HmoWRKY42 and itself.

HmoWRKY42 contained a 1491 bp ORF and encoded a protein of 496 amino acid residues with a predicted mass of 54.35 kDa and a calculated pI = 6.51 (Table S2). HmoWRKY42 protein contained a WRKY domain, a zinc-finger structure of C_2_H_2,_ and a coiled-coil motif. As well, the coiled-coil motif of HmoWRKY42 protein contained 42 amino acid residues (Figure 9A). Yeast one/two-hybrid assays were performed to study which motif on the HmoWRKY42 sequence is responsible for the binding of HmoWRKY42 to the *HmocDOPA5GT1* promoter and the interaction between HmoWRKY42 and itself (Figure 9B). The HmoWRKY42 region from 1 to 381 bp, 1 to 855 bp, and 382 to 1491 bp were used in the yeast one-hybrid assays to determine which motif on the HmoWRKY42 sequence was responsible for the binding of HmoWRKY42 to *HmocDOPA5GT1* promoter. When the WRKY domain, zinc-finger, or coiled-coil sequence of HmoWRKY42 was knocked out, HmoWRKY42 could not bind the promoter of *HmocDOPA5GT1* (Figure 9C), indicating that the WRKY domain, zinc-finger, and coiled-coil motif were required for binding between HmoWRKY42 and the promoter of *HmocDOPA5GT1*. Yeast strains harboring pGADT7-HmoWRKY42 and pGBKT7-HmoWRKY42 grew normally on –Leu/–Trp and –Ade/–His/–Leu/–Trp selective media, and the yeast strains turned blue on the –Ade/–His/–Leu/–Trp selective medium supplemented with the substrate X-α-gal. On the contrary, the yeast strains cotransformed with pGADT7 and pGBKT7-HmoWRKY42 (negative control) could grow on the –Leu/–Trp medium but not on the –Ade/–His/–Leu/–Trp selective medium. Those results indicated that HmoWRKY42 could interact with itself to form homodimers. When the coiled-coil motif of HmoWRKY42 was knocked out, HmoWRKY42 could not self-interact (Figure 9D).

## 4. Discussion

WRKY proteins play critical roles in plant physiological processes, diverse biotic/abiotic stress responses, and secondary metabolite synthesis [20,25,33,37,56,57,58,59]. Our previous studies indicated that WRKYs are involved in pitaya betalain biosynthesis [20,21]. However, the genome-wide analysis of the *WRKY* gene family has not been studied in pitaya. In this study, we identified and characterized HuWRKYs through genome-wide analyses using bioinformatics.

A total of 70 HuWRKY proteins were identified and classified into four major groups (I–IV) based on the presence of WRKY DNA binding domains and zinc-finger motif structures (Figure 1 and Figure 2). Those results showed that there is a strong correlation between motif structure and phylogenetic relationships, which additionally supports the classification of the *HuWRKY* gene family [59]. Members of group II have been further subdivided into five subgroups (IIa-IIe) based on the phylogenetic analysis (Figure 2), which was consistent with the other plants [24,25]. The phenomenon that motif 4 (in groups I, IIa, IIb, and IIc) and motif 6 (in groups IIe, IId, and III) cannot co-exist in one WRKY protein also appears in sorghum [34], indicating that the close phylogenetic relationship was shown by subgroups I, IIa, IIb and IIc, likewise IIe, IId and III. All *HuWRKY* genes share the highly conserved WRKYGQK motif (Figure 2). Members of group IV (HuWRKY67) had no zinc finger structure (C_2_H_2_ or C_2_HC) within the DNA binding domain following the WRKYGQK sequence, and it is still unclear whether they affect the function and expression of WRKY genes [25]. A variant of the WRKYGQK motif was found in HuWRKY50 and HuWRKY59 (WRKYGKK), suggesting that HuWRKY50 and HuWRKY59 may alter their DNA binding affinity [59].

Gene duplication events play a vital role in the expansion and evolutionary progress of gene families and the creation of novel biological functions [55]. In this study, two segmental duplicated events (*HuWRKY8/24* and *HuWRKY30/41*) and 11 tandem duplication events were identified in *HuWRKYs* (Figure 3). Compared with *V. vinifera* [24], soybean [31], and *Oryza rufipogon* [55], tandem duplication events mainly contributed to the expansion of *HuWRKY* genes. Comparative mapping in the synteny relationship of *H. undatus* between *A. thaliana* and *B. vulgaris* was established based on the genome (Figure 4). The *WRKY* segmental duplicated events of *H. undatus* and *B. vulgaris* (33 pairs) were 2-fold higher than those of *H. undatus* and *A. thaliana* (16 pairs). Those results demonstrated that pitaya is more closely related to sugar beet (accumulating betalains) than to *A. thaliana* (accumulating anthocyanins).

**Figure 10:**
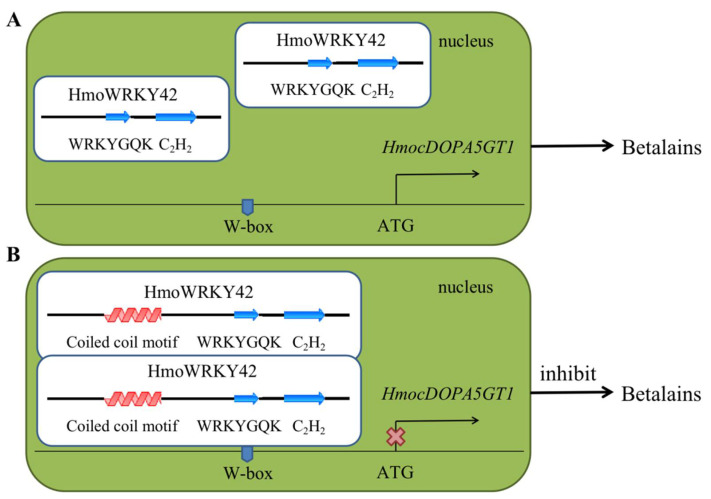
The regulatory model of HmoWRKY42 involved in betalain biosynthesis of pitayas. (**A**) Knocking out the coiled-coil motif of HmoWRKY42 prevented its self-interaction and prevented it from binding to the *HmocDOPA5GT1* promoter. (**B**) The coiled-coil motif, the WRKY domain and the C_2_H_2_ zinc finger motif are key motifs for the binding of HmoWRKY42 to the *HmocDOPA5GT1* promoter.

The peel and pulp of pitaya exhibited different colors, mainly depending on the synthesis and accumulation of betalains [8]. Color is an important parameter of pitaya quality, and it is also an important indicator to determine the commercial value of the fruit. In our previous study, *HmoADH1*, *HmoCYP76AD1*, *HmoDODAα1,* and *HmocDOPA5GT1* played key roles in the betalain biosynthesis of pitaya [17,47,55]. HmoWRKY44 and HmoWRKY40 proteins were classified into differential groups with the same function in betalain biosynthesis. HmoWRKY44 and HmoWRKY40 TFs belong to members of the group I and IIa WRKY family, respectively, and both of them could activate *HmoCYP76AD1* expression by binding to its promoter responsible for betalain biosynthesis of pitaya [20,21]. In this study, the ORF of *HmoWRKY40*, *HmoWRKY42,* and *HmoWRKY44* were cloned from pulps of ‘Guanhuahong’ (*H. monacanthus*) and ‘Guanhuabai’ (*H. undatus*) pitayas, respectively. No difference in cDNA sequences of *HmoWRKY40* and *HmoWRKY44* was detected between ‘Guanhuahong’ and ‘Guanhuabai’ pitayas (Supplementary Text S1). *HmoWRKY42* shared 99.2% of its identity between the two cultivars (Supplementary Text S1). HmoWRKY42 was localized exclusively in the cell nucleus (Figure 6D), which was consistent with *HmoWRKY40* and *HmoWRKY44* [20,21]. HmoWRKY40 and HmoWRKY44 could activate the transcription of *HmoCYP76AD1* involved in pitaya betalain biosynthesis [20,21]. HmoWRKY42 was a repressor and bonded to the *HmocDOPA5GT1* promoter (Figure 6 and Figure 8). The regulatory model of HmoWRKY42 involved in betalain biosynthesis of pitayas is shown in Figure 10. The expression levels of *HmoWRKY40* and *HmoWRKY44* significantly increased during pulp coloration of *H. monacanthus* [20,21], which was inconsistent with *HmoWRKY42*. In our study, *HmoWRKY42* showed relatively higher expression during the fruit development of *H. undatus* (Figure 7C). Knocking out the WRKY domain, coiled-coil motif, or C_2_H_2_ zinc-finger structure of HmoWRKY42 prevented it from binding to the promoter of *HmocDOPA5GT1*. Therefore, HmoWRKY42 needs to bind to the coiled-coil motif, the WRKY domain, and the C_2_H_2_ zinc finger structure to regulate the expression of *HmocDOPA5GT1* (Figure 9). In addition, HmoWRKY42 could not self-interact when the coiled-coil motif of HmoWRKY42 was knocked out (Figure 9), which indicated that the coiled-coil motif was required for HmoWRKY42 homodimer formation.

## 5. Conclusions

In summary, our study provides the first genome-wide analysis of the *WRKY* family in pitaya. A total of 70 HuWRKY proteins were obtained and can be classified into eight subgroups. The 70 HuWRKY genes were unevenly distributed among all the 11 chromosomes of pitaya. A novel *WRKY* gene, i.e., *HmoWRKY42*, belonging to a member of Group IIb, was obtained. The expression pattern of *HmoWRKY42* was correlated well with betalain accumulation during fruit maturation of ‘Guanhuabai’ pitaya. However, *HmoWRKY42* was kept at a stable low expression level during fruit maturation in ‘Guanhuahong’ pitaya. HmoWRKY42 was a nuclear-localized transcriptional repressor and could repress *HmocDOPA5GT1* expression by binding to its promoter. HmoWRKY42 was capable of forming homodimers and could bind the coiled-coil motif, the WRKY domain, and the C_2_H_2_ zinc finger structure in the *HmocDOPA5GT1* promoter. The present study provides novel insights into the functional divergence of WRKY TFs involved in the betalain biosynthesis of pitaya.

## Data Availability

Data is contained within the article and Supplementary Materials.

## Supplementary Materials

The following supporting information can be downloaded at: https://www.mdpi.com/article/10.3390/ijms231810568/s1.

## Abbreviations

aa: amino acids; AbA: aureobasidin A; ADH: arogenate dehydrogenase; BLAST: basic local alignment search tool; bp: base pair; CC: coiled coil; CDD: conserved domains database; cDNA: complementary DNA; cDOPA: cyclo-4,5-dihydroxy-phenylalanine; CDS: coding sequence; CTAB: Cetytrimethylammonium bromide; CYP: cytochrome P450; DAAP: day after artificial pollination; DOD: dioxygenase; DOPA: 4,5-dihydroxy-phenylalanine; GFP: Green fluorescent protein; GTs: glucosyltransferases; HMM: Hidden Markov Model; kDa: kilodaltons; LUC: luciferase; Mb: million base pair; MES: (2-N-morpholino) ethanesulfonic acid; MW: molecular weight; MYB: myb proto-oncogene protein; NCBI: National Center for Biotechnology Information; NJ: neighbor-joining; ORF: open reading frame; pI: theoretical isoelectric point; REN: renilla; RFP: Red fluorescent protein; RT-qPCR: reverse transcription quantitative real-time polymerase chain reaction; TAIR: The Arabidopsis Information Resource; TFs: transcription factors; TYR: tyrosinase; VIGS: virus-induced gene silencing; WRKY: wrky protein.
