# Supplementary material for: Genome-Wide Identification of WRKY Gene Family in Pitaya Reveals the Involvement of HmoWRKY42 in Betalain Biosynthesis
Source: Int J Mol Sci. 2022 Sep 12;23(18):10568. doi: 10.3390/ijms231810568 (PMC9502481; doi:10.3390/ijms231810568)
Supplement: Supplementary file 1 [file ijms-23-10568-s001.zip › Figure S1. The expression heatmap of WRKY genes according to the RNA-Seq datasets.pdf]

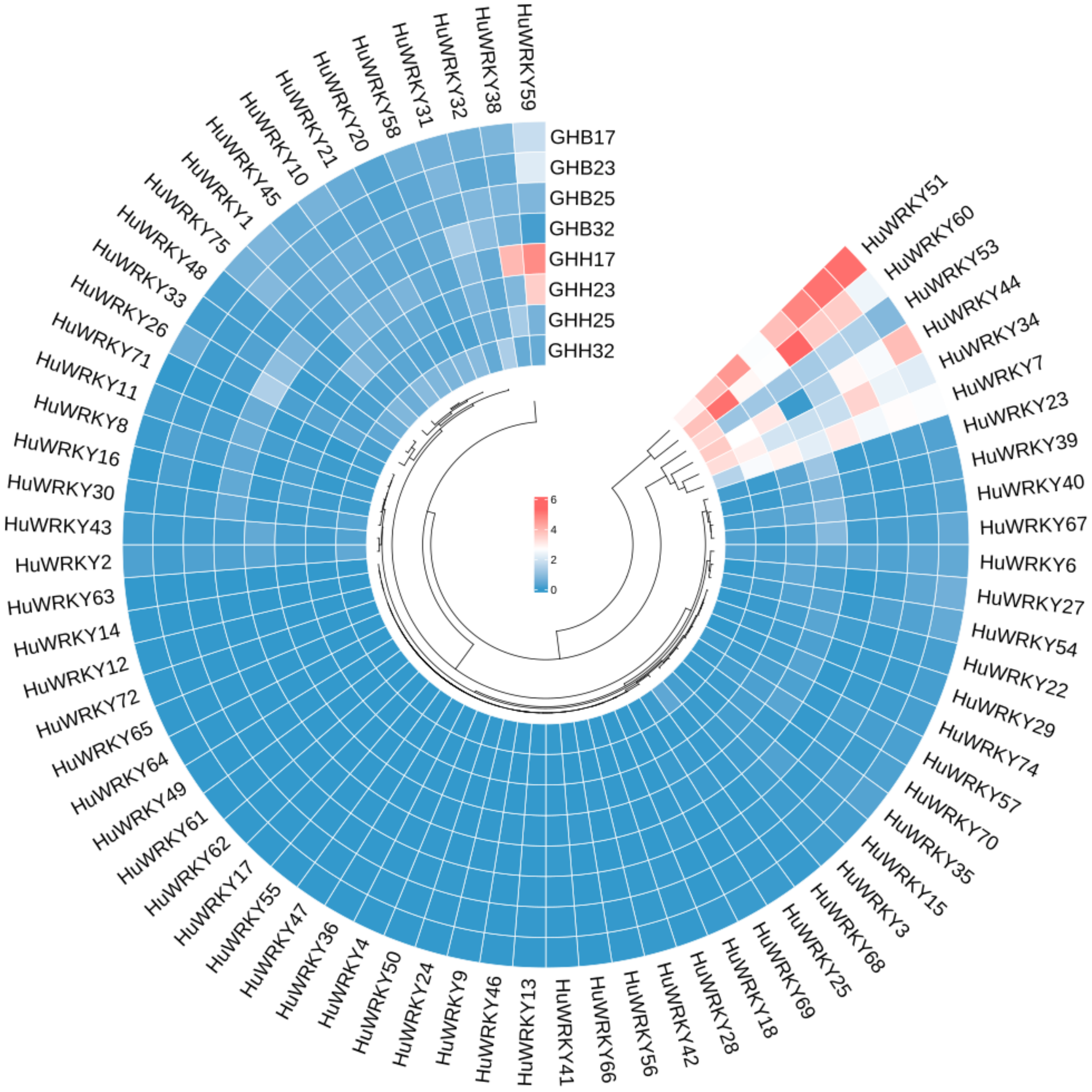

Figure S1. The expression heatmap of *WRKY* genes according to the RNA-Seq datasets of 'Guanhuahong' (GHH) and 'Guanhuabai' (GHB) pitaya pulps. The color bar represents the $\log_2$(FPKM) and ranges from blue (low expression) to red (high expression).
