# Supplementary material for: Genome-Wide Identification of WRKY Gene Family in Pitaya Reveals the Involvement of HmoWRKY42 in Betalain Biosynthesis
Source: Int J Mol Sci. 2022 Sep 12;23(18):10568. doi: 10.3390/ijms231810568 (PMC9502481; doi:10.3390/ijms231810568)
Supplement: Supplementary file 1 [file ijms-23-10568-s001.zip › Figure S2. Schematic representations of the chromosomal location of the HuWRKY genes.pdf]

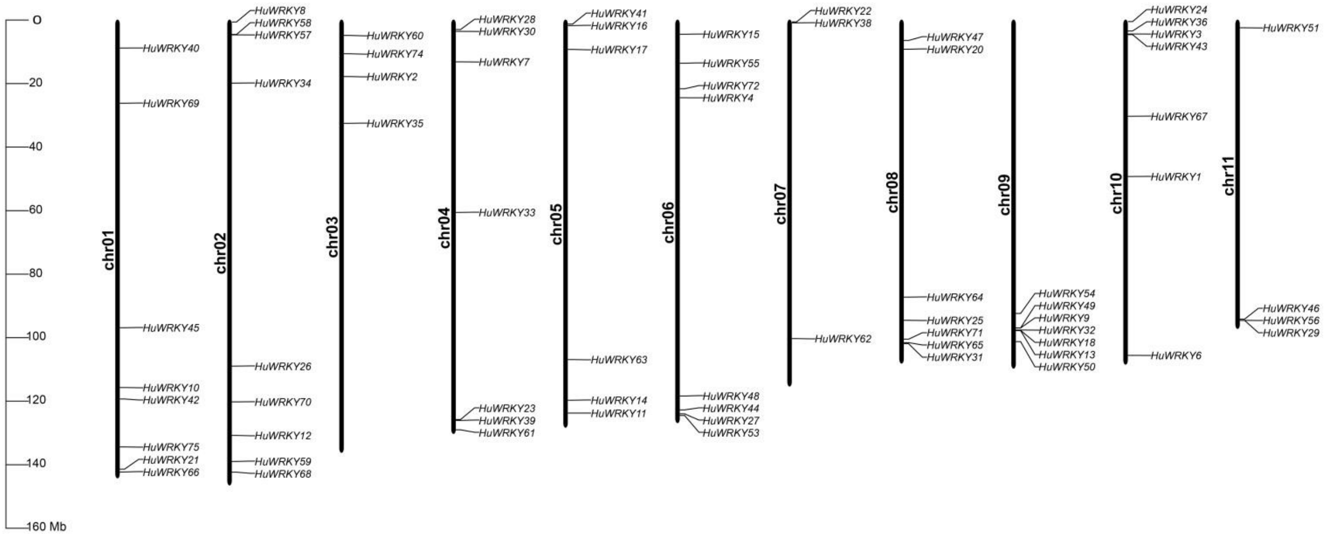

Figure S2. Schematic representations of the chromosomal location of the *HuWRKY* genes. Eleven chromosomes with varying lengths are shown in Mb (million base pair) scale in the left, the chromosome number is shown in the middle of each chromosome.
