# Supplementary material for: Genome-Wide Identification of WRKY Gene Family in Pitaya Reveals the Involvement of HmoWRKY42 in Betalain Biosynthesis
Source: Int J Mol Sci. 2022 Sep 12;23(18):10568. doi: 10.3390/ijms231810568 (PMC9502481; doi:10.3390/ijms231810568)
Supplement: Supplementary file 1 [file ijms-23-10568-s001.zip › Supplementary Table S1. Summary of primers used in pitaya.pdf]

Supplementary Table S1. Summary of vector primers used in this study.

Assay	Primer sequences	Restriction Site
	pGADT7-HmoWRKY1-F	
	GGCCATGGAGGCCAGTGAATTCATGGTTGCAAAGGAGGAAGC	
	pGADT7-HmoWRKY1-R	
	GCAGCTCGAGCTCGATGGATCCTCAGCAATTAACAGGCTCAGC	
	pGADT7-HmoWRKY3-F	
	GGCCATGGAGGCCAGTGAATTCATGCCGGAAAACGATGCCG	
	pGADT7-HmoWRKY3-R	
	GCAGCTCGAGCTCGATGGATCCCTATGTTATTCTCTCTTGTTTTAA	
	pGADT7-HmoWRKY7-F	
	GGCCATGGAGGCCAGTGAATTCATGGCAGTGGAGCTTATGGC	
	pGADT7-HmoWRKY7-R	
	GCAGCTCGAGCTCGATGGATCCCTAAGATGATTCGAGGACCATGG	
	pGADT7-HmoWRKY8-F	
	GGCCATGGAGGCCAGTGAATTCATGCATCTTCTTCTTCTTTATTATG	
	pGADT7-HmoWRKY8-R	
	GCAGCTCGAGCTCGATGGATCCTTAAAGCGAAGTGTAGATTTGC	
	pGADT7-HmoWRKY9-F	
	GGCCATGGAGGCCAGTGAATTCATGGACTCATCTAAGTGGATAC	
	pGADT7-HmoWRKY9-R	
Yeast	GCAGCTCGAGCTCGATGGATCCTTAAAGCCATCTCTTAGAATGACT	*Eco*RI
one/two-hybrid	pGADT7-HmoWRKY11-F	
assay	GGCCATGGAGGCCAGTGAATTCATGGCTATAGATCTCATGGTAGG	*Bam*HI
	pGADT7-HmoWRKY11-R	
	GCAGCTCGAGCTCGATGGATCCTTAAGATGACTCGAGAATAGACG	
	pGADT7-HmoWRKY12-F	
	GGCCATGGAGGCCAGTGAATTCATGTTGAACCAGGAGAACTTG	
	pGADT7-HmoWRKY12-R	
	GCAGCTCGAGCTCGATGGATCCCTACCAAAAGAAGTTATTGAGAA	
	pGADT7-HmoWRKY13-F	
	GGCCATGGAGGCCAGTGAATTCATGTTGAACAGCCAAGGTTTACTC	
	pGADT7-HmoWRKY13-R	
	GCAGCTCGAGCTCGATGGATCCCTAACAGAAGAAGTTACTAAGAA	
	pGADT7-HmoWRKY14-F	
	GGCCATGGAGGCCAGTGAATTCATGGACAATTATCAAGGGGATCT	
	pGADT7-HmoWRKY14-R	
	GCAGCTCGAGCTCGATGGATCCTCAAGAAACCCTTTTTTCTTGGTT	
	pGADT7-HmoWRKY15-F	
	GGCCATGGAGGCCAGTGAATTCATGGCTGTAGATCTCATGTTGGG	
	pGADT7-HmoWRKY15-R	
	GCAGCTCGAGCTCGATGGATCCTTAAGATGACTCAAGGATCAGAC	
	pGADT7-HmoWRKY16-F	

	GGCCATGGAGGCCAGTGAATTCATGGATGGGTATGAATTTGG
	pGADT7-HmoWRKY16-R
	GCAGCTCGAGCTCGATGGATCCTCATAATAGATCCATTTC
	pGADT7-HmoWRKY17-F
	GGCCATGGAGGCCAGTGAATTCATGGCTCTTGAAGAAAAACTAG
	pGADT7-HmoWRKY17-R
	GCAGCTCGAGCTCGATGGATCCTCAAAATGTAATGGCTGGATATG
	pGADT7-HmoWRKY18-F
	GGCCATGGAGGCCAGTGAATTCATGGACTCATCTAAGTGGATAC
	pGADT7-HmoWRKY18-R
	GCAGCTCGAGCTCGATGGATCCTTAAAGCCATCTCTTAGAATGACT
	pGADT7-HmoWRKY20-F
	GGCCATGGAGGCCAGTGAATTCATGGACGGAGCTCCGGTAGCC
	pGADT7-HmoWRKY20-R
	GCAGCTCGAGCTCGATGGATCCCTAAGGACCCATTAGTATCC
	pGADT7-HmoWRKY21-F
	GGCCATGGAGGCCAGTGAATTCATGGAGGGGGTTGAGGAAGC
	pGADT7-HmoWRKY21-R
	GCAGCTCGAGCTCGATGGATCCTCATGTGTTTGCTGATGGTG
	pGADT7-HmoWRKY23-F
	GGCCATGGAGGCCAGTGAATTCATGGAGAAGGAAGCAGTGTTG
	pGADT7-HmoWRKY23-R
	GCAGCTCGAGCTCGATGGATCCCTACTCTTCAGTCTTCATAATGG
	pGADT7-HmoWRKY24-F
	GGCCATGGAGGCCAGTGAATTCATGACATATGACCCTCATGAA
	pGADT7-HmoWRKY24-R
	GCAGCTCGAGCTCGATGGATCCTCAACAAGAAGGGTGAATTTGC
	pGADT7-HmoWRKY25-F
	GGCCATGGAGGCCAGTGAATTCATGGGGGACAGTGAAGATGC
	pGADT7-HmoWRKY25-R
	GCAGCTCGAGCTCGATGGATCCTCACACAATTTGGCTGTATATAGA
	pGADT7-HmoWRKY26-F
	GGCCATGGAGGCCAGTGAATTCATGGAAGAACTGAAGAAGCAAAA
	pGADT7-HmoWRKY26-R
	GCAGCTCGAGCTCGATGGATCCTTAACGAAGAAGTGACTCTAGAA
	pGADT7-HmoWRKY27-F
	GGCCATGGAGGCCAGTGAATTCATGAACGGCTCAGCTCTTG
	pGADT7-HmoWRKY27-R
	GCAGCTCGAGCTCGATGGATCCTTAAAATAGGAAGGATTGAGGG
	pGADT7-HmoWRKY30-F
	GGCCATGGAGGCCAGTGAATTCATGGAACAAGCCTTGTTGATTACC
	pGADT7-HmoWRKY30-R
	GCAGCTCGAGCTCGATGGATCCTTAATAGAAACTAGGGAA
	pGADT7-HmoWRKY31-F

	GGCCATGGAGGCCAGTGAATTCATGGAAACAACAGGATGGGGAC
	pGADT7-HmoWRKY31-R
	GCAGCTCGAGCTCGATGGATCCCTAGCTACTTGAATTGACTTT
	pGADT7-HmoWRKY32-F
	GGCCATGGAGGCCAGTGAATTCATGGCCTCTAACCCTGGAATTGCT
	pGADT7-HmoWRKY32-R
	GCAGCTCGAGCTCGATGGATCCTCAGCAAGACTTGAGCTCAATTCC
	pGADT7-HmoWRKY33-F
	GGCCATGGAGGCCAGTGAATTCATGTCTTCCTCAGTGGCAAGCATA
	pGADT7-HmoWRKY33-R
	GCAGCTCGAGCTCGATGGATCCTCAATGAAGAAATGAGTCTAAAA
	pGADT7-HmoWRKY34-F
	GGCCATGGAGGCCAGTGAATTCATGTTCCCCTCTTCCATGCCC
	pGADT7-HmoWRKY34-R
	GCAGCTCGAGCTCGATGGATCCTCAGTACAGAAAAGAATCAAAA
	pGADT7-HmoWRKY35-F
	GGCCATGGAGGCCAGTGAATTCATGGTAATGGATAATAAACGTCG
	pGADT7-HmoWRKY35-R
	GCAGCTCGAGCTCGATGGATCCCTACGTGTTGCCATATATGGGAG
	pGADT7-HmoWRKY38-F
	GGCCATGGAGGCCAGTGAATTCATGTTGAAGGGTTCAATTGAACA
	pGADT7-HmoWRKY38-R
	GCAGCTCGAGCTCGATGGATCCTTAAAACTCAAAATTAGGAAAAT
	pGADT7-HmoWRKY39-F
	GGCCATGGAGGCCAGTGAATTCATGGAGGGCCTTGAGGAAGTTAA
	pGADT7-HmoWRKY39-R
	GCAGCTCGAGCTCGATGGATCCTCACGAGTTTGTGGATTGTGAAGG
	pGADT7-HmoWRKY40-F
	GGCCATGGAGGCCAGTGAATTCATGGATTATTCAACGTGGTTAAGC
	pGADT7-HmoWRKY40-R
	GCAGCTCGAGCTCGATGGATCCTTAATCAGTTGAGGTTTGTGCAG
	pGADT7-HmoWRKY41-F
	GGCCATGGAGGCCAGTGAATTCATGGAGTTCAATGGCAACAGTAG
	pGADT7-HmoWRKY41-R
	GCAGCTCGAGCTCGATGGATCCCTATTCCTTAGGCTGAGAGA
	pGADT7-HmoWRKY42-F
	GGCCATGGAGGCCAGTGAATTCATGGAGATTGAGATTTTGGAAG
	pGADT7-HmoWRKY42-R
	GCAGCTCGAGCTCGATGGATCCCTACAGAATTGACAAGTTGAGATT
	pGADT7-HmoWRKY42-1-F
	GGCCATGGAGGCCAGTGAATTCATGGAGATTGAGATTTTGGAAG
	pGADT7-HmoWRKY42-1-R
	GCAGCTCGAGCTCGATGGATCCATTTCCTTTGGCCATTTTTTGCCCA
	pGADT7-HmoWRKY42-2-F

	GGCCATGGAGGCCAGTGAATTCAATCTCCACAATTAGACAGCATC
	pGADT7-HmoWRKY42-2-R
	GCAGCTCGAGCTCGATGGATCCCTACAGAATTGACAAGTTGAGATT
	pGADT7-HmoWRKY42-3-F
	GGCCATGGAGGCCAGTGAATTCATGGAGATTGAGATTTTGGAAG
	pGADT7-HmoWRKY42-3-R
	GCAGCTCGAGCTCGATGGATCCTTGTTCATCATCTCCATCTCGATC
	pGADT7-HmoWRKY42-4-F
	GGCCATGGAGGCCAGTGAATTCATGGAGATTGAGATTTTGGAAG
	pGADT7-HmoWRKY42-4-R
	GCAGCTCGAGCTCGATGGATCCGTCTTCAATCGTAGACTTCTGAAG
	pGADT7-HmoWRKY43-F
	GGCCATGGAGGCCAGTGAATTCATGAAGAAGAAGGGGCAAAAGA
	pGADT7-HmoWRKY43-R
	GCAGCTCGAGCTCGATGGATCCTCATAGGTTTCGCATCCCTGGAG
	pGADT7-HmoWRKY44-F
	GGCCATGGAGGCCAGTGAATTCATGTTGTATTTGTTCAGGATGGAG
	pGADT7-HmoWRKY44-R
	GCAGCTCGAGCTCGATGGATCCCTATTTCTCTTTGGAAGTAAGTGC
	pGADT7-HmoWRKY45-F
	GGCCATGGAGGCCAGTGAATTCATGGTTGCAAAGGAGGAAGG
	pGADT7-HmoWRKY45-R
	GCAGCTCGAGCTCGATGGATCCTCAGTTGGTACCCCGAGTCT
	pGADT7-HmoWRKY46-F
	GGCCATGGAGGCCAGTGAATTCATGGAGAAGATGAAGGGAAGTAC
	pGADT7-HmoWRKY46-R
	GCAGCTCGAGCTCGATGGATCCCTACATATGACGTAAGAGATTATC
	pGADT7-HmoWRKY48-F
	GGCCATGGAGGCCAGTGAATTCATGGAGGAGATGTTGAAGAAAGA
	pGADT7-HmoWRKY48-R
	GCAGCTCGAGCTCGATGGATCCCTAGGTACTGGTGGTGCTGCCG
	pGADT7-HmoWRKY49-F
	GGCCATGGAGGCCAGTGAATTCATGAACATAGAGAACAAGAAGCT
	pGADT7-HmoWRKY49-R
	GCAGCTCGAGCTCGATGGATCCCTAAGAAAATAAAACGTATAC
	pGADT7-HmoWRKY50-F
	GGCCATGGAGGCCAGTGAATTCATGACAACAACATCGACTACTAC
	pGADT7-HmoWRKY50-R
	GCAGCTCGAGCTCGATGGATCCTCATTGCTTCTTAGGGGCTTC
	pGADT7-HmoWRKY51-F
	GGCCATGGAGGCCAGTGAATTCATGGAGCAGTTGGTGCACCTC
	pGADT7-HmoWRKY51-R
	GCAGCTCGAGCTCGATGGATCCCTATGCGGTTGACTGAGGCTGAG
	pGADT7-HmoWRKY54-F

	GGCCATGGAGGCCAGTGAATTCATGGATGAGAAGAAAATGATAAG
	pGADT7-HmoWRKY54-R
	GCAGCTCGAGCTCGATGGATCCCTAAGAACATAGACGATCATAGT
	pGADT7-HmoWRKY56-F
	GGCCATGGAGGCCAGTGAATTCATGGATGACCACCAAGAAGC
	pGADT7-HmoWRKY56-R
	GCAGCTCGAGCTCGATGGATCCTTAGCCTAAACTAGAAAGAAA
	pGADT7-HmoWRKY57-F
	GGCCATGGAGGCCAGTGAATTCATGAGCAATCCGGGTAAACC
	pGADT7-HmoWRKY57-R
	GCAGCTCGAGCTCGATGGATCCTCATAGATTTCGTATTCCAGGAGA
	pGADT7-HmoWRKY58-F
	GGCCATGGAGGCCAGTGAATTCATGGTGGACAACGACGGTGC
	pGADT7-HmoWRKY58-R
	GCAGCTCGAGCTCGATGGATCCCTATGTTATTCTTTCTTCCTTT
	pGADT7-HmoWRKY59-F
	GGCCATGGAGGCCAGTGAATTCATGGATAGAAACTACCAAACCAC
	pGADT7-HmoWRKY59-R
	GCAGCTCGAGCTCGATGGATCCTCATTGCCTTGGAGCGTCATG
	pGADT7-HmoWRKY60-F
	GGCCATGGAGGCCAGTGAATTCATGGATTACTCAAATTCAACAAC
	pGADT7-HmoWRKY60-R
	GCAGCTCGAGCTCGATGGATCCCTAATCACTTGAACTCCGCTGGGC
	pGADT7-HmoWRKY66-F
	GGCCATGGAGGCCAGTGAATTCATGGATAGTTACTCTAGTCCTGAG
	pGADT7-HmoWRKY66-R
	GCAGCTCGAGCTCGATGGATCCTTAAAGGTCCAACTCAAGCTCATC
	pGADT7-HmoWRKY67-F
	GGCCATGGAGGCCAGTGAATTCATGATCGGAGGTACCAAAATGCA
	pGADT7-HmoWRKY67-R
	GCAGCTCGAGCTCGATGGATCCTTAGTCAATGTGCATCGTCCT
	pGADT7-HmoWRKY68-F
	GGCCATGGAGGCCAGTGAATTCATGGCCGAGAAGTACAACAA
	pGADT7-HmoWRKY68-R
	GCAGCTCGAGCTCGATGGATCCTTACTCTTCTTTTGCTGTGGCTTGG
	pGADT7-HmoWRKY69-F
	GGCCATGGAGGCCAGTGAATTCATGGTTATGGAAAATAAATATCG
	pGADT7-HmoWRKY69-R
	GCAGCTCGAGCTCGATGGATCCTCATCCCGTGCTACCACAAA
	pGADT7-HmoWRKY70-F
	GGCCATGGAGGCCAGTGAATTCATGGATCATCTAATTATAGCCC
	pGADT7-HmoWRKY70-R
	GCAGCTCGAGCTCGATGGATCCTCATGCACAGATCAAACTCTGC
	pGADT7-HmoWRKY71-F

	GGCCATGGAGGCCAGTGAATTCATGTCTAGTAATTCAAGGAAGGA	
	pGADT7-HmoWRKY71-R<br>GCAGCTCGAGCTCGATGGATCCCTAAGGGTGCTCTTGCTTCAG	
	pGADT7-HmoWRKY74-F<br>GGCCATGGAGGCCAGTGAATTCATGGAGATTGAAATAGCCAAC	
	pGADT7-HmoWRKY74-R<br>GCAGCTCGAGCTCGATGGATCCTCAGGTGTGAGCGGAATGAGATG	
	pGADT7-HmoWRKY75-F<br>GGCCATGGAGGCCAGTGAATTCATGGAGAATAATCCCATGGCTC	
	pGADT7-HmoWRKY75-R<br>GCAGCTCGAGCTCGATGGATCCTCAAGGAGGTACATAAATCTGC	
	pAbAi-HmocDOPA5GT1-F<br>AAGCTTGAATTCGAGCTCTGACTTGGTCAAAACGCTAAAC	*Sac* I
	pAbAi-HmocDOPA5GT1-R<br>CAGAGCACATGCCTCGAGTCTCTCTTTCTCTCACTTTCTCTTTC	*Xho* I
	62-SK-HmoWRKY30-F<br>TAGAACTAGTGGATCCATGGAACAAGCCTTGTTGATTACC	
	62-SK-HmoWRKY30-R<br>GCTTGATATCGAATTCTAATAGAAACTAGGGAA	
	62-SK-HmoWRKY35-F<br>TAGAACTAGTGGATCCATGGTAATGGATAATAAACGTCGGG	
	62-SK-HmoWRKY35-R<br>GCTTGATATCGAATTCCTACGTGTTGCCATATATGGGAG	*Bam*HI
Dual LUC assay	62-SK-HmoWRKY42-F<br>TAGAACTAGTGGATCCATGGAGATTGAGATTTTGGAAG	*Eco*RI
	62-SK-HmoWRKY42-R<br>GCTTGATATCGAATTCCTACAGAATTGACAAGTTGAGATTATC	
	62-SK-HmoWRKY70-F<br>TAGAACTAGTGGATCCATGGATCATCTAATTATAGCCC	
	62-SK-HmoWRKY70-R<br>GCTTGATATCGAATTCTCATGCACAGATCAAACTCTGC	
	p0800-HmocDOPA5GT1-F<br>CTATAGGGCGAATTGGGTACCTGACTTGGTCAAAACGCTAAAC	*Kpn* I
	p0800-HmocDOPA5GT1-R<br>CGCTCTAGAACTAGTGGATCCTCTCTCTTTCTCTCACTTTCTCTTTC	*Bam*HI
	pBD-62-SK-HmoWRKY42-F<br>CGCCGTCTAGAACTAGTGGATCCATGGAGATTGAGATTTTGGAAG	*Bam*HI
Transcriptional activation analyses	pBD-62-SK-HmoWRKY42-R<br>TCGATAAGCTTGATATCGAATTCCTACAGAATTGACAAGTTGAGATT ATC	*Eco*RI
	pGBKT7-HmoWRKY42-F<br>CATGGAGGCCGAATTCATGGAGATTGAGATTTTGGAAG	*Eco*RI
	pGBKT7-HmoWRKY42-R<br>CAGGTCGACGGATCCCTACAGAATTGACAAGTTGAGATTATC	*Bam*HI

Subcellular localization	pC18-HmoWRKY42-F<br>GGTATCGATAAGCTTATGGAGATTGAGATTTTGGAAG	*Hind*III
	pC18-HmoWRKY42-R<br>CATACTAGTGGATCCCAGAATTGACAAGTTGAGATTATC	*Bam*HI
Gene silence assay	pTRV2-HmoWRKY42-F<br>GCCTCCATGGGGATCCAAGGCAGAGCAGAGCCACGAA	*Bam*HI
	pTRV2-HmoWRKY42-R<br>CTTCGGGACATGCCCGGGGGTTGCGGTCTGGGAGAAGA	*Sma* I
