# Supplementary material for: Genome-Wide Identification of WRKY Gene Family in Pitaya Reveals the Involvement of HmoWRKY42 in Betalain Biosynthesis
Source: Int J Mol Sci. 2022 Sep 12;23(18):10568. doi: 10.3390/ijms231810568 (PMC9502481; doi:10.3390/ijms231810568)
Supplement: Supplementary file 1 [file ijms-23-10568-s001.zip › Supplementary Table S2. Characteristics of the 70 HuWRKY genes in pitaya.pdf]

Supplementary Table S2. Characteristics of the 70 *HuWRKY* genes in pitaya.

Gene names	GenBank number	ORF (bp)	Length (aa)	Molecular weight (kDa)	pI
HuWRKY1	HU10G01510.1	1314	437	48.23	6.35
HuWRKY10	HU01G01634.1	2184	727	78.88	8.04
HuWRKY11	HU05G01931.1	972	323	34.76	9.62
HuWRKY12	HU02G02777.1	690	229	26.31	9.11
HuWRKY13	HU09G01343.1	717	238	27.22	9.42
HuWRKY14	HU05G01710.1	1299	432	47.41	5.49
HuWRKY15	HU06G00411.1	978	325	35.75	9.72
HuWRKY16	HU05G00106.1	801	266	29.54	6.55
HuWRKY17	HU05G00484.1	792	263	29.36	9.39
HuWRKY18	HU09G01339.1	948	315	35.63	8.24
HuWRKY2	HU03G01517.1	2241	746	81.04	5.85
HuWRKY20	HU08G00283.1	1785	594	63.51	6.26
HuWRKY21	HU01G02705.1	1047	348	38.65	9.51
HuWRKY22	HU07G00041.1	1080	359	38.46	5.84
HuWRKY23	HU04G01891.1	1146	381	40.96	5.5
HuWRKY24	HU10G00045.1	585	194	22.74	5.64
HuWRKY25	HU08G01332.1	2367	788	85.71	5.3
HuWRKY26	HU02G02539.1	1116	371	41.39	7.61
HuWRKY27	HU06G02562.1	1221	406	44.65	5.6
HuWRKY28	HU04G00262.1	762	253	27.43	5.95
HuWRKY29	HU11G01744.1	1119	372	39.55	6.24
HuWRKY3	HU10G00405.1	1518	505	54.91	6.41
HuWRKY30	HU04G00301.1	1062	353	39.18	5.67
HuWRKY31	HU08G01942.1	1836	611	65.61	6.44
HuWRKY32	HU09G01332.1	1683	560	60.74	8.87
HuWRKY33	HU04G01010.1	1626	541	59.92	6.18
HuWRKY34	HU02G01423.1	1359	452	49.79	7.17
HuWRKY35	HU03G01848.1	879	292	32.1	6.21
HuWRKY36	HU10G00322.1	1455	484	52.59	8.44
HuWRKY38	HU07G00077.1	1098	365	40.84	6.05
HuWRKY39	HU04G01916.1	1056	351	38.56	9.59
HuWRKY4	HU06G01192.1	1296	431	47.41	8.47
HuWRKY40	HU01G00584.1	1065	354	38.78	6.91
HuWRKY41	HU05G00064.1	1092	363	40.39	6.13
HuWRKY42	HU01G01699.1	1491	496	54.35	6.51
HuWRKY43	HU10G00427.1	576	191	21.62	9.13
HuWRKY44	HU06G02430.1	1374	457	50.89	9.14
HuWRKY45	HU01G01395.1	1527	508	57.08	5.96
HuWRKY46	HU11G01694.1	1098	365	40.54	6.86
HuWRKY47	HU08G00218.1	1473	490	53.02	6.07
HuWRKY48	HU06G02188.1	1209	402	43.74	5.91
HuWRKY49	HU09G01312.1	441	146	17.07	9.37

HuWRKY50	HU09G01525.1	516	171	19.05	5.82
HuWRKY51	HU11G00035.1	1146	381	41.06	9.51
HuWRKY53	HU06G02594.1	1335	444	50.22	6.95
HuWRKY54	HU09G01148.1	1053	350	40.03	6.49
HuWRKY55	HU06G00952.1	1026	341	37.86	6.05
HuWRKY56	HU11G01740.1	660	219	25.07	6.91
HuWRKY57	HU02G00385.1	951	316	34.92	6.34
HuWRKY58	HU02G00374.1	1476	491	53.62	8.09
HuWRKY59	HU02G03012.1	528	175	20.34	5.19
HuWRKY6	HU10G01980.1	1884	627	68.01	6.45
HuWRKY60	HU03G00514.1	1074	357	39.04	6.86
HuWRKY61	HU04G02201.1	1896	631	67.58	6.24
HuWRKY62	HU07G01666.1	1041	346	39.46	6.16
HuWRKY63	HU05G01336.1	606	201	22.44	6.78
HuWRKY64	HU08G01085.1	894	297	33.4	7.2
HuWRKY65	HU08G01913.1	861	286	31.46	6.08
HuWRKY66	HU01G02778.1	684	227	25.43	9.2
HuWRKY67	HU10G01349.1	153	50	5.73	10.12
HuWRKY68	HU02G03246.1	1185	394	42.51	5.95
HuWRKY69	HU01G00960.1	855	284	31.13	5.2
HuWRKY7	HU04G00611.1	1035	344	36.98	9.7
HuWRKY70	HU02G02628.1	939	312	34.82	5.99
HuWRKY71	HU08G01835.1	1020	339	37.56	7.26
HuWRKY72	HU06G01151.1	1719	572	62.84	5.92
HuWRKY74	HU03G01032.1	1074	357	39.99	9.84
HuWRKY75	HU01G02174.1	525	174	20.31	9.28
HuWRKY8	HU02G00037.1	792	263	30.38	9.05
HuWRKY9	HU09G01313.1	936	311	35.17	8.43
