# Supplementary material for: Genome-Wide Identification of WRKY Gene Family in Pitaya Reveals the Involvement of HmoWRKY42 in Betalain Biosynthesis
Source: Int J Mol Sci. 2022 Sep 12;23(18):10568. doi: 10.3390/ijms231810568 (PMC9502481; doi:10.3390/ijms231810568)
Supplement: Supplementary file 1 [file ijms-23-10568-s001.zip › Supplementary Table S3. Gene duplication and synteny analysis of WRKY genes between pitaya and sugar beet.pdf]

Supplementary Table S3. Gene duplication and synteny analysis of WRKY genes between pitaya and sugar beet.

Number	*Hylocereus undatus*		*Beta vulgaris*	
	Chromosome location	Gene names	Chromosome location	GenBank number
1	Chr02	HuWRKY8	Chr06	0KMT07420
2	Chr05	HuWRKY11	Chr06	0KMT09751
3	Chr06	HuWRKY15	Chr06	0KMT09751
4	Chr10	HuWRKY24	Chr06	0KMT07420
5	Chr10	HuWRKY3	Chr06	0KMT06985
6	Chr10	HuWRKY36	Chr06	0KMT07123
7	Chr01	HuWRKY21	Chr05	0KMT10289
8	Chr04	HuWRKY23	Chr05	0KMT09995
9	Chr04	HuWRKY61	Chr05	0KMT10375
10	Chr04	HuWRKY30	Chr05	0KMT11948
11	Chr05	HuWRKY16	Chr05	0KMT12028
12	Chr10	HuWRKY6	Chr08	0KMT04131
13	Chr02	HuWRKY34	Chr07	0KMT05423
14	Chr02	HuWRKY70	Chr07	0KMT06512
15	Chr02	HuWRKY26	Chr07	0KMT06634
16	Chr09	HuWRKY54	Chr07	0KMT06512
17	Chr02	HuWRKY59	Chr02	0KMT17894
18	Chr06	HuWRKY4	Chr02	0KMT17318
19	Chr09	HuWRKY50	Chr02	0KMT17894
20	Chr07	HuWRKY38	Chr01	0KMT20339
21	Chr07	HuWRKY22	Chr01	0KMT20406
22	Chr11	HuWRKY46	Chr01	0KMT20339
23	Chr11	HuWRKY56	Chr01	0KMT20402
24	Chr11	HuWRKY29	Chr01	0KMT20406
25	Chr01	HuWRKY10	Chr04	0KMT12903
26	Chr03	HuWRKY2	Chr04	0KMT14758
27	Chr08	HuWRKY25	Chr04	0KMT14758
28	Chr08	HuWRKY20	Chr04	0KMT12903
29	Chr02	HuWRKY68	Chr03	0KMT16293
30	Chr06	HuWRKY53	Chr03	0KMT16079
31	Chr06	HuWRKY27	Chr03	0KMT16161
32	Chr01	HuWRKY40	Chr09	0KMT01048
33	Chr03	HuWRKY60	Chr09	0KMT01048
