# Supplementary material for: Genome-Wide Identification of WRKY Gene Family in Pitaya Reveals the Involvement of HmoWRKY42 in Betalain Biosynthesis
Source: Int J Mol Sci. 2022 Sep 12;23(18):10568. doi: 10.3390/ijms231810568 (PMC9502481; doi:10.3390/ijms231810568)
Supplement: Supplementary file 1 [file ijms-23-10568-s001.zip › Supplementary Table S4. Gene duplication and synteny analysis of WRKY genes between pitaya and Arabidopsis.pdf]

Supplementary Table S4. Gene duplication and synteny analysis of WRKY genes between pitaya and Arabidopsis.

Number	*Hylocereus undatus*		*Arabidopsis thaliana*	
	Chromosome location	Gene names	Chromosome location	GenBank number
1	Chr04	HuWRKY28	Chr01	AT1G64000.1
2	Chr10	HuWRKY6	Chr01	AT1G62300.1
3	Chr10	HuWRKY43	Chr01	AT1G69310.1
4	Chr10	HuWRKY36	Chr01	AT1G69810.1
5	Chr10	HuWRKY3	Chr01	AT1G13960.1
6	Chr05	HuWRKY63	Chr02	AT2G40740.3
7	Chr07	HuWRKY38	Chr02	AT2G46400.1
8	Chr10	HuWRKY3	Chr02	AT2G03340.1
9	Chr11	HuWRKY56	Chr02	AT2G46130.1
10	Chr11	HuWRKY46	Chr02	AT2G46400.1
11	Chr03	HuWRKY74	Chr03	AT3G04670.1
12	Chr05	HuWRKY63	Chr03	AT3G56400.1
13	Chr01	HuWRKY10	Chr04	AT4G26640.2
14	Chr04	HuWRKY30	Chr04	AT4G23810.1
15	Chr04	HuWRKY30	Chr04	AT4G11070.1
16	Chr06	HuWRKY27	Chr05	AT5G52830.1
