# Supplementary material for: Genome-Wide Identification of WRKY Gene Family in Pitaya Reveals the Involvement of HmoWRKY42 in Betalain Biosynthesis
Source: Int J Mol Sci. 2022 Sep 12;23(18):10568. doi: 10.3390/ijms231810568 (PMC9502481; doi:10.3390/ijms231810568)
Supplement: Supplementary file 1 [file ijms-23-10568-s001.zip › Supplementary Text S1. cDNA sequences of the 70 HuWRKY genes in pitaya.pdf]

Supplementary Text S1. Summary of WRKY members information in pitaya. Yellow highlights indicates specific primers for full length cloning of cDNA sequences. Underlining indicates sequence of *HmoWRKY* genes primers for real-time PCR. The downstream primer is the reverse complementary sequence.

>*HuWRKY1*

ATGGTTGCAAAGGAGGAAGCTCATGTGGTTGTTGCTCCTGAGGATAACTTGCAACACAGACCAAGCAC
TGATTCTGCAGTGCATGCTTCCCACACTGACCAAGAAGAGAAGACACCTATTGTCCCAGAGAAACCAT
CAGAAGACGGATATAATTGGAGAAAATATGGCCAAAAGAATGTCAAAGGAAATGAATACATACGAAGC
TATTACAAGTGTTCATATCCAAACTGCCAGGTGAAAAAACAAGTGGAGCGCTCGCCGACTGGACGGAT
TACTGATGTTACTTACTTGGGGAAGCATGACCATGCTAAACCTCGCTCTGAAACCCAGGAAGCTGTCCC
CTCTTTTCAGTCGGTACACTTACAGAAGCCAGATTGTCCTGTATCCACTGTTGAAGACAAGCCATCTGA
TGCATGTGCCCTCACAACTGATAATGTTCAACCTCAGCCAATGGATGTATGTAACCATCCAATAGTTGAT
GAGGGTACTTCTACGACCTGTCTTCCAGCGGCAGATCTAGCCAAGGATGTTCATAATGAAAATATGATC
CTAAAGAGACGGAAGAAAAGCATCGATGGTGCTGATATCATAACAGAAAAACCTACTCATGAATCACG
TCTTGTTGTCCATACGGTTAGTGAGTTTGATATCGTAAATGACGGGTACAGGTGGCGCAAATACGGACA
GAAAATGGTCAAAGGCAATCCTAATCCACGGAGTTACTACAGGTGCTCTACAGCAGGATGCCCAGTCA
AGAAACATGTCGAAAGGGCTTCTCATGATCCGAAAGTCGTCATCACCACATATGAGGGTCAACATGATC
ACAGTATACCTCCCATCAGGACCGTCGTCCCCCAGAGTGGAGCTCCATCTGGTGGCGGGATTGAATTGA
ACGGTGTCAGTGGGACCAAGTCACAAGAGAGTGGTCAGAAGGGTTCAACAATGTGTGATGCCGTTGG
TTCCACAGCAAAAGAAGAAATCAACACCAAAGCAGCAGTTAAGATCGAGCCTGAAGTTTCACCTAGC
CTGCAAGAACTGCAGCCCAAATCCAAAAGAAACAATCAAATTGAACATACTAGTCATGCAATTTCAGG
TAGTGAGCAGCAAATGCAGGACGATCCCATTTCCAGTGTGTCAAATTCTGATAATGTTGATCATCAAAG
TGTCAAACAAGAAAAAGGGGCCACAGTTCAACAAGTAAAGATGGGGGTTGTCTCAACCCAGGAGCAG
AGGCCTGATCCACTTGTTGACAGGTCTCATCTTGAGACGAGATCAAGTGGCCAAGAAAGGCCTGCTGA
GCCTGTTAATTGCTGA

>*HmoWRKY1*

ATGGTTGCAAAGGAGGAAGCTCATGTGGTTGTTGCTCCTGAGGATAACTTGCAACACAGACCAAGCAC
TGATTCTGCAGTGCATGCTTCCCACACTGACCAAGAAGAGAAGACACCTATTGTCCCAGAGAAACCAT
CAGAAGACGGATATAATTGGAGAAAATATGGCCAAAAGAATGTCAAAGGAAATGAATACATACGAAGC
TATTACAAGTGTTCATATCCAAACTGCCAGGTGAAAAAACAAGTGGAGCGCTCGCCGACTGGACGGAT
TACTGATGTTACTTACTTGGGGAAGCATGACCATGCTAAACCTCGCTCTGAAACCCAGGAAGCTGTCCC

CTCTTTTCAGTCGGTACACTTACAGAAGCCAGATTGTCCTGTATCCACTGTTGAAGACAAGCCATCTGA

TGCATGTGCCCTCACAACTGATAATGTTCAACCTCAGCCAATGGATGTATGTAACCATCCAATAGTTGAT

GAGGGTACTTCTACGACCTGTCTTCCAGCGGCAGATCTAGCCAAGGATGTTCATAATGAAAATATGATC

CTAAAGAGACGGAAGAAAAGCATCGATGGTGCTGATATCATAACAGAAAAACCTACTCATGAATCACG

TCTTGTTGTCCATACGGTTAGTGAGTTTGATATCGTAAATGACGGGTACAGGTGGCGCAAATACGGACA

GAAAATGGTCAAAGGCAATCCTAATCCACGGAGTTACTACAGGTGCTCTACAGCAGGATGCCCAGTCA

AGAAACATGTCGAAAGGGCTTCTCATGATCCGAAAGTCGTCATCACCACATATGAGGGTCAACATGATC

ACAGTATACCTCCCATCAGGACCGTCGTCCCCCAGAGTGGAGCTCCATCTGGTGGCGGGATTGAATTGA

ACGGTGTCAGTGGGACCAAGTCACAAGAGAGTGGTCAGAAGGGTTCAACAATGTGTGATGCCGTTGG

TTCCACAGCAAAAGAAGAAATCAACACCAAAGCAGCAGTTAAGATCGAGCCTGAAGTTTCACCTAGC

CTGCAAGAACTGCAGCCCAAATCCAAAAGAAACAATCAAATTGAACATACTAGTCATGCAATTTCAGG

TAGTGAGCAGCAAATGCAGGACGATCCCATTTCCAGTGTGTCAAATTCTGATAATGTTGATCATCAAAG

TGTCAAACAAGAAAAAGGGGCCACAGTTCAACAAGTAAAGATGGGGGTTGTCTCAACCCAGGAGCAG

AGGCCTGATCCACTTGTTGACAGGTCTCATCTTGAGACGAGATCAAGTGGCCAAGAAAGGCCTGCTGA

GCCTGTTAATTGCTGA

>*HuWRKY2*

ATGGAAGAGAGTGGTGATGGGCTTGAAGAGTGGAGGGATCTTGTGATGAGCCCTAACCCTAAAGGGTT

GTTCTCAGGTTTTACATCTGAGCCATGTGTTAGCAAATCAATCGATGATGCTTTGATTGGTGGGGAGGGT

ATTTTGGGTGTTGATCATGAAGAAGTTGAGAATTCAATGTTGGATTCAAAGAGGAAAGTGCAGGGTACT

AGTGAAAATGGGTTGAATTCTTCTTCCATTGGCATGGGTGATTCTGCTGGCATTAAGACAGGAGGGAGC

ATAGCAGAGAGAAGGGCAGCTACATTTGGATTCAATGCTGCTAAAATCAGCAATCCTCGGTTTAGGTCT

AGTAGCATGTTGCCTTCTCCAGGCGTTCGATCACCTTATCTGACCATACCGCCTGGCATTAGCCCCACTG

CATTGCTAAACTCTCCGATTATGGTATCCAATCCACAGCCTTCTCCAACCACGGGGAGTTTTGCATCACC

TCCTATGGACAGGGGCAATGAAATGTTGATTTCAGCATCTCATGCAGAAATGGACAAGGTTAGAGATGA

TGGTTTATCTTCCATGTTTAAGCCCAGAGTTGATCCCATTCCTATGGATTGTGGCCTGGCTGCAGAAAAT

CAGGTTTGTTCTACTAGTGGTCTGGCTCAGAGTTTGGTTGCTTATCTTCAACATCAAAGTCTTACATCCA

TGAATCTGCCAACGGACATCAAATTTCCCACGGAAATCCCTACAGAAGTTACTGTGGACAATGACCTTG

CTGGCTTACCACCAGAGGATTCAGGAGCCCACAATTCTTTGGACGGGGACCAGAAAGCTCCAACAAGT

ACACTAAGAACTGCAGAGGACGAATACACTTGGAGGAAATATGGCCAGAAGCAAGTCAAGGGTAGTG

AATATCCTCGGAGTTACTATAAATGCACCCATCAAAACTGTGAAGTAAAGAAGAAGGTAGAACATTCTC

ATGATGGCCAGATAATAGAGATCATCTATAAGGGTACCCATAATCATCCAAAACCTCAACCTAGTCGGAA

ATCGACCTCATCATTCAGTGAGATATCTGACGCAAATGATGCTAAGGGATCTTCTATTAAAGTTGATGGT

GGCTTAGTCTGGAGAAGTATAGAAAGTGGATCTAGGCACACTAAGATGGCATCAGATTGGAGAGCTGA

TGGCTTGGAGAGGACATCTTCAACTTCCGTTGTCACTGAAGTTTCTGATCTACTGTCAACCACTCAAGG

GAAGCTGATGGGTATGCATGAATCAGCAGGAACTCCAGAGTTCTTGTCAACACTGGCCATCCATGAGG

ATGATGAATCAGAAGCAGCAGCACCACAAGGAAGGTTTTCATTTGAAGATGATGGTGATAATGAACCA

GATTCAAAAAGAAGGAGGAAGGAGGCTTGTTTCATTGAAACGACTTTAGGTTCCACGGCTGTTCGTGA

ACCAAGAGTAGTTGTCCAAATAGAGAGTGATGTGGATATTCTTGATGATGGGTATCGTTGGCGAAAATA

TGGGCAAAAAGTCGTCAAGGGAAATCCCAACCCTAGGAGCTACTACAAATGCACCAATGCTGGATGCA

CTGTCAGGAAGCACGTGGAGAGAGCATCAGATAATTTGAAATGTGTGATAACGACATATGAAGGGAAA

CACAATCATGCAGTGCCAGTAGCTAGAAACAGCAGCCACGCAAATTCAGGCAGCAGAAGTTTACCCCC

TCCCGGGACTAATGCCCAGTCTGCCCTGACATTACCCAGACAACCCCATCTTCCAAAGGCTGAACCTCA

TGTCCACGAACTTCCCCTCCATTTTGAGGAAAAAAAGGAGTGCAGCAATGACTTTCTAAGACCAAACT

TCCTAGGAAGTTTAGGAGGTGGAACTACCCCATTTTACCAAATGAAATTCCCTATTTTCTGCAGTGCAA

CACCATCTGGTCCCTTTGGTTTGAGTAATCACCATCCAGGTTATTCTGGTGGCATCGTACCACTCATCCC

AGAAGTTCCTATCCCATTGCCAGTAGGGCTTGCTCAATCTCCAAGTTTGTCCATCACTAATTACAACAAT

TGTGGAAGATCGCTGTCTTCAGTTCAGCCCTTACATACTGCACACCAACTGGAGGAAAACAATGTCAA

ACACAGAGATGACTTGATCTATGATACAGGAATGGAGAATACCGACCATGCAAACCCTTCATCATCCTC

CATATACAACCATATTGCAAGGTCCTTTCCATTGTGA

>*HuWRKY3*

ATGCCGGAAAACGATGCCGTGACTTCCACGGAACCGCCGCCGGCGCCGCGGATGGCGGTGGGGCAGC

TGCAGCCACCGACTATTACACTGCCGCCGCGTGGTGGGGTCGACTCCATTTTTTCTGGGTCCGGGTTCG

GAGCTAGCCCAGGCCCGATGACCCTGGTCTCCAGCTTCTTCTCTGAGAATGACCCGGATTCTGATTGTC

GTTCTTTCTCTCAGCTCTTAGCTGGCGCCATGGCTTCTCCCCGACCGGCTTCCGAGAACAGCTCTAGTG

AACCGGACCATCTCCGGTTCAGGCAGAGCAGGCCGGCCGGTCTGGTGGTTTCTCAGCAGCAAGGGGT

TTTCACCATTCCGCCCGGGCTTAGTCCTGCTAGCTTGCTCGATTCTCCTGGTTTATTCCCTGGTCAGGGG

CCATTTGGGATGTCTCACCAGCAAGCGCTAGCTCAAGTTACGGCTCAAGCTGTACAAGCACAAGCTAA

CTTGCATGTGCAAGGCAATTTTCCATCTTCCCTCGGTATAGGTCTTCCGGGATCATCTCCATTTCCAGCC

TTTATCTCTGAGTCAATGCCACAAAATGTGACAAGTTCTGCGCTAGATTCTACCGAGATAAAAGAACCG

GCACAGTACTCTGGCATGGACCAAAGATCACAGCCTTCTTCCATGATTGTTGATAAGCCAGCTGATGAT
GGCTATAATTGGAGGAAATATGGGCAGAAGCAAGTTAAAGGGAGTGAATTTCCCCGAAGTTATTACAAA
TGTACACACATTAATTGCCCTGTCAAGAAGAAGGTGGAGCGTTCTCTTGATGGCCAAGTGACCGAGAT
AATCTATAAGGGCCAGCACAATCATCCGCCACCACAAAAACGTTCTAAAGAGAGCGGAAATTCTAATG
GAAACTTGGGAAGTCAAGGAAACCCTGAATTAAACATGAATAGCACGAATGAGGGCATGACTTACTCA
TTGTCTAGAAGGGATCAGGAATCTAGCCAAGCTACAGCAGAGCATTTGTCTAGTGATGAGGAGGAAGT
GGGCAATGATGAAACTAGAGAATCTGAGGATGAACCTGATGCCAAGAGAAGGAACACAGAACTCCGG
GTACCAGAGCAAGCTGTTTCACATAGGACAGTCACTGAACCCAAGATTATTGTTCAAACAACTAGTGA
AGTTGATCTTTTGGACGATGGTTATAGGTGGCGCAAGTATGGACAAAAAGTTGTCAAAGGAAATTCATA
TCCGAGGAGCTATTACAAATGCACTCACCCAGGGTGTAATGTTCGTAAGCATATTGAGCGGGCAGCCTC
AGACCCTAAAGCAGTGATAACAACATACGAGGGGAAGCACAGTCACGATGTTCCAGCTGCTAAGACG
AGCAGCCATAACACATCCAACAGCAGCTTTTCACAGCCAAGACCGCCTAGTTTGGCAGCGAATAATAA
CTCCTATGTTGGGAAGACGGATTTTACAAGTAATCAACAGCCTGTAGCCCTTCTCCATTTAAAACAAGA
GAGAATAACATAG

>*HmoWRKY3*

ATGCCGGAAAACGATGCCGTGACTTCCACGAAACCGCCGCCGGCGCCGCGGATGGCGGTGGG
GCAGCTGCAGCCACCGACTATTACACTGCCGCCGCGTGGTGGGGTCGACTCCATTTTTTCTGGG
TCCGGGTTCGGAGCTAGCCCAGGCCCGATGACCCTGGTCTCCAGCTTCTTCTCTGAGAATGAC
CCGGATTCTGATTGTCGTTCTTTCTCTCAGCTCTTAGCTGGCGCCATGGCGTCTCCCCGACCGG
CTTCCGAGAACAGCTCTAGTGAACCGGACCATCTCCGGTTCAGGCAGAGCAGGCCGGCCGGT
CTGGTGGTTTCTCAGCAGCAAGGGGTTTTCACCATTCCGCCCGGGCTTAGTCCTGCTAGCTTGC
TCGATTCTCCTGGTTTATTCCCTGGTCAGGGGCCATTTGGGATGTCTCACCAGCAAGCGCTAGC
TCAAGTTACGGCTCAAGCTGTACAAGCACAAGCTAACTTGCATGTGCAAGGCAATTTTCCATCT
TCCCTCGGTATAGGTCTTCCGGGATCATCTCCATTTCCAGCCTTTATCTCTGAGTCAATGCCACA
AAATGTGACAAGTTCTGCCCTAGATTCTACCGAGATAAAAGAACCGGCACAGTACTCTGGCAT
GGACCAAAGATCACAGCCTTCTTCCATGATTGTTGATAAGCCAGCTGATGATGGCTATAATTGG
AGGAAATATGGGCAGAAGCAAGTTAAAGGGAGTGAATTTCCCCGAAGTTATTACAAATGTACA
CACATTAATTGCCCTGTCAAGAAGAAGGTGGAGCGTTCTCTTGATGGCCAAGTGACCGAGATA
ATCTATAAGGGCCAGCACAATCATCCGCCACCACAAAAACGTTCTAAAGAGAGCGGAAATTCT
AATGGAAACTTGGGAAGTCAAGGAAACCCTGAATTAAACATGAATAGCACGAATGAGGGCATG

ACTTACTCATTGTCTAGAAGGGATCAGGAATCTAGCCAAGCTACAGCAGAGCATTTGTCTAGTG
ATGAGGAGGAAGTGGGCAATGATGAAACTAGAGAATCTGAGGATGAACCTGATGCCAAGAGA
AGGAACACAGAACTCCGGGTACCAGAGCAAGCTGTTTCACATAGGACAGTCACTGAACCCAA
GATTATTGTTCAAACAACTAGTGAAGTTGATCTTTTGGACGATGGTTATAGGTGGCGCAAGTAT
GGACAAAAAGTTGTCAAAGGAAATTCATATCCGAGGAGCTATTACAAATGCACTCACCCAGGG
TGTAATGTTCGTAAGCATATTGAGCGGGCAGCCTCAGACCCTAAAGCAGTGATAACAACATACG
AGGGGAAGCACAGTCACGATGTTCCAGCTGCTAAGACGAGCAGCCATAACACATCCAACAGC
AGCTTTTCACAGCCAAGACCGCCTAGTTTGGCAGCGAATAATAACTCCTATGTTGGGAAGACG
GATTTTACAAGTAATCAACAGCCTGTAGCCCTTCTCCATTTAAAACAAGAGAGAATAACATAG

>*HuWRKY4*

ATGGCGTCCAACTTATTTTCCGACGGATTTTCTGGCTCATTTTCCGACCTCCTCAACGCAGAGATGAAC
GCTCCGGTGCCGCCTTCCGGCCTGGGGCTTAGCTTTCTGTCGGAAAAAGCCGAGAATAGCGCCGGCGC
CGGTGGTGGCTCATCTGGGTATAGGCAGAGTAAGCCGGCGAATCTGATTTTACCGGTGAACGTCTCGCC
GGCGTTTTCTGTTTCGCCGGGGATTAGCCCCTCTCTTTTTCTCAACTCGCCCACCTTCTTACAGTCCGCT
CTGAACGCAACATATGGTACGTCTCCCCAACAGGTACTGCAAGTAACTGCTCAAGCAGCACTTTCCAAT
TCCCATTTGGGCATCCAACCTGACTTCTACCCATCTTCATTAAGCCCAACCCCGCTGCATCCATCTATGA
CTTACCAGTCGTCCACTTTTAATACTATATCGCGTATTCCCGAACGCCCAAATGCCCTCCACCACGAAAA
GAGGCACCAGAGCTTCCCTGCACCCGTTGACAAGCCGGCTGATGATGGGTACAACTGGAGGAAATACG
GGCAAAAACAAGTCAAGGGAAGTGAATACCCTAGAAGCTATTATCGATGTACTAGTCCTAATTGCCCTG
TTAAGAAAAAGGTGGAGCGGTCCTTCGATGGGCATATAACTGAGATCATCTACAAAGGCCAGCATAATC
ATGATCCCCCTCCATCTGGAAGAAGAGGAAAAGACAACAATGCTTTGCTTGATCATGGTGGGTCTCAG
GGCCAGATGGGTAACTTCCATTGGTCAACCAGGCTAGGGAGGGACCATGATTCAGCCCAAGCCACATC
GATGCAGCTAAATGGCCCAAGTGATAGTGAAGAATCGGGTGAAAATTATGATCTAGCTGATGAGGAAGA
TGATGAGGACAAGCCAAATGCCAAGAGAAGGCAAGTATATTATTCAACAAATGTTCTTCAAGGACATAT
TTTGGTTTTTTTAAGACCATCTGACGTCCCTTTCACGCACAGGACAATGACGGAGCCAAAAATTGTTGT
GCAGACGAGGAGTGAGGTCGATCTTTTGGACGATGGCTATAGATGGCGCAAGTATGGGCAGAAGGTCG
TCAAAGGCAATCCTAATCCAAGGAGCTATTATAAGTGTACCACTGCAGGGTGCAAAGTCCGCAAACAT
GTAGAGAGGGCATCTACTGACCCAAGAGCGGTGATAACTACCTATGAAGGGAAGCACAACCATGATGT
GCCAACTGCTAGAGGCAGCAATGCTAACACCATTAACTCCCAAAGTTCAACAGACTAG

>*HuWRKY6*

ATGGACGGTGGAGGATGGGCTTCATCACTTGCTCTTCAATCTGATCATCATCATCATCATCATCAAAGAA
ATCTTACTTCTTTCTTAGGTAAAACAAACCCTACTACTCCTACTACTACAAGGATCAGGATGAGCAATAA
TCAGACGTTCCCAGGCGTAGAATTCCCAGTGAACCTCAACCTCAGAGGGGATCATGATCATGATTATGA
TCATGATCACAAACATGAGGATGAGGATCATGAGGATCGTAAAGTGGCGGTTGGTGAAGTAGATTTCTT
CTCTGATCATCATCAGAAGAATAACAATAATAATAATAAGAGCAGTTTGGTCAGTAATAATAGCCGT
GTGATCAAGGAAGAGAATTCTCCTCGTGATCATCATAATCGTGCTCCTGCTCCACCTGATCATCCCTCCA
CCAGACCTCACCTCGATGTTAATACTGGTCTACGTCTCCTCACAACTAATACGGGAAGCGATCAATCTA
CTATAGATGATGGTACTTCTTCGGATGCAGAAGATAAACGAGCTAAAAATGAGCTTGTTGTGCTTCAAG
CCGAGCTCGAAAGGATGAATGCTGAAAATCAAAAATTAAAGACCATGTTATCTCAAATAAGCCAGAATT
ATAGCACTCTTCAAATGCACCTCGTGACATTGATGCAGCAACAACAACAACAACAAAAGAGTACGCAA
AATGAGGGAATGCAAGGAAAGGGGGATCAAGATAATAAGCAAATAGTGCCCCGCCAATTCTTAGATTT
AGGCCCAAGTGAGAACAACGAGTCGATAGATGGAATGACATCTGAGGATGAGAGAACCGTAGATCGCT
CGGGCTCTCCTAGGAAGAGGATAAGGGAGGATAGTCCTGAAGACTCGGACCCTCAAGGGTGGGTTTCC
AGTAAGGCACCCAAGTTGGGTGCCTCTAATATGGGTGCCCCTAACAAGGGCACTAGCCCCCCAGCTGA
ACAAGCACCCGCTGAGCTAGCCACAATGAGGAAGGCTCGTGTCTCAGTTCGAGCTCGATCCGAAGCA
CCTATGATAAGTGATGGATGTCAATGGAGAAAATATGGACAAAAGATGGCAAAGGGGAATCCTTGCCCT
AGAGCTTATTATCGGTGCACGATGGCTATTGGTTGTCCGGTTCGCAAACAAGTTCAAAGATGTGCGGAG
GACAGGTCAATATTGATCACAACCTATGAAGGCAACCACAGTCATCCACTGCCACCGGCAGCCATGGC
AATGGCCTCAACAACCTCCGCGGCAGCGAGCATGCTCCTATCGGGCTCGATGCCTAGTGGGGACGGGC
TCATGAATCCAAACTTTCTCGCTAGGACCATCCTCCCATGCTCATCAAGCCTGGCCACAATCTCAGCCT
CAGCCCCCTTCCCGACTGTGACCCTAGACCTAACCCACTCACCCAACCCTCTCCAAATACAAAGGCCAT
CAGTACTGCACCAACCAGCCAGCCACTTCCAAGTCCCCTTCCCCCAACCCCAACCCCAACCCACCGCC
GGTCACGTGTCGATCCCTCCCAACTTTATCAACTCAATGCCTCAAATATTTGGGCAAACCCTATTTGCCA
ACAATATGAACCAATCCAAATTCTCAGGGTTACAAACTTCATCCTCCCAAGGCCTTGATGCTATGTTACA
TGTTGGCGGGACCGGATTCCCCGACACCTTGAGCGCCGCCACCGCCGCCATTGCCTCCGATCCCAACT
TCACGGCAGCCCTTGCGGCCGCCATCTCATCGATCATCGGTGGGGTCCGCCCTGGGGAAAACAATAAT
GCCAATCATTCTTCAGGTGGTGCAAATACTACTAGTGGTGATAACAACAAGGGTAATAATGTGAATTCTA
GTAGTGGCATGCAACAACAACAATAA

>*HuWRKY7*

ATGGCAGTGGAGCTTATGGCAAGCTCCTCCTCTTACAAGGAGAGCTTCGCAGCTCTAATGGAGGAAAA
CGCGGTCAGAGAAGCCGCCGCGGCCGGCCTTCAGAGCGTCGAGAAGCTCATCAAACTCATCTCTCAGA
TGGACAACCAGCACCACACCACCACCTCCTCACTCAACAACAACAGCAACGACTCTAGAAACTCCTC
GATCCGATTCGATGATTCCGATTACAAGGCGGTGGCGGACGTCGCTGTCAACAGGTTCAGGAAGGTAA
TCTCTCTCCTCGACCGCACTCGCACCGGCCACGCGCGCTTCCGGCGCGGTCCGCTCTCTTCACCGCCTC
CTCCTCCGCCGCCACTTCCGCTGAAGCCGATCATCCACGGCGGCGCAGGCTCGGCCTTCACGAGAGTC
CACGGCGGTGGAGCTTCTCAGATCCAGCGCCTGCCGCCGCTTCCGCAGCCGAAGACGGTGGCGGAGA
AAAAGGAATCGGCATCGACGACGATCCATTTCTCGACGTCACCGCCGATGTCCGCCGCGAACTCGTAC
ATCTCGTCGTTGACCGGCGACACCGAGAGTTTGCCGGCGTCGATGTGCTCCGGCGGGTTTCAGATTAC
GAATTTGTCGCAGGTTTCTTCCGCCGGAAAACCGCCTCTCTCGTCTTCGTCGTTGAAGCGGAAGTGTA
ACTCCATGGATGACTCCAAGTGTGGCGGTTCTTCTGGACGTTGCCATTGCTCTAAGCGAAGGAAATCA
AGGACAAAGAGGGTGGTAAGAGTACCAGCAATAAGCTTGAAGATGGCGGATATTCCACCGGATGATTA
CTCCTGGAGGAAGTACGGTCAGAAGCCAATCAAGGGGTCTCCTCATCCAAGGGGATATTACAAGTGTA
GTAGCGTGCGGGGGTGTCCAGCAAGGAAGCATGTGGAGCGTGCATCGGACGATCCATCGATGCTGATC
GTGACGTACGAAGGCGATCACAACCATTCACTGTCCATCACTGATGCGTCCGCAGCAGCTGCCATGGT
CCTCGAATCATCTTAG

>*HmoWRKY7*

ATGGCAGTGGAGCTTATGGCAAGCTCCTCCTCTTACAAGGAGAGCTTCGCAGCTCTAATGGAGGAAAA
CGCGGTCAGAGAAGCCGCCGCGGCCGGCCTTCAGAGCGTCGAAAAGCTCATCAAACTCATCTCTCAGA
TGGACAACCAGCACCACACCACCTCCTCCTCACTCAACAACAACAGCAACGACTCTAGAAACTCCTC
GATCCGATTCGATGATTCCGATTACAAGGCGGTGGCGGACGTCGCTGTCAACAGGTTCAGGAAGGTAA
TCTCTCTCCTCGACCGCACTCGCACCGGCCACGCGCGCTTCCGGCGCGGTCCGCTCTCTTCACCG
CCTCCTCCTCCGCCGCCACTTTCGCTGAAGCCGATCATCCACGGCGGCGCAGGCTCGGCCTTCACGAG
AGTCCACGGTGGTGGAGCTTCTCAGATCCAGCGTCTGCCGCCGCTTCCGCAGCCGAAGACGGTGGCG
GAGAAAAAGGAATCGGCATCGACGACGATCCATTTCTCGACGTCACCGCCGATGTCTGCCGCGAACTC
GTACATCTCGTCGTTGACCGGCGACACCGAGAGTTTGCCGGCGTCGATGTGCTCCGGCGGGTTTCAGA
TTACGAATTTGTCGCAGGTTTCTTCCGCCGGAAAACCGCCTCTCTCGTCTTCGTCGTTGAAGCGGAAGT
GTAACTCCATGGATGACTCCAAGTGTGGCGGTTCTTCTGGACGTTGCCATTGCTCTAAGCGAAGGAAAT
CAAGGACAAAGAGGGTGGTAAGAGTACCAGCAATAAGCTTGAAGATGGCGGATATTCCACCGGATGAT

TACTCCTGGAGGAAGTACGGTCAGAAGCCAATCAAGGGGTCTCCTCATCCAAGGGGATATTACAAGTG
TAGTAGCGTGCGGGGGTGTCCAGCAAGGAAGCATGTGGAGCGTGCATCGGACGATCCATCGATGCTGA
TCGTGACGTACGAAGGCGATCACAACCATTCACTGTCCATCACTGATGCGTCCGCAGCTGCTGCCATGG
TCCTCGAATCATCTTAG

>*HuWRKY8*

ATGCATCTTCTTCTTCTTTATTATGTCATCTTCAAACCTATATATTCCCCACCAACCCCGGCCATCCCCTCT
CTCTCTCTCTCTCTCTCTCTCTCTCGCGTCTCAATCATGGAAGGTTTCTCATCCAAGTTTAGCTCTTCGA
CTAACTCAGGCTCAGCATCATCTTCTGCTACTAATATCTTCCCTTTTGATGGAGAATATTATGATCATCAA
CATCATCATCAAAATGATGGGCAGTTGATTAATGGTATGATGATGACGATGATGATGGACCAGCAAGATG
CGCAAGAGCAGCTGAATCAACGGCAGAGATTGGACTTGGCTCCGGTGGTTCGATCCCAGCCCTTGATC
GATGATAAAAGTAACAATACTAATGGTTACTTATCGTGTAATTACAATCATCATCATCATCATCATCATCAT
CAAAATGATGATGACAAAAGTAATGATGGTGCTGAATTGAAGAAGAAGAAGAAGGGAATAGCAGCATT
AGAGAAGAAGATAAGGAAGCACAGATATGCGTTTCAAACCAGGAGCCAAGTTGATATACTGGATGATG
GCTACAGGTGGCGAAAATATGGCCAAAAAGCCGTCAAAAATAATAAGTTCCCAAGAAGCTACTATCGA
TGCACACATCAAGGATGTAATGTGAAAAAACAAGTTCAACGGCTAACAAAAGATGAAGGTGTTGTCGT
CACCACTTACGAAGGAATTCACTCTCATCCTATTGAAAAGTCTACGGACAACTTTGAGCACATCCTGAC
CCAAATGCAAATCTACACTTCGCTTTAA

>*HmoWRKY8*

ATGCATCTTCTTCTTCTTTATTATGTCATCTTCAAACCTATATATTCCCCACCAACCCCGGCCATCCCCTCT
CTCTCTCTCTCTCTCTCTCTCTCTCGCGTCTCAATCATGGAAGGTTTCTCATCCAAGTTTAGCTCTTCGA
CTAACTCAGGCTCAGCATCATCTTCTGCTACTAATATCTTCCCTTTTGATGGAGAATATTATGATCATCAA
CATCATCATCAAAATGATGGGCAGTTGATTAATGGTATGATGATGACGATGATGATGGACCAGCAACATG
CGCAAGAGCAGCTGAATCAACGGCAGAGATTGGACTTGGCTCCGGTGGTTCGATCCCAGCCCTTGATC
GATGATAAAAGTAACAATACTAATGGTTACTTATCGTGTAATTACAATCATCATCATCATCATCATCATCA
AAATGATGATGACAAAAGTAATGATGGTGCTGAATTGAAGAAGAAGAAGAAGGGAATAGCAGCATTAG
AGAAGAAGATAAGGAAGCACAGATATGCGTTTCAAACCAGGAGCCAAGTTGATATACTGGATGATGGC
TACAGGTGGCGAAAATATGGCCAAAAAGCCGTCAAAAATAATAAGTTCCCAAGAAGCTACTATCGATG
CACACATCAAGGATGTAATGTGAAAAAACAAGTTCAACGGCTAACAAAAGATGAAGGTGTTGTCGTCA
CCACTTACGAAGGAATTCACTCTCATCCTATTGAAAAGTCTACGGACAACTTTGAGCACATCCTGACCC
AAATGCAAATCTACACTTCGCTTTAA

>*HuWRKY9*

ATGGACTCATCTAAGTGGATACTCAATCCCCCTCTCGGGTTCAATCTTAACCTTAGTCTTTCTGATTCGG

ATTCTCTTAGATGCGAATCAAGAAGTCCATCAAATGATGCGATTTTATGTCAACAGGTGACGAACAAAG

AGAGTGAGAACCTAGTGGAGAAGATGGACAGAATGAGAACAGAGAACAAGAAATTGACACAAATGTT

GACATCAATGTGTGAGAATTACAATGTTTTGCAAAGCCAGTTGGTGAATTTGTTGAGTCAAACTCCAAA

AGCTGAAAATAGAGATGATTATCTTAAGAAAAGGAAGGCTTGTTTTGAGGCTATCGATGGTGAAGATAT

TCAGGGGAGTTCAGAGCATAGCTGCATCAGCTACGAAGATTCATGTAAAAGAGCAAGGCTGCTAAGTT

CTAAGCCTAAGGTGTCAAAGATCTTCGTTCGAACTGAAAAATCCGATACGAAACTGGGTGTGAAGGAT

GGATATCAATGGAGAAAATATGGGCAAAAGGTCACAAAAGATAACCCAAGTCCTAGAGCCTACTTTAA

ATGTGCCAATGCCCCTATGTGCCCCGTGAAAAAGAAGGTGCAAAGAAGCATCGAGGACCCATTCATATT

GGTTGCAACATATGAAGGGGAGCACAACCATGCAAAAGATACAGACCAAGTAGTCTTAGGCTCAAGTC

ACTGTTCCATTACTCATGCAAACGCTAGCATGATCCATAATGAGTTGATGATGAACCCTAATTTGCCTAA

CAAGACTATGGATGACTATCAAAATCCGCCAAGCGTGGACTTTGATATCAAAACACTACATCGATCGTT

GGTGGATAAAATGGCTGCTTCATTAACTAGAGATCCTTGTTTCACATCTTCATTAGCAGCAGCGATATCT

GGGAAGATGTTAGATGACAGTCATTCTAAGAGATGGCTTTAA

>*HmoWRKY9*

ATGGACTCATCTAAGTGGATACTCAATCCCCCTCTCGGATTCAATCTTAACCTTAGTCTTTCTGATTTGGA

TTCTCTTAGATGCGAATCAAGAAGTCCATCAAATGATGCGATTTTATGTCAACAGGTGACGAACAAAGA

GAGTGAGAACCTAGTGGAGAAGATGGACAGAATGAGAACAGAGAACAAGAAATTGACACAAATGTTG

ACATCAATGTGTGAGAATTACAATGTTTTGCAAAGCCAGTTGGTGAATTTGTTAAGTCAAACTCCAAAA

GCTGAAAATAGAGATGATTATCTTAAGAAAAGGGAGACTTGTTTTGAGGCTATCGATGGTGAAGATATT

CAGGGGAGTTCAGAGCATAGCTGCATCAGCTACGAAGATTCATGTAAAAGAGCAAGGCTGCTAAGTTC

TAAGCCTAAGGTGTCAAAGATCTTCGTTCGAGCTGAAAAATCCGATACGAAACTGGGTGTGAAGGATG

GATATCAATGGAGAAAATATGGGCAAAAGGTCACAAGAGATAACCCAAGTCCTAGAGCCTACTTTAAAT

GTGCCAATGCCCCTATGTGCCCCGTGAAAAAGAAGGTGCAAAGAAGCATTGAGGACCCATCCATATTG

GTTGCAACATATG

>*HuWRKY10*

ATGACAAGACTCCGACCCCGCGTTGTCAGGGCACGGTGGAACTTATTTAATCGAAAAAAGAAAAAGG

AAAAGCAAAAAGCAAAATCATTTATTGAGTCCCTAAAAAGCGAAACGACGTCGCTCTATGCTCCCGCT

TTTCTCTCTTTCTCTCTCTTTTCTTTCAGATCCGTTGGAACTAGTCAACACTCTCACGGCTCCACTCTCT

CTCTGTCTCTCTCTCCCCGCCTTTCTCTCTCCTCTGTCCATTCCCAAACTACCCTTCTCTCTCTTCGCTAT

CCATCCTCAGCGTCAGCGTCACAGGTGAATTGCCCGGCGAATGCGGTGATGGAAGCCGCTCCGTCCGT

CGCTCCGTCACCGTCTGGGTTGATGATGGCCACCGTAGACGGCGGCGATTTGAGGAGAGCTGCCACCA

ACGACGGCGGAGGCGGATCTAGTAATGCCAAGTACAAGCTGATGTCCCCGGCGAAGCTTCCGATCTCG

AGGTCTCCTTGCCTTACCATTCCTCCTGGACTCAGTCCGACGTCGTTTCTTGAGTCCCCCGTCCTTCTCT

CCAACTTCAAGGGAGAGCCTTCCCCAACAACTGGTTCTTTTTCCATCCTCAGATGATGAACTTCTCTT

CTGGTGGCTCTACGGCATTCCCTTCTATCATAAATAGCTCCGGTAGCCTGGATGATATTAAATCGAGTGG

CTTTGAGTTCAAACCTGGTCCAGGATCATATTCCACTGCAAACTTTTCTATGGCAGGGCATGTGGTTCCT

TCTGCAGATATGAATCAACAGCAAGTTGCACAAGCTGCACAAGTCCAATCGGAGTTCCAGTTGCAAGC

ATCTGACACATCCATAACTGTTAAAGACGAAGTTCCGGTCTCAAATGAGCTCCAGTCTGTAGTGGTTGC

TTCAGCTTCTCAGGCATCACCTGATATTGATTCTAATGAAATAAACCAAGGAGAAGACTCCAATAATGA

AGCCCAGGTGCCACAATCTGAGCAGAAAGTAGGAGGATCTCATGTCATAACTGATCGAACATCTGATGA

TGGATATAATTGGAGGAAATATGGACAGAAGCTTGTCAAGGGAAGTGAATACCCACGAAGTTATTACAA

ATGTACACATCCGAATTGTGAGGTCAAAAAACTGTTTGAGAGGTCACCTGATGGACAGATAACAGAGA

TCATCTACAAGGGTACACATGATCACCCAAAACCTCAACCGAATCGTCGACTTGCTGCTGGTTCTCTGG

TGTCAGCGCACGACGAGAAGTCAGAGAGAGTTCCTGTGCTTACTGATCAAGAAGCCAAGCCTGCAAA

CTTGTTTGGCCAATCAGCCGGCAATGACGAGAGCAGCACTCCTGATATACTCCCTGGTGGAGCAAATG

ACAATAATGGAGAAGGTGGTCTTCTAAATAGAAGCACTGATGATGCTGATGATGATGATCCATTTGCAA

AGAGGAGGTATTGGATATCTTCAGCATTTCCGTTAAATATATGTAACCATAGGCAGGGCAATGAACTTAT

ATTCCTTGTTATCAGGAGGATGGAAGGCGGTGAAATGGACTTCAATTCGGTAGTGAAACCTATTCGAGA

ACCACGTGTTGTTGTTCAAACTGTGAGTGAGGTTGATATTCTTGATGATGGGTACCGATGGCGTAAGTA

CGGCCAGAAGGTGGTGAGAGGCAACCCTAATCCAAGGAGTTATTACAAGTGCACTCATCCAGGATGCC

CAGTTCGTAAACATGTAGAGAGGGCATCTCATGACCCTAAAGCAGTGATAACAACATACGAGGGGAAA

CACAATCATGAAGTACCGACTGCAAGGAATAGCAGCCATGACACAGTGGCTACAGCAACCGCGAGTCT

GGCAACCCGAATTGGGTCACATGAAGGTAGTGAACAAGTAAGCCTCGATCTTGGGGTCGGGATTAGGT

CTGCTCCTGAATTTGTACAGGGTCAGCAGCTGGCAATGGAACCTGAGCGCATTCCCGGCCAAACCCAT

TTCAGCAGTCCAAATCTCTTATTGCTTCAACAACCTCCTCTTTCAACTTTTCCTAGTTATGTGAATGGTG

GTATGGTGCTTTATGGTTCTAGGGAAAACCATAGTCAAGCTCATAGTGTTGGTAATGCACCTGTAAATCA

CTCTTCTAATTCGTATCAGCAGAACCTGGGAAGAATACTCATGGGTCCATAG

>*HuWRKY11*

ATGGCTATAGATCTCATGGTAGGCTACAAAAACGACGCCGCTGCTTCCCTTTCTCTCTCCTCTCAAATGG
AGGATACCCAAGCCCAAACCATCGCCCACGAAGCTTCCAAAGGCCTCCAGAGCGTCCACAATTTCATC
AACCTTCTCTCCAAATCTACTGACAATTCTCAACCTGCCAAGTCAGCTGGCACCATGGACATCGACCTT
AACTGCAGGGCCGTCGCCGACGACGCCGTTTCAAGATTCAAGAACGTCATCTCTCTCCTCGGCCGGGC
TAGGACGGGCCACGCCAGGTTCCGCCGGGCCCCCGTTTCACCGCCCCTCTCTCCTCCGAAGCGGGGCG
GCGGCGACGTCGTGGTGTTGTGTCCTAAGCCAATCCAGCAGGTTCCGCCGGTTCAGACCGGCTTTGAT
TTTAAACCGGCGGCGGTGATAAATTTCGCGTACTCGTCGCCGGTTGTCTCCTCCGCCGGGAACTCGGTG
AAGTCAACGTTGACCGGCGAGACGGATAGCAAGCAGAACACAACGGCTACTACTACAACAACGACGT
CGTTTCAGATAAGCCATTATTCGGGGATGTCCTCGGCTGGGAAGCCGCCTCTGTCTCATACGACGTCGT
TTAAGGTGAGGAAGTGCCACTCTGAGAACGGCAAGTGTGGTGGATCTTCTGGTCGTTGCCACTGCTCT
AAGAAAAGGAAGTTGAGGTTGAAGAGGGTGGAGAGAGTACCGGCAATAAGTTCAAAGATGGCAGAC
ATACCACCGGATGACTATTCCTGGAGAAAATACGGTCAAAAGCCCATCAAAGGCTCCCCACATCCAAG
AGGGTACTACAAATGCAGTACGGTTAGAGGGTGCCCAGCTCGGAAACACGTAGAGAGGGCGTTGGAT
GATGCGAACATGTTGATCGTCACCTATGAAGGGGAGCACAACCATGCTCTGTCTATTGCTGAAGCGTCT
ATTCTCGAGTCATCTTAA

>*HmoWRKY11*

ATGGCTATAGATCTCATGGTAGGCTACAAAAACGACGCCGCTGCTTCCCTTTCTCTCTCCTCTCAAAT
GGAGGATACCCAAGCCCAAACCATCGCCCACGAAGCTTCCAAAGGCCTCCAGAGCGTCCACAATTTCA
TCAACCTTCTCTCCAAATCTGCTGACAATTCTCAGCCTGCCAAGTCAGCTGCCACCATGGACATCGACC
TTAACTGCAGGGCCGTCGCCGACGACGCCGTTTCAAGATTCAAGAACGTCATCTCTCTCCTCGGCCGG
GCCAGGACGGGCCACGCCAGGTTCCGCCGGGCCCCCGTTTCATCCCCCCACTCTCCTCCGAAGCGGGG
CGGCGGCGACGTCGTGGTGTTGTGTCCGAAGCCAATCCAGCAGGTTCCGCCGGTTCAGACCGGCTTTG
ATTTTAAGCCGGCGGCGGTGATAAATTTCGCGTACTCGTCGCCGGTGCTCTCCTCCGCCGGGAACTCGG
TGAAGTCAACGTTGACCGGCGAGACGGATAGCAAGCAGAACACAACAACGACAACGGCTACTACTAC
AACAACGGCGTCGTTTCAGATAAGCCATTATTCGGGGATGTCCGCCGCTGGGAAGCCGCCTCTGTCTCA
TACGACGTCGTTTAAGGTGAGGAAGTGCCACTCTGAGAACGGCAAGTGTGGTGGATCTTCTGGTCGTT
GCCACTGCTCTAAGAAAAGGAAGTTGAGGTTGAAGAGGGTGGAGAGAGTCCCGGCAATAAGCTCAAA
GATGGCAGACATACCACCGGATGATTATTCCTGGAGAAAATACGGTCAAAAGCCCATCAAAGGCTCCC
CACATCCAAGAGGATACTACAAATGCAGTACGGTTAGAGGGTGCCCAGCTCGGAAACACGTAGAGAG

GGCGTTGGATGATGCAAACATGTTGATCGTCACCTATGAAGGGGAGCACAACCATGCTCTGTCTATTGC
TGAAGCGTCTATTCTCGAGTCATCTTAA

>*HuWRKY12*

ATGTTGAACCAGGAGAACTTGATTGATCAAGACGATGATCAAGGGATGATGATGATGTCTTCCCAAATG
GGTTTCTCTCAAAACCCTATGATCAACTCAACTAATAATCCGACAACCACAAGCTTTGCCCCTCTTGTTT
CTTCTTTTGCATTAGATCATGCAACTTTTAGTACTCTCACTCAAGGCCTCCTTTCTTCCATCTCTCCAGTT
CCAAAGCCACCGTCTAGCATGCTTACTCTACCTTTTTCTCCCCAAAAATCAACCACAAATCCATGGGCA
TGGGAAGAAAAGGAGGAGAGCCAAGGGGTGAATAGGAGAAGCATGACCAATGGACTGGAGCAACCT
CCCACGGTATCCGCGATGAAGATGAAGAATAAGATCAAAGGGAGAAGGAAGGTTAGAGAGCCAAGAT
TTTGCTTCAAGACCTTGAGTGATGTGGATGTCTTGGATGATGGTTACAAATGGAGGAAGTATGGCCAAA
AAGTAGTCAAGAACACCCTTCACCCAAGGAGTTACTACAGATGCACGCAGGACAACTGCAGGGTGAA
GAAGAGAGTGGAGAGATTAGCAGACGATCCGAGGATGGTGATCACCACCTATGAAGGAAGACATGCTC
ATTCTCCCTCCCATGACCTTGAGCAAGATTCACAGCAGACCCCTCATCCCTTTCTCAATAACTTCTTTTG
GTAG

>*HmoWRKY12*

ATGTTGAACCAGGAGAACTTGATTGATCAAGACGATGATCAAGGGATGATGATGATGTCTTCCCAAATG
GGTTTCTCTCAAAACCCTATGATCAACTCAACTAATAATCCGACAACCACAAGCTTTGCCCCTCTTGTTT
CTTCTTTTGCATTAGATCATGCAACTTTTAGTACTCTCACTCAAGGCCTCCTTTCTTCCATCTCTCCAGTT
CCAAAGCCACCGTCTAGCATGCTTACTCTACCTTTTTCTCCCCAAAAATCAACCACAAATCCATGGGCA
TGGGAAGAAAAGGAGGAGAGCCAAGGGGTGAATAGGAGAAGCATGACCAATGGACTGGAGCAACCT
CCCACGGTATCCGCGATGAAGATGAAGAATAAGATCAAAGGGAGAAGGAAGGTTAGAGAGCCAAGAT
TTTGCTTCAAGACCTTGAGTGATGTGGATGTCTTGGATGATGGTTACAAATGGAGGAAGTATGGCCAAA
AAGTAGTCAAGAACACCCTTCACCCAAGGAGTTACTACAGATGCACGCAGGACAACTGCAGGGTGAA
GAAGAGAGTGGAGAGATTAGCAGACGATCCGAGGATGGTGATCACCACCTATGAAGGAAGACATGCTC
ATTCTCCCTCCCATGACCTTGAGCAAGATTCACAGCAGACCCCTCATCCCTTTCTCAATAACTTCTTTTG
GTAG

>*HuWRKY13*

ATGTTGAACAGCCAAGGTTTACTCTTCGAGGATAGTCAAGCTGGGATCATGTCCACCCAAATGGGGTTT
TGCCTAAATCCTACGAACTTGACTTCCAGCCTTGTTGCTCCTCTTGCTTCTCATCAAGACCAACAATATG
TTAAGCCCTTCATCAATAATATCATACCTTCTTTGTTTGGAATGGATCAGGTTTCCTCCACTCTCACTCAA

GCGCTTTTGTCTTCTACCTCTCCAAAGCCTAGAGAAGACCTCATTTCCTCATCTTATAACATGCCTCAAC
TGGTCTCTCCTCAAAGATTTACTCCGAATATGTGGGCATGGGGAGGGAACAAGGAGGGCCAAGGAATG
GAGAAGAGACCAAATGGGGAGAATCCTCAATCTCTAGGGGTATCGATGAGGAAGATGAAAAGGGTAA
AAGGGAGAAGGAAGGTGAGAGAGCCAAGATTTTGCTTCCAAACCATGAGCGATGTGGATGTCTTGGAT
GATGGATACAAGTGGAGGAAGTATGGCCAAAAAGTTGTGAAGAACACCCTTCATCCAAGGAGCTATTA
TCGTTGCACACAAGACAACTGCAGGGTGAAGAAGAGAGTGGAGAGACTGGCAGAGGATCCGAGGAT
GGTGATCACCACCTATGAAGGACGCCACATCCATTCTCCCTCACACGACCAGCTCGACGAGTCACAAC
CTCAACCTCTTCTTAGTAACTTCTTCTGTTAG

>HmoWRKY13

ATGTTGAACAGCCAAGGTTTACTCTTCGAGGATAGTCAAGCTGGGATCATGTCCACCCAAATGGGGTTT
TGCCTAAATCCTACGAACTTGACTTCCAGCCTTGTTGCTCCTCTTGCTTCTCATCAAGACCAACAATATG
TTAAGCCCTTCATCAATAATATCATACCTTCTTTGTTTGGAATGGATCAGGTTTCCTCCACTCTCACTCAA
GCGCTTTTGTCTTCTACCTCTCCAAAGCCTAGAGAAGACCTCATTTCCTCATCTTATAACATGCCTCAAC
TGGTCTCTCCTCAAAGATTTACTCCGAATATGTGGGCATGGGGAGGGCAAAAGGAGGGCCAAGGAATG
GAGAAGAGACCAAATGGGGAGAATCCTCAATCTCTAGGGGTATCGATGAGGAAGATGAAAAGGGTAA
AAGGGAGAAGGAAGGTGAGAGAGCCAAGATTTTGCTTCCAAACCATGAGCGATGTGGATGTCTTGGAT
GATGGATACAAGTGGAGGAAGTATGGCCAAAAAGTTGTGAAGAACACCCTTCATCCAAGGAGCTATTA
TCGTTGCACACAAGACAACTGCAGGGTGAAGAAGAGAGTGGAGAGACTGGCAGAGGATCCGAGGAT
GGTGATCACCACCTATGAAGGACGCCACATCCATTCTCCCTCACACGACCAGCTCGACGAGTCACAAC
CTCAACCTCTTCTTAGTAACTTCTTCTGTTAG

>HuWRKY14

ATGGACAATTATCAAGGGGATCTAGCAGACATGGTCCGAGCCAGCAGTGGTGCGATCAGAACCATCAC
CAGCGTCGGTACTACTATGACTGCTACGACTGCTACTGCTGGGACTACTACAACAACTGATGAACAACT
GCCTGAGTCAGAGTGGCAATTCAGATCCTCGAATCCAGATCTATGCCCGTTCCCTGATCTGCTCCTCAA
CAACGTGGATCCAATAACGAGAACAGATTCGCTGTCATCTTTCATCAATGTAGGGTTTGAAGATCGTCA
AAACACGCCATATGAAGGTGTTGGTGGTGGTGGGTTGATGACAAACAACTTCAACGTGTTCGCAAAGG
TCCTCCAGATCTCATCAAATATGTATAGTTCTACTGATGGCAATAACAATAACGGTATCAACAGCAGCAG
CTACAACAACAACAATAATTCAAAGACGCCATCAATAGCAAGAGGCTCATCTAGCTTTATGAAAACAGA
TTGTGGATTGATGTCAAATGAGAAGATCATCAACAATAACGTAAGTAGCAGCAGCAATACAAAGGATG
AGTTCCTTGACAATTGCACCTCCGGAACTCAGATCACTTCACTAGAGAGTCCTGGCAATAAAAGAAGG

AAGAGCCAAGCCAAGAAAGTAGTGTGTATACCAGCACCAACAGGAGCACATAGCAGGCAAAGTGGAG
AAGCAGTTCCATCTGATCTCTGGGCTTGGAGGAAATATGGTCAGAAACCTATTAAAGATTCTCCTTATCC
CAGAGGCTACTATAGATGTAGTAGCTCTAAGGGGTGTTCAGCAAGAAAGCAAGTGGAAAGGAGTCGA
ACGGACCCGAACATGTTGGTTATTACATACACCTCGGAGCACAATCACCCTTGGCCAACTCAGAGGAA
CGCCCTTGCTGGATCAACGCGCTCTCAAACATCCAAGAATATTGCTACTGCACTCACCTCTTCGAAGCT
CAACAAAGCACTCGTCCAGGAGGAAGTCAGCACTAACAAGGAGATTAGCCAGGCGAAAAACTTTGTC
GAAGAAGAGATGAAGATTGAGAAGCAAGTAGAGGAAAAAGGGGAAGATCTTAGTTTGACGGTGAGTT
ATAAGCCCACATTGCATGAACTGAATGGTAAGCAGCCTCAACAGGACTTGTCTATGGAGTTGGGTTTCG
ATGGCGACGACCCTCTTGATCTCATATTCACCCAAGAAATCAAATCATTGGATCCCTTTAATGGTCTCTT
TGATTGGGATGCACCACAAAGTGCCATGGTCCAAGGTAATGGCAACCAAGAAAAAAGGGTTTCTTGA

>*HmoWRKY14*

ATGGACAATTATCAAGGGGATCTAGCAGACATGGTCCGAGCCAGCAGTGGTGCGATCAGAACCATCAC
CAGCGTCGGTACTACTATGACTGCTACGACTGCTACTGCTGGGACTACTACAACAACTGATGAACAACT
GCCTGAGTCAGAGTGGCAATTCAGATCCTCGAATCCAGATCTATGCCCGTTCCCTGATCTGCTCCTCAA
CAACGTGGATCCAATAACGAGAACAGATTCGCTGTCATCTTTCATCAATGTAGGGTTTGAAGATCGTCA
AAACACGCCATATGAAGGTGTTGGTGGTGGTGGGTTGATGACAAACAACTTCAACGTGTTCGCAAAGG
TCCTCCAGATCTCATCAAATATGTATAGTTCTACTGATGGCAATAACAATAACGGTATCAACAGCAGCAG
CTACAACAACAACAATAATTCAAAGACGCCATCAATAGCAAGAGGCTCATCTAGCTTTATGAAAACAGA
TTGTGGATTGATGTCAAATGAGAAGATCATCAACAATAACGTAAGTAGCAGCAGCAATACAAAGGATG
AGTTCCTTGACAATTGCACCTCCGGAACTCAGATCACTTCACTAGAGAGTCCTGGCAATAAAAGAAGG
AAGAGCCAAGCCAAGAAAGTAGTGTGTATACCAGCACCAACAGGAGCACATAGCAGGCAAAGTGGAG
AAGCAGTTCCATCTGATCTCTGGGCTTGGAGGAAATATGGTCAGAAACCTATTAAAGATTCTCCTTATCC
CAGAGGCTACTATAGATGTAGTAGCTCTAAGGGGTGTTCAGCAAGAAAGCAAGTGGAAAGGAGTCGA
ACGGACCCGAACATGTTGGTTATTACATACACCTCGGAGCACAATCACCCTTGGCCAACTCAGAGGAA
CGCCCTTGCTGGATCAACGCGCTCTCAAACATCCAAGAATATTGCTACTGCACTCACCTCTTCGAAGCT
CAACAAAGCACTCGTCCAGGAGGAAGTCAGCACTAACAAGGAGATTAGCCAGGCGAAAAACTTTGTC
GAAGAAGAGATGAAGATTGAGAAGCAAGTAGAGGAAAAAGGGGAAGATCTTAGTTTGACGGTGAGTT
ATAAGCCCACATTGCATGAACTGAATGGTAAGCAGCCTCAACAGGACTTGTCTATGGAGTTGGGTTTCG
ATGGCGACGACCCTCTTGATCTCATATTCACCCAAGAAATCAAATCATTGGATCCCTTTAATGGTCTCTT
TGATTGGGATGCACCACAAAGTGCCATGGTCCAAGGTAATGGCAACCAAGAAAAAAGGGTTTCTTGA

>*HuWRKY15*

ATGGCTGTAGATCTCATGTTGGGTTACAGCAACTCCTCCTCCTCTAATAACCGTTCTCTCTCCTCTAAAA

TGGAGGAAGCCATCGCTCAAGAAACCGCCGCTGGTCTTCAAAGCGTCGAGAATTTCGTCAACCTCCTA

TCCAAACAGTCGCACCATCAACCTAAACCAGAGGCCATGGAAATCGATCTCAACTGCCGGGCTGTGGC

GGACGACGCCGTTTCCAGGTTCAAGAAGGTCATCTCTCTCCTGGGCCGAGCTCGGACGGGCCACGCCC

GCTTCCGTCGGGGCCCGGTTTCGCCACGCCAGCAGCCGCAGAACGTCGTCGTTCTGTGCCCCAAGCCT

ATTCAGCAAGTGCCCCTTGTGCATGATTTTTCGACGTTGGTGATGCGAGATAGGAAGAAGGAGGTGGC

CGTGCCACCGACGACGGCCACGATTAATTTCTCCTACTCGGCGGGTACCTCGATGAAGTCAACGTTGAC

CCGGGAGAATGAGAGCAGTAAACAACAGGCGGAGACTACTACGACTACTACAACCACTACGACGTCG

TTTCAGTTAACGAATTATTCCGGAGTGTCATCGGCGGGGAAGCCGCCTCTTTGTCATACGACGTCGTTT

AAGGTTAGGAAGTGTTACTCTGAGAACGGCAAGTGTGGTGGTGGGTCCTCCGGTCGTTGTCACTGCTC

CAAGAAGAGAAAATTGAGGTTGAAGAGGGTGGTGAGAGTGCCGGCTATAAGCTCAAAAATGGCGGAT

ATACCCCCAGATGATTATTCTTGGAGAAAATATGGGCAAAAGCCCATCAAAGGCTCCCCTCATCCAAGA

GGATACTACAAGTGCAGTACAGTTAGAGGATGCCCAGCTCGAAAGCATGTAGAGAGGGCATTGGACGA

TCCAAACATGTTAGTTGTCACATATGAAGGCGAGCACAACCATTCTCTCTCCGTCGTTGAAGCCTCCAG

TCTGATCCTTGAGTCATCTTAA

>*HmoWRKY15*

ATGGCTGTAGATCTCATGTTGGGTTACAGCAACTCCTCCTCCTCTAATAACCGTTCTCTCTCCTCTAAAA

TGGAGGAAGCCATCGCTCAAGAAACCGCCGCTGGTCTTCAAAGCGTCGAGAATTTCGTCAACCTCCTC

TCCAAACAGTCGCACCATCAACCTAAACCAGAGGCCATGGAAATCGATCTCAACTGCCGGGCTGTGGC

GGACGACGCCGTTTCCAGGTTCAAGAAGGTCATCTCTCTCCTGGGCCGAGCTCGGACGGGCCACGCCC

GCTTCCGTCGGGGCCCGGTTTCGCCACGCCAGCAGCCGCAGAACGTCGTCGTTCTGTGCCCCAAGCCT

ATTCAGCAAGTGCCCCTTGCGCATGATTTTTCGACGTTGGTGATGCGAGATAGGAAGAAGGAGGTGGC

CGTGCCACCGACGACGGCCACGATTAATTTCTCCTACTCGGCGGGTACCTCGATGAAGTCAACGTTGAC

CCGGGAGAATGAGAGCAGTAAACAACAGGCGGAGACTACTACGACTACTACAACCACTACGACGTCG

TTTCAGTTAACGAATTATTCCGGAGTGTCATCGGCGGGGAAGCCGCCTCTTTGTCATACGACGTCGTTT

AAGGTTAGGAAGTGTTACTCTGAGAACGGCAAGTGTGGTGGTGGGTCCTCCGGTCGTTGCCACTGCTC

CAAGAAGAGAAAATTGAGGTTGAAGAGGGTGGTGAGAGTGCCGGCTATAAGCTCAAAAATGGCGGAT

ATACCCCCAGATGATTATTCTTGGAGAAAATATGGACAAAAGCCCATCAAAGGCTCCCCTCATCCAAGA

GGATACTACAAGTGCAGTACAGTTAGAGGATGCCCAGCTCGAAAGCATGTAGAGAGGGCATTGGACGA

TCCAAACATGTTAGTTGTCACATATGAAGGCGAGCACAACCATTCTCTCTCCGTCGTTGAAGCCTCCAG

TCTGATCCTTGAGTCATCTTAA

>*HuWRKY16*

ATGGATGGGTATGAATTTGGTAAATCAGGAGGTGACTGGGATTTGCAGGCCATTGTCAAAGGGTGCAA

CAGTCCTGTGATCAGTCAATCATGGATTCAATCTCTAAGCCAGGAAGAAGATCACAGCACATGCCATGA

AAACAAAGCTTTGCAAGGCGCGACAAGCGTCCATGACTCCGCGAAAAGTCCAACTCGAGTGCAAAAG

CAGAAGGCAGTGGGTGTTTCTTGTGCAAATTCTCATGGAGTTACCAAAGAAAGAAGGAGGAAGCCCA

AACAGAGGACCACCTTTGTTCAGCAAGTTGCAGGGGATCGCGCCTTGTCAGATAACTGGAACTGGAG

AAAATATGGCCAGAAAACCATAAAGGGTTCACCCTATCCAAGGAGCTATTACAAATGTAGCAGCTCCGG

AGATTGCTTGGCAAGAAAACAAGTGGAGCACAGCAGCAGTGATCCAGGGACATTCTTGATCACCTACA

TATATGAACATAACCATCCTTATCCAACTCGACGACACTCTCAAGCTGGCCGTACTAAGGACAGATTTGA

TTCATCATCCAAAGTCCACGCAACTAGTGAATCATCAACAAACAAGCGAAGTACGACCAACACAATTA

GCTCCCTCGAATCCCCATTATTTGGAGAGAATGATCGCATTTCGACTTTGAAATGCGAAAAAGATGAAG

GAAACTCAGAAGATGCCGGAGGAGGAGGAGGAGATGTTGATGTTTCAAACATGATGTGGGATGATGAA

TTTTTTGCAGGGTTAGAGCACTTGGAGGGATTAGAAATGGATCTATTATGA

>*HmoWRKY16*

ATGGATGGGTATGAATTTGGTAAATCAGGAGGTGACTGGGATTTGCAGGCCATTGTCGAAGGGTGCAA

CAGTCCTGTGATCAGTCAACCATGGATTCAATCTCTAAGCCAGGAAGAAGATCACAACACATGCCATG

AAAACAAAGCTTTGCAAGGCGCGACAAGCATCCATGACTCCGCGAAAAGTCCTACTCGAGTGCAAAA

GCAGAAGGCAGTGGGTGTTTCTTGTGCAAATTCTCAGCGAGTTACCAAAGAAAGGAGGAGGAAGCCC

AAACAGAGGACCACCTTTGTTCAGCAAGTTGCAGGGGATCGCGCCTTGTCAGATAACTGGAACTGGA

GAAAATATGGCCAGAAAACCATAAAGGGTTCACCCTATCCAAGGAGCTATTACAAATGTAGCAGCTCCG

GAGATTGCTTGGCAAGAAAACAAGTGGAGCACAGCAGCAGTGATCCAGGGACATTCTTGATCACCTAC

ATATATGAACATAACCATCCTTATCCAACTCGACGACATTCTCAAGCTGGCCGTACTAAGGACAGATTTG

ATTCATCATCCAAAGTCCACGCAACTAGTGAATCATCAACAAACAAGCGAAGTACGACCAACACAATT

AGCTCCCTCGAATCCCCATTATTTGGAGAGAATGATCGCATTTCGACTTTGAAATGCGAAAAAGATGAA

GGAAACTCAGAAGATGTCGGAGGAGGAGGAGGAGATGTTGATGTTTCAAACATGATGTGGGATGATGA

ATTTTTTGCAGGGTTAGAGCACTTGGAGGGATTAGAAATGGATCTATTATGA

>*HuWRKY17*

ATGGCTCTTGAAGAAAAACTAGCCAATGAAGCGGTTCAAAAAGGATTAACATATGCACATAAGTTCATC

AGCTCCATTTCTGATCAAACCCAACCCAGCATTAGCCTCGATTTCGAAGAATCAAGCCTAATTGCCTATG
AAGCGATCAAGGAGTTCAAAAAATTTGTTTCCCTTCTCGAGGAATCGATGATAAAGAAGCCCAACAGA
GTCAAACAGGGTCCTCTGGCTAAAGTTAGAGGGATAAACCCTAAGGAGATGGTTGATCCTCTGGCTCC
CAGATTGAACCAGTTGGTGTCTTCTCAGGTTCCTGCAACCAAGGAGCTGAGTTTTGTTAAGGGGGGAA
GTAATGTCATAAATATGTCATTTTCAAACCCGGTTTTGTCCTTGGAAGGATCAACCACCAGCAGCAAGC
AAATGGTTATGCAGAGCTCATTTTCCGGGGTTTTGACATTCAGTGGAGATACTAGCACCAGGTGTGCTT
CCTCTAGCAGGACCTGTCATTGCTCCAGGAAAAGGAAACGGAGAGAAAAGCAAAGAATAAAAGTCCC
AGCCATAAGTGAGAAAATAGCAGACATACCACCTGATGATCATTGCTGGAGGAAATACGGCCAGAAAC
CGATCAAAGGTTCTCCACACCCCAGGAGCTACTACAAATGCAGCACTTGCAAGGGGTGCCCAGCAAG
GAAACAAGTGGAAAGATGTTTGGAAGATTCAAGCATGTTGGTTGTGACATATGAAGGAGAACACAATC
ACTCTTCATTTACATTTCCATATCCAGCCATTACATTTTGA

>*HmoWRKY17*

ATGGCTCTTGAAGAAAAACTAGCCAATGAAGCGGTTCAAAAAGGATTAACATATGCACATAAGTTCATC
AGCTCCATTTCTGATCAAACCCAACCCAGCATTAGCCTCGATTTCGAAGAATCAAGCCTAATTGCCTATG
AAGCGATCAAGGAGTTCAAAAAATTTGTTTCCCTTCTCGAGGAATCGATGATGAAGAAGCCCAACAGA
GTCAAACAGGGTCCTCTGGCTAAAGTTAGAGGGATAAACCCTAAGGAGATGGTTGATCCTCTGGCTCC
CAGATTGAACCAGTTGGTGTCCTCTCAGGTTCCTGCAACCAAGGAGTTGAGTTTTGTTAAGGGGGGAA
GTAATGTCATAAATATGTCATTTTCGAACCCGGTTTTGTCCTTGGAAGGATCAACCACCAGCAGCAAGC
AAATGGTTATGCAGAGTTCATTTTCCGGGGTTTTGACATTCAGTGGAGATACTAGCACCAGGTGTGCTT
CCTCTAGCAGGACCTGTCATTGCTCCAGGAAAAGGAAACGGAGAGAAAAGCAAAGAATAAAAGTCCC
AGCCATAAGTGAGAAAATAGCAGACATACCACCTGATGATCATTGCTGGAGGAAATACGGCCAGAAAC
CGATCAAAGGTTCTCCACACCCCAGGAGCTACTACAAATGCAGCACTTGCAAGGGGTGCCCAGCAAG
GAAACAAGTGGAAAGATGTTTGGAAGATTCAAGCATGTTGGTTGTGACATATGAAGGAGAACACAATC
ACTCTTCATTTACATTTCCATATCCAGCCATTACATTTTGA

>*HuWRKY18*

ATGGACTCATCTAAGTGGATACTCAATCCCCCTCTCGGGTTCAATCTTAACCTTAGTCTTTCTGATTCGG
ATTCTCTTAGATGCGAATCAATAAGTCCATCAAATGATGCGATTTTATGTCAACAGGTGACGAACAAAG
AGAGTGAGAACCTAGTGGAGAAGATGGACAGAATGAGAACAGAGAACAAGAAATTGACACAAATGTT
GACATCAATGTGTGAGAATTACAATGTTTTGCAAAGCCAGTTGGTGAATTTGTTAAGTCAAACTCCAAA
AGCTGAAAATAGAGATGATTATCTTAAGAAAAGGAAGACTTGTTTTGAGGCTATCGATGGTGAAGATAT

TCAGGGGAGTTCAGAGCATAGCTGCATCAGCTACGAAGATTCTTGTAAAAGAGCAAGGCTGCTAAGTT

CTAAGCCTAAGGTGTCAAAGATCTTCGTTCGAACTGAAAAATCCGATACGAAACTGGGTGTGAAGGAT

GGATATCAATGGAGAAAATATGGGCAAAAGGTCACAAGAGATAACCCAAGTCCTAGAGCCTACTTTAA

ATGTGCCAATGCCCCTATGTGCCCCGTGAAAAAGAAGGTGCAAAGAAGCATTGAGGACCCATCCATATT

GGTTGCAACATATGAAGGGGAGCACAACCATGCAAAACCTAGTTCAAAAGATACAGACCAGGTAGTCT

TAGGCTCAAGTCACTGTTCCATCACTCATCCAAACGCTAGCATGATCCATGATGAGTTGATGATGAACC

CTAATTTTCCTAACAAGACTATGGATGACTATCAAAATCCGCCAAGCATGGACTTTGATATCAAAACACT

ACATCGATCGTTGGTGGAGAAAATGGCTGCTTCATTAACTAGAGATCCTTGTTTCACATCTTCATTAGCA

GCAGCGATATCTGGGAAGATGTTAGATGACAGTCATTCTAAGAGATGGCTTTAA

>*HmoWRKY18*

ATGGACTCATCTAAGTGGATACTCAATCCCCCTCTCGGGTTCAATCTTAACCTTAGTCTTTCTGATTCGG

ATTCTCTTAGATGCGAATCAATAAGTCCATCAAATGATGCGATTTTATGTCAACAGGTGACGAACAAAG

AGAGTGAGAACCTAGTGGAGAAGATGGACAGAATGAGAACAGAGAACAAGAAATTGACACAAATGTT

GACATCAATGTGTGAGAATTACAATGTTTTGCAAAGCCAGTTGGTGAATTTGTTAAGTCAAACTCCAAA

AGCTGAAAATAGAGATGATTATCTTAAGAAAAGGAAGACTTGTTTTGAGGCTATCGATGGTGAAGATAT

TCAGGGGAGTTCAGAGCATAGCTGCATCAGCTACGAAGATTCTTGTAAAAGAGCAAGGCTGCTAAGTT

CTAAGCCTAAGGTGTCAAAGATCTTCGTTCGAACTGAAAAATCCGATACGAAACTGGGTGTGAAGGAT

GGATATCAATGGAGAAAATATGGGCAAAAGGTCACAAGAGATAACCCAAGTCCTAGAGCCTACTTTAA

ATGTGCCAATGCCCCTATGTGCCCCGTGAAAAAGAAGGTGCAAAGAAGCATTGAGGACCCATCCATATT

GGTTGCAACATATGAAGGGGAGCACAACCATGCAAAACCTAGTTCAAAAGATACAGACCAGGTAGTCT

TAGGCTCAAGTCACTGTTCCATCACTCATCCAAACGCTAGCATGATCCATGATGAGTTGATGATGAACC

CTAATTTTCCTAACAAGACTATGGATGACTATCAAAATCCGCCAAGCATGGACTTTGATATCAAAACACT

ACATCGATCGTTGGTGGAGAAAATGGCTGCTTCATTAACTAGAGATCCTTGTTTCACATCTTCATTAGCA

GCAGCGATATCTGGGAAGATGTTAGATGACAGTCATTCTAAGAGATGGCTTTAA

>*HuWRKY20*

ATGGACGGAGCTCCGGTAGCCTCCGGCAACACGCCAACATCATCCGGCGCCGGCGTTATGGACGGCTC

TGATCTGAGGAGCAGGGGGGCCCACAACGATGCCGGATCATCAACCATTAACGCCAAGTACAAGCTCA

TGTCGCCGGCCAAACTCCCGATCTCTCGTTCGCCTTGCATCACTATCCCTCCTGGCCTCAGTCCGACGT

CGTTTCTTGAATCCCCCGTCCTCCTTTCCAATTTCAAGGGAGAACCTTCGCCAACAACTGGTTCCTTTTT

CAAGCCTCAAATAATGCACAGCTCTTCTTGTGGGTCCACATTCCCTTCTATTATAAATAACTCTGATACCA

TGGATGGTAGTAATTCAAGCGGCTTTGAGTTCAAACCCGATTCTGGATCATTTTCTGCTGGAGGCTTTTC

TTTTGCAAGATCTCTGCTCCCATCTTCAGATTTGAACCGACAACAAGCTATGCAAGTCCAAGCAGAGTG

CCAGTCTCAATCATCTGATCCATCGACAACTGTTAAAAGCGAAGTAGGAGGCTCAACTGAATTGAGATC

TACTATGTTTACTCCAGGATCTGCTATACAGCCTGAAGTTGATTCCAATGAATTAAATCAAACAGCAGAC

TCCAATAATGAAGCCCAGGCACCACAAACTGAGCAGAAAGGAGTAGGGTCTCAAGTCATAACATCAGA

TGATGGGTACAATTGGAGGAAGTATGGACAGAAACTTGTCAAGGGAAGTGAATATCCACGAAGTTATT

ACAAATGTACGCATCCCAACTGTGAAGTCAAAAAGTTATTTGAGCGATCACCTGATGGACATATAACAG

AGATCATCTACAAGGGTACACATGATCATCCGAAGCCTCAACCTAATCGTCGACTCCCAACTGGCTCTA

TGATGTCCACCCTTGAAGAAAAATCTGATAGAGTTCCTCTTGCTGGTCACGAAGGCAAACTAGCTGATC

CTTTCAGTCAAATAGGCCATAATAGTGAGCCCAGCAGCACCCCTGAGCTACCTGCTTCTGGAGCGAATG

ACCATATTGGAGAAGGTGGCGTTTCCCAATTGAATAGAATTGCTGACGATGCTGATGATGACGATCCTTT

TGCCAAGAGGAGGAGGATGGAAGTTGGTGAAATGGACATCAACTCTGTGGTTAAGCCAATCCGAGAA

CCACGTGTCGTTGTCCAAACGGTGAGTGAGGTTGATATTCTTGATGATGGATACCGCTGGCGCAAGTAT

GGGCAGAAGGTGGTGAGAGGCAACCCAAATCCAAGGAGTTACTATAAGTGCACTCACCCCGGATGCC

CAGTAAGGAAGCACGTGGAGAGGGCATCCCATGACCCAAAATCAGTGATAACAACTTACGAGGGGAA

ACACAATCATGACGTACCAACTGCCCGGAATAGTAGCCATGACACAGTGGCTCAAGCAGCTTCAAGCC

TGGCAGCACGGGCTAGCTCACATGAAGGGACTGAAGGAGTGAGCCTTGATCTCGGAGTTGGGCTGAG

GCCAGGCCCTGACTTTGGGTCAGGCCACCAGACGGCTATGGCGGCTGAGCACATCGCTGGCGCTGCCC

ATTTTGGGAGTCCGAATCTTTTGTTACTTCAAGCGGCTCAGATTTCAAGTTTTCCTGGTTATGTAAATGG

TGGTATGGTGGTTTATGGTTCAACAAATAGGGAAAACCATAGTCAAGGGCATAGCATTGGAAATGGGAA

CCCGCCTGTAAATCACTCTTCTAATCCATATCAACAGAACACGGGAAGGATACTAATGGGTCCTTAG

>*HmoWRKY20*

ATGGACGGAGCTCCGGTAGCCTCCGGCAACACGCCAACATCATCCGGCGCCGGCGTTATGGACGGCTC

TGATCTGAGGAGCAGGGGGGCCCACAACGATGCCGGATCATCAACCATTAACGCCAAGTACAAGCTCA

TGTCGCCGGCCAAACTCCCGATCTCTCGTTCGCCTTGCATCACTATCCCTCCTGGCCTCAGTCCGACGT

CGTTTCTTGAATCCCCCGTCCTCCTTTCCAATTTCAAGGGAGAACCTTCGCCAACAACTGGTTCCTTTTT

CAAGCCTCAAATAATGCACAGCTCTTCTTGTGGGTCCACATTCCCTTCTATTATAAATAACTCTGATACCA

TGGATGGTAGTAATTCAAGCGGCTTTGAGTTCAAACCCGATTCTGGATCATTTTCTGCTGGAGGCTTTTC

TTTTGCAAGATCTCTGCTCCCATCTTCAGATTTGAACCGACAACAAGCTATGCAAGTCCAAGCAGAGTG

CCAGTCTCAATCATCTGATCCATCGACAACTGTTAAAAGCGAAGTAGGAGGCTCAACTGAATTGAGATC

TACTATGTTTACTCCAGGATCTGCTATACAGCCTGAAGTTGATTCCAATGAATTAAATCAAACAGCAGAC

TCCAATAATGAAGCCCAGGCACCACAAACTGAGCAGAAAGGAGTAGGGTCTCAAGTCATAACATCAGA

TGATGGGTACAATTGGAGGAAGTATGGACAGAAACTTGTCAAGGGAAGTGAATATCCACGAAGTTATT

ACAAATGTACGCATCCCAACTGTGAAGTCAAAAAGTTATTTGAGCGATCACCTGATGGACATATAACAG

AGATCATCTACAAGGGTACACATGATCATCCGAAGCCTCAACCTAATCGTCGACTCCCAACTGGCTCTA

TGATGTCCACCCTTGAAGAAAAATCTGATAGAGTTCCTCTTGCTGGTCACGAAGGCAAACTAGCTGATC

CTTTCAGTCAAATAGGCCATAATAGTGAGCCCAGCAGCACCCCTGAGCTACCTGCTTCTGGAGCGAATG

ACCATATTGGAGAAGGTGGCGTTTCCCAATTGAATAGAATTGCTGACGATGCTGATGATGACGATCCTTT

TGCCAAGAGGAGGAGGATGGAAGTTGGTGAAATGGACATCAACTCTGTGGTTAAGCCAATCCGAGAA

CCACGTGTCGTTGTCCAAACGGTGAGTGAGGTTGATATTCTTGATGATGGATACCGCTGGCGCAAGTAT

GGGCAGAAGGTGGTGAGAGGCAACCCAAATCCAAGGAGTTACTATAAGTGCACTCACCCCGGATGCC

CAGTAAGGAAGCACGTGGAGAGGGCATCCCATGACCCAAAATCAGTGATAACAACTTACGAGGGGAA

ACACAATCATGACGTACCAACTGCCCGGAATAGTAGCCATGACACAGTGGCTCAAGCAGCTTCAAGCC

TGGCAGCACGGGCTAGCTCACATGAAGGGACTGAAGGAGTGAGCCTTGATCTCGGAGTTGGGCTGAG

GCCAGGCCCTGACTTTGGGTCAGGCCACCAGACGGCTATGGCGGCTGAGCACATCGCTGGCGCTGCCC

ATTTTGGGAGTCCGAATCTTTTGTTACTTCAAGCGGCTCAGATTTCAAGTTTTCCTGGTTATGTAAATGG

TGGTATGGTGGTTTATGGTTCAACAAATAGGGAAAACCATAGTCAAGGGCATAGCATTGGAAATGGGAA

CCCGCCTGTAAATCACTCTTCTAATCCATATCAACAGAACACGGGAAGGATACTAATGGGTCCTTAG

>*HuWRKY21*

ATGGAGGGGGTTGAGGAAGCTAACAGAGGTGCCGTGGAAAGTTGTCATAGAGTTCTAAGCATCATATC

TCAATCTCAAGACCAAGCCCAGTTTAGGAAATTAGCTGCTGAAACTGAGCAAGCCTTGACTAAGTTCA

AAAGGGTTATATCTCTCCTAGGTAACGGATTAGGCCATGGGAGGGCTAGGATAGTGAAGAGAAATAGA

ACTAACCCTTATCCCCAAAATATTTTCTTAGAGTGGTCAAATGAGCCTAAAGCTGATCAGATTCAACCAA

TTGAGGCTCTTCAGCTACTCAAAACCAACTTGAATGAGTGCCCAATTCAAGAGAATGGCTCCACAACT

GGTAGAAGTTGTTTGTCTTTGGGGGGGAATCTGTCATTGGAACTGAGTTCAAGCGGAAAAACCCCTCT

TCAACTTGCTCAGGTGGTTCCGAAATCACATTATCAGTTCCTTCAGCCGCAGCAGAATCTCCAGCTTCA

ACAGCAACAGCTAAAGCATCAAGCTGAAATGATGTATAAGAGGAGTAATAGTGGGATTAATCTGAATTT

CAACTACTCTAGTTGCACTCCTACGATGTCTTCGACTCGATCTTTCATCTCTTCATTGAGTATTGATGGGA

GTGTGGCTAATTTGGATGGGAATGGGTTCAATTTGATTGGGGTCAGTCACTCATCAGACCAGAACTTGA

GCCAACAGAGAAAGAGGTGCTCTGGTAGGGGAGAAGATGGTAGTGTGAAATGTTCAAGTAGTGGTCG

ATGCCATTGCTCCAAAAAGAGGAAGCATAGGGTGAAGAGATCAATCAAGGTTCCTGCCATTAGTAACA
AACTGGCCGATATCCCTCCGGACGAGTATTCCTGGAGAAAATATGGTCAGAAGCCAATCAAGGGTTCA
CCTCACCCTAGGGGTTACTATAAGTGTAGCACCATGAGAGGTTGTCCCGCAAGGAAGCATGTCGAGAG
GTGCTTGGAAGATCCGTCTATGCTTATTGTTACATATGAAGGTGAGCACAATCACTCCTGTGTACCATCA
CCATCAGCAAACACATGA

>*HmoWRKY21*

ATGGAGGGGGTTGAGGAAGCTAACAGAGGTGCCGTGGAAAGTTGTCATAGAGTTCTAAGCATCATATC
TCAATCTCAAGACCAAGCCCAGTTTAGGAAATTAGCTGCTGAAACTGAGCAAGCCTTGACTAAGTTCA
AAAGGGTTATATCTCTCCTAGGTAACGGATTAGGCCATGGGAGGGCTAGGATAGTGAAGAGAAATAGA
ACTAACCCTTATCCCCAAAATATTTTCTTAGAGTGGTCAAATGAGCCTAAAGCTGATCAGATTCAACCAA
TTGAGGCTCTTCAGCTACTCAAAACCAACTTGAATGAGTGCCCAATTCAAGAGAATGGCTCCACAACT
GGTAGAAGTTGTTTGTCTTTGGGGGGGAATCTGTCATTGGAACTGAGTTCAAGCGGAAAAACCCCTCT
TCAACTTGCTCAGGTGGTTCCGAAATCACATTATCAGTTCCTTCAGCCGCAGCAGAATCTCCAGCTTCA
ACAGCAACAGCTAAAGCATCAAGCTGAAATGATGTATAAGAGGAGTAATAGTGGGATTAATCTGAATTT
CAACTACTCTAGTTGCACTCCTACGATGTCTTCGACTCGATCTTTCATCTCTTCATTGAGTATTGATGGGA
GTGTGGCTAATTTGGATGGGAATGGGTTCAATTTGATTGGGGTCAGTCACTCATCAGACCAGAACTTGA
GCCAACAGAGAAAGAGGTGCTCTGGTAGGGGAGAAGATGGTAGTGTGAAATGTTCAAGTAGTGGTCG
ATGCCATTGCTCCAAAAAGAGGAAGCATAAGGTGAAGAGATCAATCAAGGTTCCTGCCATTAGTAACA
AACTGGCCGATATCCCTCCGGACGAGTATTCCTGGAGAAAATATGGTCAGAAGCCAATCAAGGGTTCA
CCTCACCCTAGGGGTTACTATAAGTGTAGCACCATGAGAGGTTGTCCCGCAAGGAAGCATGTCGAGAG
GTGCTTGGAAGATCCGTCTATGCTTATTGTTACATATGAAGGTGAGCACAATCACTCCTGTGTACCATCA
CCATCAGCAAACACATGA

>*HuWRKY22*

ATGGAAGGCTTTGCAGATTGGGATCTTCTAGCGGTGGTCCGAAGTTGTTGCACAGCCACCGCCACCGC
GACCACCACCTCAGCGGTGGATGTCCACCCATCTCCCCCGCCTCCTCCGCCAGTGAAGGAAGAGCCCT
GTTTCCCTCTCCCAACTCCTGCTCCTCCTACTCCACCTTCTTCTTCTTCTTCCTCATCAAGTGAGCTTCTT
TTTGCGTTTCCCACTACCCTTTTTGACCCCCCAACGGCGTCGTTCTTTGGTGGGCAACAAGATTTGCAG
GATGTTTGTAAACCCAAATCCATTTGCACCACCAATTCTACCAATTTTCACCCAAACTCCTCCACTTCTT
CTTCTCTGACTTCCTCTGTTTCTTCATCGTCTTCTTCTTCTGTCATCGCTCGAAAATCTTTCGGAATCGGA
GAACAACCGGAGCTCCGGCGCCTAATTTGCCCTACTCAAGTTGTATTTGGGGCTGCTCCTGCTTCGACT

TCTCCAGCTCAAAGAACAAAAAAAAGGAAGAATTTGGTGAAGAAAGTGTTCCATGTACCAGCTGAAG
GTCTGTCAAATGACATGTGGGCATGGAGAAAATATGGTCAGAAACCCATCAAAGGTTCTCCATATCCCA
GGGGTTACTACAGATGCAGCAGTTCGAAGGGGTGTTTAGCCCGAAAACAAGTGGAGCGAAACAGATC
CGACCCGACAATGTTCATCGTCACTTACACCGCGGAGCACAACCACCCTATGCCTACCCATCGGAACTC
CCTAGCCGGAAGCACCCGACAGAAGTCCGCCGCTTCCGCCGATGTTCCGTCGCAAGATCAGCCGACCT
GCTCTTCCCCCTCGGCCGCCAGTCTCTCTCCTCCGATGGAGAAATCGGAGTCAAAAACAGAGGGCGAT
GATCTGATCAAGGAAGACGAAGACAACGACATGGGTCTTTCAGATCTGATTCTCAATGACGATTTCTAC
GTGGGTTTTGAAAAAATCGACGCCGATGAGTGCTTTTCCGACCACTTTTCTCCGCCGATGGAGCCTCCT
TGGCTAGCAAACAGCAGCAGCCCCGCCGCCGCAGCCGGTGGCAGCTGA

>*HuWRKY23*

ATGGAGAAGGAAGCAGTGTTGAAAATGGAGAGTTTGCTGGGAGGAGTAGGAGGAGGAGGAGGAGGA
GCTTCATTTAGTGGTGTTAGTACTACTGATTACCACTGTAATTTGGCTAAAAGTAGTCTTTCATCATCCAC
AACAACAACATCATCATCATCAACATCACCCAGTTTGGGGTTTATGGAGTCAATGAGTATGCAGGAAAT
CATGATTCATAATAACATTATCGGCCCTTCTCTGCTTGATATGGTCATCAATGGCTATGGTGTGGGTGGAA
ATCATCAGGTCTTGCCTGATTCTCTAGACCCTCCTGCACCTGATCCCACTCTTAATTCAAATGTCAACCC
AGATTCATTATCTGATCAAGTTGTTAATAATCATCAGCCTACAACACCAAACTCTTCTTCAGTGTCCTCT
GCTTCGAATGACGCGGCCAATGATGAACAACAACAGCAGCAGCAGAACAAGCCTGGTGATGGTGGGG
AGGAGGAGGAAGAAGAAGAAGAGGAACAGCAAAAAGACACCAAAGATTTGACAAAAGCCAAAAAG
ACAAATCAGAAGCGGCAGAGAGAACCCAGATTTGCGTTCATGACAAAGAGCGAGGTTGATCATCTAG
AAGATGGGTATCGATGGAGAAAGTATGGTCAAAAAGCTGTAAAAAACAGCCCTTTCCCAAGGAGTTAC
TACCGTTGCACCAGTGCATCATGCAATGTGAAGAAGAGAGTGGAGAGATCCTTCACTGACCCCTCCAT
AGTTGTCACTACCTATGAAGGCCAACACATCCACCCCTGTACCGTCCTTCCACGTGGCATCATGCCAGG
TCCCGGGTTCGGATTCAGTTTCGGCCGCGGCTCTGGTGGCGGTGGCGGTGCAACCACCACCACCACAT
CTCCCTATGCCTTGCAGCAGCAAATGCAACTTATGATGGCTAGTAATAATAGTCACATTCTACCTTCTAA
CATCCAAACAAGTTATGGTCGTCATGTTGCCTCTTTTGGCAATGTTTTAAGCACACCACATAATGCATTG
TTGTTTGCTCAAGAAAAGAGCTTCTGTGGTGCACCTGCTAATGCTGGTTTGATTCCTGATAATGGCCTTC
TTCAAGATATGATTCTTCCTAATCCCATTATGAAGACTGAAGAGTAG

>*HmoWRKY23*

ATGGAGAAGGAAGCAGTGTTGAAAATGGAGAGTTTGCTGGGAGGAGTAGGAGGAGGAGGAGGAGGA

GCTTCATTTAGTGGTGTTAGTACTACTGATTACCACTGTAATTTGGCTAAAAGTAGTCTTTCATCATCCAC
AACAACAACATCATCATCATCAACATCACCCAGTTTGGGGTTTATGGAGTCAATGAGTATGCAGGAAAT
CATGATTCATAATAACATTATCGGCCCTTCTCTGCTTGATATGGTCATCAATGGCTATGGTGTGGGTGGAA
ATCATCAGGTCTTGCCTGATTCTCTAGACCCTCCTGCACCTGATCCCACTCTTAATTCAAATGTCAACCC
AGATTCATTATCTGATCAAGTTGTTAATAATCATCAGCCTACAACACCAAACTCTTCTTCAGTGTCCTCT
GCTTCGAATGACGCGGCCAATGATGAACAACAACAGCAGCAGCAGAACAAGCCTGGTGATGGTGGGG
AGGAGGAGGAAGAAGAAGAAGAGGAACAGCAAAAAGACACCAAAGATTTGACAAAAGCCAAAAAG
ACAAATCAGAAGCGGCAGAGAGAACCCAGATTTGCGTTCATGACAAAGAGCGAGGTTGATCATCTAG
AAGATGGGTATCGATGGAGAAAGTATGGTCAAAAAGCTGTAAAAAACAGCCCTTTCCCAAGGAGTTAC
TACCGTTGCACCAGTGCATCATGCAATGTGAAGAAGAGAGTGGAGAGATCCTTCACTGACCCCTCCAT
AGTTGTCACTACCTATGAAGGCCAACACATCCACCCCTGTACCGTCCTTCCACGTGGCATCATGCCAGG
TCCCGGGTTCGGATTCAGTTTCGGCCGCGGCTCTGGTGGCGGTGGCGGTGCAACCACCACCACCACAT
CTCCCTATGCCTTGCAGCAGCAAATGCAACTTATGATGGCTAGTAATAATAGTCACATTCTACCTTCTAA
CATCCAAACAAGTTATGGTCGTCATGTTGCCTCTTTTGGCAATGTTTTAAGCACACCACATAATGCATTG
TTGTTTGCTCAAGAAAAGAGCTTCTGTGGTGCACCTGCTAATGCTGGTTTGATTCCTGATAATGGCCTTC
TTCAAGATATGATTCTTCCTAATCCCATTATGAAGACTGAAGAGTAG

>*HuWRKY24*

ATGACATATGACCCTCATGAATTTTTTCTTGATAACCATGATAATCAAGATGATCATGATAATGATGGCAG
CGTTATGGCACAAGTGTTGGAAATTCCCACGGTTCAGTCTCCATTGATCCTCGATGATATTTATCATACTA
ATTATGACATTAACAATGATCATCATGTTAATTATCATCCTGATCAAGACGATGATGATCATCATCATGATC
ATGCTAATAATGATGAGATTATCATCATAAATGATGATAATAGTAAGTTGACGAAGAAAGTATCATCATCA
TCGAAGAAGATAAAGAAGCATCGATATGCATTTCAAACAAGGAGTCAAGTTGATATTCTTGATGACGGC
TATAGATGGCGCAAGTATGGCCAAAAGGCGGTCAAAAATAACACCTTCCCAAGAGGGTATTATAGATGC
ACCTATCAAGGATGCAAGGTGAAAAAACAAGTTCAACGTCTATCGGATGACGAAGGGATTGTGGTCAC
CACTTATGAAGGGGTTCACTCTCATCCAATTCAGAAGTCTACAGATAACTTTGAGCATATCTTGAGCGA
AATGCAAATTCACCCTTCTTGTTGA

>*HmoWRKY24*

ATGACATATGACCCTCATGAATTTTTTCTTGATAACCATGATAATCAAGATGATCATGATAATGATGGCAG
CGTTATGGCACAAGTGTTGGAAATTCCCACGGTTCAGTCTCCATTGATCCTCGATGATATTTATCATACTA
ATTATGACATTAACAATGATCATCATGTTAATTATCATCCTGATCAAGACGATGATGATCATCATCATGATC

ATGCTAATAATGATGAGATTATCATCATAAATGATGATAATAGTAAGTTGACGAAGAAAGTATCATCATCA
TCGAAGAAGATAAAGAAGCATCGATATGCATTTCAAACAAGGAGTCAAGTTGATATTCTTGATGACGGC
TATAGATGGCGCAAGTATGGCCAAAAGGCGGTCAAAAATAACACCTTCCCAAGAGGGTATTATAGATGC
ACCTATCAAGGATGCAAGGTGAAAAAACAAGTTCAACGTCTATCGGATGACGAAGGGATTGTGGTCAC
CACTTATGAAGGGGTTCACTCTCATCCAATTCAGAAGTCTACAGATAACTTTGAGCATATCTTGAGCGA
AATGCAAATTCACCCTTCTTGTTGA

>*HuWRKY25*

ATGGGGGACAGTGAAGATGCATTGGATGAGTGGAGGGATCTTGTGGTGAGTCCTAAGGGACTATTCTC
AGTCTTTTCACCTGAGCGCTGTGTTACAAAATCAATTGCTGATGTTTTCAGTGATCGGAAGGGGATTTG
GGGGGGTGATGATGACAAAGAAATGGAGAATTCAGTGTTTGATTTGGGGAAGAAGTTGGAGGATTGTG
GGCAGGGATGGGATTCTTCGATTGTCGAGGATGATTTGGATGTGCCCATGGCAGGAGGGAGCATAGTG
GATACGAGGGCTGCTAAGTTGGGGTTCAATGCTCCTAACGCAAGTTCCCCTCCTTTTAGCTCTAGCAGC
CCGTTGCCCTCCCCAGGTTCTCGATCACCCTATCTTATCGCACCTGCTAGTATTAGCCCTACTGGACTGC
TTGACTCTCCACCTTTGCTGCCAAATTCACAGCCTTCTCCAACCACAGGAACTTTTGAATCACCTCCGG
AGGGTGGAAACAATCAGATGGAGATTCCAACATCTTCTGCAGGCACGGACAAGCACAATGATGGTGGC
ACTACAACCATGATTAAGGCCAATGATGCCATTCCTATCACATGTTTCTGTGCCGTAGAAAATGAGGGGT
CCCCTGGTGGTTTGGCTCAGAGTTTGGTTGCTCGTGTTCATGATCAAAGTATTAACACAATGCCGCTAC
CAGTGAACTTCGAAATTCCAATGCAGTTTCCTAATGAAGTTGCTATGAAAAGTCATGAAATTGGCTCAC
CAAATGATGAACACTTGTCGGATGACATCACTGTAGACTCCAACCCCACCACTCTGCAACCATGCCATT
CGACGACTTTGGTATGTGATCAATTGCAGGAAAAGGGCGAACCGTCTCTGGAAGAGGATGCTGATACT
TATAATGCCCTGGATTCAAAACACAAAGGAACATATGCTCCAACAGGGACAGTGAGAACCACAGAAGA
TGGGTACAATTGGAGAAAATATGGACAGAAACAAGTCAAGGGTAGTGAATATCCACGCAGTTACTATA
AATGCACCCACCCAACCTGTCAAGTGAAGAAGAAGGTAGAACGTTCTCATGATGGCCAAATTACAGAG
ATTATATACAAGGGTGGCCATAACCATCCAATGCCCCAACCCTGTAGGAAATCAGCATTTAGTTCATCAT
TATCATTTCGTGAGACATCAGATATATGTGATGGTAGTAGCTCTTGCGTCAAAACTGAGAGTGAATCAAC
CTGGAGAAACATGCAGCCAGGGAGTAAAGGTGGCATGCCTGGGTCTGACTGGAGGACTGATGGCTTG
GACAGGGCATCCTCTACCTCTGTTGTGACTGAAGTTTCTGATATCCTGTCCACCAATCAAGAAAAACCC
TTATGCATGCTTGAATCAGCAGGAAGTCCAGAGTTCTCATCTACAATTGCTATTAATGAGGATGATGAAG
AAGATACTACCACCCAAGGGGGTTGTTTACTTGCAGATGATGATGACAATAAATCAGACTCAAAAAGA
AGGAAAAAGGAGTCTTGTTTTTATGAAACAACTTTAGGTTCCAGGGCTGTTCGCGAACCAAGAGTTGT

TATCCAAATAGAGAGTGATATTGATATACTAGATGATGGGTATCGCTGGCGCAAGTACGGGCAGAAGGT
TGTGAAGGGGAATCCAAATCCTAGGAGCTACTACAAATGCACCCGTCCTGGATGCCCTGTCAGGAAGC
ATGTGGAAAGAGCCTCACACGACTTGAAATTTGTGATAACAACGTATGAAGGAAAACACAATCATGAA
GTCCCTGCTGCTAGAAATAGCTGTCATGCCAATTCAAGCAGTGGAGGCTTGCCCCCTCCCACATCCAGT
ACACAGCCTACCCTGAACTTACCGCGAAACCCTCATCTTCCAAAGCCGGAGTCTCATGTACAAGATCTC
CCCCCTCATTTTGGCAGAAAATTGGAGTGCAATAGTGACTTCTTAGGAGCGAACTTTCTAGGGAATTTT
GGAGGCGGAGCTTCCTCATTTTATCAAATGAAGTTTCCAGCCTTACCGAATGCTGTACCGTATGGTCCAT
TTAGTTTGAGTAATCACCATCCAACTTTCCCCGGCAATGTGGTGCCAGGCTTCACAGATCTTTCCCTCCC
ATTGCCACTCGGATTCCCCCATTCCTCAAATTTAGCCATTGCCAACTATACCAATCCTGGAAGACCACTT
GCTTCAGTCCAGCCCTTCATGGCAGGCCATCACCATGAGGAACCTAACTCCAAGATCCTTAGACCTAAG
CAGGAACAGGGAGATGACTCATTTTGTGATACAGGAATGCATCACATCGACCATACCATGTCTTCATCAT
CATCTATATACAGCCAAATTGTGTGA

>*HmoWRKY25*

ATGGGGGACAGTGAAGATGCATTGGATGAGTGGAGGGATCTTGTGGTGAGTCCTAAGGGACTATTCTC
AGTCTTTTCACCTGAGCGCTGTGTTACAAAATCAATTGCTGATGTTTTCAGTGATCGGAAGGGGATTTG
GGGGGGTGATGATGACAAAGAAATGGAGAATTCAGTGTTTGATTTGGGGAAGAAGTTGGAGGATTGTG
GGCAGGGATGGGATTCTTCGATTGTCGAGGATGATTTGGATGTGCCCATGGCAGGAGGGAGCATAGTG
GATACGAGGGCTGCTAAGTTGGGGTTCAATGCTCCTAACGCAAGTTCCCCTCCTTTTAGCTCTAGCAGC
CCGTTGCCCTCCCCAGGTTCTCGATCACCCTATCTTATCGCACCTGCTAGTATTAGCCCTACTGGACTGC
TTGACTCTCCACCTTTGCTGCCAAATTCACAGCCTTCTCCAACCACAGGAACTTTTGAATCACCTCCGG
AGGGTGGAAACAATCAGATGGAGATTCCAACATCTTCTGCAGGCACGGACAAGCACAATGATGGTGGC
ACTACAACCATGATTAAGGCCAATGATGCCATTCCTATCACATGTTTCTGTGCCGTAGAAAATGAGGGGT
CCCCTGGTGGTTTGGCTCAGAGTTTGGTTGCTCGTGTTCATGATCAAAGTATTAACACAATGCCGCTAC
CAGTGAACTTCGAAATTCCAATGCAGTTTCCTAATGAAGTTGCTATGAAAAGTCATGAAATTGGCTCAC
CAAATGATGAACACTTGTCGGATGACATCACTGTAGACTCCAACCCCACCACTCTGCAACCATGCCATT
CGACGACTTTGGTATGTGATCAATTGCAGGAAAAGGGCGAACCGTCTCTGGAAGAGGATGCTGATACT
TATAATGCCCTGGATTCAAAACACAAAGGAACATATGCTCCAACAGGGACAGTGAGAACCACAGAAGA
TGGGTACAATTGGAGAAAATATGGACAGAAACAAGTCAAGGGTAGTGAATATCCACGCAGTTACTATA
AATGCACCCACCCAACCTGTCAAGTGAAGAAGAAGGTAGAACGTTCTCATGATGGCCAAATTACAGAG
ATTATATACAAGGGTGGCCATAACCATCCAATGCCCCAACCCTGTAGGAAATCAGCATTTAGTTCATCAT

TATCATTTCGTGAGACATCAGATATATGTGATGGTAGTAGCTCTTGCGTCAAAACTGAGAGTGAATCAAC

CTGGAGAAACATGCAGCCAGGGAGTAAAGGTGGCATGCCTGGGTCTGACTGGAGGACTGATGGCTTG

GACAGGGCATCCTCTACCTCTGTTGTGACTGAAGTTTCTGATATCCTGTCCACCAATCAAGAAAAACCC

TTATGCATGCTTGAATCAGCAGGAAGTCCAGAGTTCTCATCTACAATTGCTATTAATGAGGATGATGAAG

AAGATACTACCACCCAAGGGGGTTGTTTACTTGCAGATGATGATGACAATAAATCAGACTCAAAAAGA

AGGAAAAAGGAGTCTTGTTTTTATGAAACAACTTTAGGTTCCAGGGCTGTTCGCGAACCAAGAGTTGT

TATCCAAATAGAGAGTGATATTGATATACTAGATGATGGGTATCGCTGGCGCAAGTACGGGCAGAAGGT

TGTGAAGGGGAATCCAAATCCTAGGAGCTACTACAAATGCACCCGTCCTGGATGCCCTGTCAGGAAGC

ATGTGGAAAGAGCCTCACACGACTTGAAATTTGTGATAACAACGTATGAAGGAAAACACAATCATGAA

GTCCCTGCTGCTAGAAATAGCTGTCATGCCAATTCAAGCAGTGGAGGCTTGCCCCCTCCCACATCCAGT

ACACAGCCTACCCTGAACTTACCGCGAAACCCTCATCTTCCAAAGCCGGAGTCTCATGTACAAGATCTC

CCCCCTCATTTTGGCAGAAAATTGGAGTGCAATAGTGACTTCTTAGGAGCGAACTTTCTAGGGAATTTT

GGAGGCGGAGCTTCCTCATTTTATCAAATGAAGTTTCCAGCCTTACCGAATGCTGTACCGTATGGTCCAT

TTAGTTTGAGTAATCACCATCCAACTTTCCCCGGCAATGTGGTGCCAGGCTTCACAGATCTTTCCCTCCC

ATTGCCACTCGGATTCCCCCATTCCTCAAATTTAGCCATTGCCAACTATACCAATCCTGGAAGACCACTT

GCTTCAGTCCAGCCCTTCATGGCAGGCCATCACCATGAGGAACCTAACTCCAAGATCCTTAGACCTAAG

CAGGAACAGGGAGATGACTCATTTTGTGATACAGGAATGCATCACATCGACCATACCATGTCTTCATCAT

CATCTATATACAGCCAAATTGTGTGA

>*HuWRKY26*

ATGGAAGAACTGAAGAAGCAAAATGACATGAATAATAACAAAGAGCAACCCGGTTCCCACTCCGGGAT

AAACCGGGACCAAGAAACAGAATCCAGTGCACAAAAGAATTACAACAGCTCTCAGCCTCAGCAACAA

CCACCACAACCAGTAGTGCATATGAACAGAGCACAGAAGCGATCAGAGGACGGCTACTGCTGGAGGA

AGTACGGCCAGAAGCAAGTGAAGGGGAGTGAGAATCCCCGGAGCTACTACAAGTGCTCGTACCCTGG

TTGCCCAACAAAGAAGAAGGTCGAGCGGTCTCCTGAAGGGTTCATCACTGAGATTGTCTACAAGGGTA

GTCACAACCATCCTAAGCCTCAACCTGGGAGAAGATCTTCAGGGCAGAATGGCAACAATGGTGGGTTT

GATGGCTCCGAGGTGTTCAACTCTTCGGGTTTTCAAGGGCAAAGTGAGTACTCATCTTTCACTCCTGAG

ACATCCTCGGTTTCATTGGAAGATGATGATGAGCTTGATCAGACTTCTGCAATGAGTAAGTCGTGCAAG

AATGATGACAATGAACCTGAAGCTAAAAGATGGAGGGGTGAGAATGAGGGTGAGGTCATGTCAGGGTA

TGAGAGCAGAACAGTGAGAGAGCCAAGAGTGATAGTTCAAACGACAAGCGAGATCGATATTCTTGATG

ATGGGTATAGATGGAGAAAATATGGGCAGAAAGTGGTAAAAGGCAACCCGAACCCTAGGAGCTACTAT

AAATGCACAAGTGCGGGTTGCCCAGTGAGGAAGCACATAGAGAGGGCTTCCCATGACTTGAGGGCCG
TCATCACAACCTACGAAGGGAAGCACAACCACGATGTCCCAGCAGTGCGTGGTAGCAGCAGCTACAAT
AACGCCCCTAACCTGATTAGGCCATCGGCCACTACTGGTCCACCAAATCAACTAACTGCCCCAAAGCCC
CTCGATAACAGTGGTGCAAGTACCTCCAGAGTGGATACTCAGTCTCCCTTCAGCTTGCAGATGTTGCAG
AGCCCTGGGAGCTTCGATTTCTCCGGGTTTGGGAATCCATTGGGAACCAAGGAGGAGCCTGTAGACAA
TACATTTCTAGAGTCACTTCTTCGTTAA

>*HmoWRKY26*

ATGGAAGAACTGAAGAAGCAAAATGACATGAATAATAACAAAGAGCAACCCGGTTCCCACTCCGGGAT
AAACCGGGACCAAGAAACAGAATCCAGTGCACAAAAGAATTACAACAGCTCTCAGCCTCAGCAACAA
CCACCACAACCAGTAGTGCATATGAACAGAGCACAGAAGCGATCAGAGGACGGCTACTGCTGGAGGA
AGTACGGCCAGAAGCAAGTGAAGGGGAGTGAGAATCCCCGGAGCTACTACAAGTGCTCGTACCCTGG
TTGCCCAACAAAGAAGAAGGTCGAGCGGTCTCCTGAAGGGTTCATCACTGAGATTGTCTACAAGGGTA
GTCACAACCATCCTAAGCCTCAACCTGGGAGAAGATCTTCAGGGCAGAATGGCAACAATGGTGGGTTT
GATGGCTCCGAGGTGTTCAACTCTTCGGGTTTTCAAGGGCAAAGTGAGTACTCATCTTTCACTCCTGAG
ACATCCTCGGTTTCATTGGAAGATGATGATGAGCTTGATCAGACTTCTGCAATGAGTAAGTCGTGCAAG
AATGATGACAATGAACCTGAAGCTAAAAGATGGAGGGGTGAGAATGAGGGTGAGGTCATGTCAGGGTA
TGAGAGCAGAACAGTGAGAGAGCCAAGAGTGATAGTTCAAACGACAAGCGAGATCGATATTCTTGATG
ATGGGTATAGATGGAGAAAATATGGGCAGAAAGTGGTAAAAGGCAACCCGAACCCTAGGAGCTACTAT
AAATGCACAAGTGCGGGTTGCCCAGTGAGGAAGCACATAGAGAGGGCTTCCCATGACTTGAGGGCCG
TCATCACAACCTACGAAGGGAAGCACAACCACGATGTCCCAGCAGTGCGTGGTAGCAGCAGCTACAAT
AACGCCCCTAACCTGATTAGGCCATCGGCCACTACTGGTCCACCAAATCAACTAACTGCCCCAAAGCCC
CTCGATAACAGTGGTGCAAGTACCTCCAGAGTGGATACTCAGTCTCCCTTCAGCTTGCAGATGTTGCAG
AGCCCTGGGAGCTTCGATTTCTCCGGGTTTGGGAATCCATTGGGAACCAAGGAGGAGCCTGTAGACAA
TACATTTCTAGAGTCACTTCTTCGTTAA

>*HuWRKY27*

ATGAACGGCTCAGCTCTTGAGCCCTCCTCGTTCGGTTCGTTCTTTCCTTACAACAACACTGATTACTCAT
TTGACCATCATGACCTCGTAGATTTCTACAAGCCGTTCTTGGTCCAGCCGTCACCCCTTGGTGGCACTG
GTGCCGACGTTCCTGACCCTCAGGTTATTGGTTCGTTACCAAAAGCTGACACTGGGTTCGGACCTGATT
CTGGTGGGTTCCTAACGTTAGAACCCCACCAGAATCCACACCAGCAGCATCAACTAATACCGGCACCA
GTAGTTGGTGGTGGTGGTACTTTTCAGCAGCATGATGATCAACAGCTTCTTCTCCGGCAGCGAAGTCTT

CAGAATCGAAAGCTGCAGATACCAGGGGCTTGCCCTGTTGGCATCCATAAGAAAAAGAAGTTTGAGGA
GAGGAGAGTGTGCAAGATGAGACTAGAAGAGTTATCTGAGGACAAATGGGCATGGCGGAAATACGGG
CAAAAACCCATCAAGGGCTCTCCATATCCAAGGAACTACTACAGGTGCAGCACCTCAAAAGGATGCAC
AGCAAGGAAGCAAGTGGAGCGGAGCCCCGTTGAACCGAATACCTACCTGGTCACCTACACAGGTCAC
CACCACCATGCCCGGCCACCCCTCCGGACCCCAGCCCGGGTCACCCGCCCATCGGACTCCACCAGCTC
GCCTGAGGAAGAATCCAAATCTCAGACCAGTACTTCTGCATCCCCTACCCCGACCCTGGCTCCTTATCA
AAACGGGTCGGGTTCCGGTTCCGGGGAACCCGTGCTGGGGAAGAATGAGAATGAGAATGAGGTGGAG
GAAGGGGATGATGATGAGGAGATTGAGCTGATTCCAAACTCGATGGCGGACCCGGAACTGCTGATGGG
TATGGAGCGCCTGATGAACAATGATCAAAAGACATCATCAGGAGAGAGAAAAACTGATGAGACTGTTG
GAAATGGGTATGGGTATGAGTACGGGTACGGATATGGATATGACATGATGATGAGGGAGATCGGGGCAT
CCGACCAGAATGTGAATTTCGGGGTGGTGGTGAGAGGAAATGGGTTGTGTCTTGGGATCCAACAAAAG
GAATTGGATTCTTCTTCATCTGGTTCTGCTGCACCAACAACGCTGGCGGCGGCGCATGAGTCGCCGAAT
GGTCGGGGCCGCAACATTTGGGATAACGGGATCATTAACCCTCAATCCTTCCTATTTTAA

>*HmoWRKY27*

ATGAACGGCTCAGCTCTTGAGCCCTCCTCGTTCGGTTCGTTCTTTCCTTACAACAACACTGATTACTCAT
TTGACCATCATGGCCTCGTAGATTTCTACAAGCCGTTCTTGGTCCAGCCGTCACCCCTTGGTGGTACTG
GTGCCAACGTTCCTGACCCTCAGGTTATTGGTTCGTTACCAAAAGCTGACACTGGGTTCGGACCTGATT
CTGGTGGGTTCCTAACGTTAGAACCCCACCAGAATCCACACCAGCAGCATCAACTAATACCGGCACCA
GTAGTTGGTGCTACTTTTCAGCAGCATGATGATCAACAGCTTCTTCTCCGGCAGCGAAGTCTTCAGAAT
CGAAAGCTGCAGATACCAGGGGCTTGCCCTGTTGGCATCCATAAGAAAAAGAAGTTTGAGGAGAGGA
GAGTGTGCAAGATGAGACTAGAAGAGTTATCTGAGGACAAATGGGCATGGCGGAAATACGGGCAAAA
ACCCATCAAGGGCTCTCCATATCCAAGGAACTACTACAGGTGCAGCACCTCAAAAGGATGCACGGCAA
GGAAGCAAGTGGAGCGGAGCCCCGTTGAACCGAATACCTACCTGGTCACCTACACAGGTCACCACCA
CCATGCCCGGCCACCCCTCCGGACCCCAGCCCGGGTCACCCGCCCATCGGACTCCACCAGCTCGCCTG
AGGAAGAATCCAAATCTCAGACCAGTACTTCTGCATCGCCTACCCCGACCCTGGCTCCTTATCAAAACG
GGTCAAGTTCCGGTTCCGGTTCCGGGTCAGGGGAACCCGTGCTGGGGAAGAATGAGAATGAGAATGA
GGAGGAGGAAGGGGATGATGATGAGGAGATTGAGCTGATTCCGAACTCGATGGCGGACCCGGAACTG
CTGATGGGTATGGAGCGCCTGATGAATAATGATCAAAAGACATCATCAGGAGAAAGAAAAACTGATGA
GACTGTTGGAAATGGGTATGGGTATGGGTATGAGTACGGGTACGGATATGGATATGACATGATGATGAG
GGAGATCGGGGCACCCGACCAGAATGTGAATTTCGGGGTGGTGGTGAGAGGAAATGGGTTGTGTCTTG

GGATCCAACAAAAGGAATTGGATTCTTCTTCATCTGGTTCTGCTGCACCAACAACGCTGGCGGCGGCG

CATGAGTCGCCGGATGGTCGGGGCCGCAACATTTGGGATAACGGGATCATTAACCCTCAATCCTTCCTA

TTTTAA

>*HuWRKY28*

ATGGATGCACAAGAATTCTTATTATCATCATCATCATCAGATCATGCCCTAATGGCACCATATAATGCTGT

TGTTGAAGATCATGATCATCATCAAATCATGACAACTATGATGATCCCCTCTCATAATCATGATCATGGTA

ATGATGATCATGAGTTTGTGATCCCTACAAACTCATCATCATCTTCAATATCGGCGCCCAACAACAACAA

CAACAACATGTTGTTCTCCCCATTACTGCCAATGCAAACTCCTCAAACACCCTATACCTTGGACAATATT

GACTGGGTTAGTCTGCTCTCTCCTCCTACTCCTGCTGAGGTGGCGCCTTTCAGGCTGATCTCGCTACCC

ATGGTATCATCCTCTTCACCAGTTCCTACAATCAACAGCAGCAGCAACAACAACAACGACAACGACAA

CAACAAGAACAACAACAATGGCGGCGGCGGTGGTGGTGGTGGTAGTGGTCATGATCATCAAATGAGG

GGAGACAGATCAATGGCTGAGATTAATGGAGGTGGAAGTAGTAATAATAGTAAAGGAAATAGAGGAGG

GCTAAGTAGTTCTTCAAGTTATAATTCTAGTAATTATAATAATTCAACAATGAAGAAAAAGGGGGTGACT

CCGAGGGTGGCCTTCTATACAAAGAGTAATGAGGATGTTCTTGATGATGGTTATAAGTGGCGGAAATAT

GGCCAGAAATCTGTTAAAAATACCGTCCATCCCAGATTCAACGACACTCAAGAGATAAAAGCATTGTG

A

>*HmoWRKY28*

ATGGATGCACAAGAATTCTTATTATCATCATCATCATCAGATCATGCCCTAATGGCACCATATAATGCTGT

TGTTGAAGATCATGATCATCATCAAATCATGACAACTATGATGATCCCCTCTCATAATCATGATCATGGTA

ATGATGATGATCATGAGTTTATGATCCCTACAAACTCATCATCATCTTCAATATCGGCGCCCAACAACAA

CAACAACAACATGTTGTTCTCCCCATTACTGCCAATGCAAAATCCTCAAACACCCTATACCTTGGACAAT

ATTGACTGGGTTAGTCTGCTCTCTCCTCCTACTCCTGCTGAGGTGGCGCCTTTCAAGCTGATCTCGCTAC

CCATGGTATCAGCCTCTTCACCAGTTCCTACAATCAACAGCAGCAGCAACAACAACAACGACAACAAC

AAGAACAACAACAATGGTGGTGGTGGTGGTGGTGGTGGTAGTCATGATCATCAAATGAGGGGAGACA

GATCAATGGCTGAGATTAATGGAGGTGGAAGTAGCAATAATAGTAAAGGAAATAGAGGAGGGCTAAGT

AGTTCTTCAAGTTATAATTCTAGTAATTATAATAATTCAACAATGAAGAAAAAGGGAGTGACTCCGAGG

GTTGCCTTCTATACAAAGAGTAATGAGGATGTTCTTGATGATGGTTATAAGTGGCGGAAATATGGCCAGA

AATCTGTTAAAAATACCGTCCATCCCAGGAGCTATTATCGTTGCACATATCACACATGCAATGTGAAGAA

ACAGATTCAACGACACTCAAGAGATAAAAGCATTGTGAAAGGGCAATTCCAGCACACTGGCGGCCGTT

ACTAG

>*HuWRKY29*

ATGGAAGCTCCTCCAGATTGGGATCTACTCGCTGTGGTCCGAAGCTGCAGAACCTCCACCGCCGCCGT
CACATCAGCCGCTTTCAATGCGGCGGAGCTTCGACTTCCGCCGCCGGCACCGCCGCAGCTACCCTGCA
GCTTCTCTCCTTCGACGACCACCTCTGCTTCTTCTTCATCAACTGGAGTTGTGTTCCGATTTCCGAACCC
TTTTGAGGCCGCGGCGACGTCGTTTCAGGACTTGCAGGATATCTGCAAACCCTTGATTTGCACCACCAC
AACCACCACCACTAGCAGCAATTGCACCGCCGATGCCGCTGCCGCCGCCGTGACCGGCACTTTCCAGG
CGTCTTCTCTCTTATCCCCTGTTTCTTCTTCGTCTTCTTCTTTGATTGCCGCCGGCAGACCCTTCACAATC
GCCGAACAGCCGCGGCTAGGGCGGCCCAGTAAGCCGTCTCAGGTCATCTTTGGAGCTCCTTCTGTTAC
TGCTGGTACTTCTCAGGCTCAAAGGTCAAAGAAAAGGAAGAACTTGATGAAGAAGGTGTGCCATGTT
CCAGCTGAAGGGCTTTCCTCTGATATGTGGGCTTGGAGAAAATATGGTCAAAAACCCATCAAGGGCTCT
CCATATCCAAGGGGATACTACAGATGTAGCAGCTCAAAAGGCTGTTTGGCCCGAAAACAAGTGGAGCG
GAACAGATCCGACCCGAAAATGTTCATAGTCACTTATACCGCCGAGCACAACCACCCTATGCCCACTCA
CCGGAACTCCCTCGCCGGAAGCACGCGCCAGAAGCCCCTCTCCACCACCACCAACGACGCTTCAGCC
GCCGCAACTCCGCCGGAGCAGCCCACCTGCTCATCCCCCTCCGCTGCCAGTCTCTCTCCTCTGACGGA
GAAATCGGAGTCGAAGAATGAAGCCGACGACGTCGTAAAGGAGGAGAACGAAGAAGATAACGAGAT
GGGCATCTCTGATTTGATGTTAAACGACGATTTCTACGTGGGTTTTGAGCAGATCGATGGTGATTATGGC
GGGGATTGTTTTTCCGACCATTTCTCGCCGCCGATGGAGTTCCCCTGGCTTTCTAGTAATAGCGGCACC
GCCACCGCGGCCGGCGGGAGTTGA

>*HuWRKY30*

ATGGAACAAGCCTTGTTGATTACCAAGGAGCTAACCGAGGGCATGGAGAAGGCTATACAGCTAAAAGC
TCATATGGGTCAGCAAGTTTCAGGTAAGATTGAGGGAATGCTGATAGAAGGGATACTATCTTCCTTTGA
GAATGCCCTCTTGATTCTGAAATGGGGTGGGTCAATGGAACAGTCTCACCAACATGTGGTGCCATCGAC
CAGTATGCCCGGATCACCACAGTCGGTCATTGGGAGTCCCAAGAGTGAGGATTTGAACCCCGGTTTTA
CCACTCAACCAGGCAGCAGAGATTCATCTAGGAAGAGAAAGGCGATGCCGACTTGGACTGAACAAGG
GCAAGCTAGACCTGAGAATGGGATGGAAGGACCGCCTGAGGACGGTCATAGTTGGAGGAAGTACGGT
CAGAAGGACATTTTGGGAGCCAAGTATCCTAGAAGCTACTACAGATGCACATACCGCGACATGCAAGG
ATGTTGTGCTATCAAACAAGTGCAGCGAAATGATGAAAACCCTTTGGTATTTGACATCACCTATAAAGG
GAAGCACACATGTACGCCGACCCCACGTTCAAAGTCTGCGGCATTATCATCTGAAAAACAGGAATGCA
ATAGACGACATAACATTGCCATCACAGAATACCAGCAGCAGCCTGATGTGACGCTGAATTTCCAGACAG

GGCCAACCATTGATATACAAAACATAGAACATCAACAGTTGGGCCTCCCATTGACTCCAGAGGGATTAG

GATATGAAATTCATACCTTGGCACCATCAGCACTTGATGATAATAACTTCTATAGTAGTTTTCCACCATCT

TTCATACCATCTCCAGCTACCTCAGAATCAAACCACTTCTCTACGTCGCCCTGCAAAATGAGCACCTTT

GGAGGAGCTCAACATGGACAGCAATCTGAATCAGATATTACAGAGATAATTTCAGCCACTACTTCAATA

ACCAACTCCCCAATTGTTGGTGTACATTTTCCACTAGAACAATGGGACTTCAGCCTGAGTTTCCCATTT

GACAACCCAACTTTTTTCCCTAGTTTCTATTAA

>*HmoWRKY30*

ATGGAACAAGCCTTGTTGATTACCAAGGAGCTAACCGAGGGCATGGAGAAGGCTATACAGCTAAAAGC

TCATATGGGTCAGCAAGTTTCAGGTAAGATTGAGGGAATGCTGATAGAAGGGATACTATCTTCCTTTGA

GAATGCCCTCTTGATTCTGAAATGGGGTGGGTCAATGGAACAGTCTCACCAACATGTGGTGCCATCGAC

CAGTATGCCCGGATCACCACAGTCGGTCATTGGGAGTCCCAAGAGTGAGGATTTGAACCCCGGTTTTA

CCACTCAACCAGGCAGCAGAGATTCATCTAGGAAGAGAAAGGCGATGCCGACTTGGACTGAACAAGG

GCAAGCTAGACCTGAGAATGGGATGGAAGGACCGCCTGAGGACGGTCATAGTTGGAGGAAGTACGGT

CAGAAGGACATTTTGGGAGCCAAGTATCCTAGAAGCTACTACAGATGCACATACCGCGACATGCAAGG

ATGTTGTGCTATCAAACAAGTGCAGCGAAATGATGAAAACCCTTTGGTATTTGACATCACCTATAAAGG

GAAGCACACATGTACGCCGACCCCACGTTCAAAGTCTGCGGCATTATCATCTGAAAAACAGGAATGCA

ATAGACGACATAACATTGCCATCACAGAATACCAGCAGCAGCCTGATGTGACGCTGAATTTCCAGACAG

GGCCAACCATTGATATACAAAACATAGAACATCAACAGTTGGGCCTCCCATTGACTCCAGAGGGATTAG

GATATGAAATTCATACCTTGGCACCATCAGCACTTGATGATAATAACTTCTATAGTAGTTTTCCACCATCT

TTCATACCATCTCCAGCTACCTCAGAATCAAACCACTTCTCTACGTCGCCCTGCAAAATGAGCACCTTT

GGAGGAGCTCAACATGGACAGCAATCTGAATCAGATATTACAGAGATAATTTCAGCCACTACTTCAATA

ACCAACTCCCCAATTGTTGGTGTACATTTTCCACTAGAACAATGGGACTTCAGCCTGAGTTTCCCATTT

GACAACCCAACTTTTTTCCCTAGTTTCTATTAA

>*HuWRKY31*

ATGGAAACAACAGGATGGGGACTTGCCCTTGAATCTGATCATCATCATCACCGTCATCACCATCATCGTA

TTCCTCCTCCTCATCATCATCACGCTAGTCTCCTCGCCTTGAAAGGTGGAGGAAGCAGTAACAATCGGC

TGTTCCCTGGTGTAGACTTCCCCATGAATCTCAACCGTCGAGATTACCATGATCATGATGATGATCAGAC

TGGCCATAAGGTGGTAGTGGGTGAAGTTGATTTCTTCTCTGATCAAAAGAGTGCAAAGAGGACGTCAT

CACCTCATCATAGTAGTTATCATGCCAAGGAGGAGGGTTGTCCTCGAGATCACCATCATCATCCTGCTCC

AGCTGCCCCTGATTCCAACCCTGACCTGCTCCATGTAAATACTGGCCTACATCTCCTCACGACAAACAT

GGGAAGCGATCAATCTACTGTAGATGACAGTGCCTCTTCAGATGCAGAGGACAAGAGAGCCAAAAATG
AGTTAGCAGCACTTCAAGCCGAGCTAGAGCGAATGAATGCCGAAAACCAGAGGTTAAAGAACATGCTA
TCTCAAGTGAGCCACGACTACAGTAACCTTCAAGTGCACTTCGTATCTTTGATGCAACAACAACAACAT
CAAAAGGGTGAAAGCACCCAATCACATGAGGTGATTCAAGGGAAGGGGGAGGGAAGGAGGAACATA
GTGCCGAGGCAGTTCTTAGACATGGGTCCGAGCTCAAACAATGACACCGATGAGCTGACCTCTCAAAC
ATTATCCGATGATGAGCAAACCCTTGATGGTTCACCCCATAACAAAGAGGGTAATGGCTCGTACGAGCA
TGGCAAAGGTGGTAGTCATGGAAGAGAAGATAGCCCTGAAGACGCTGAGTGGGTACCTAATAAGGCTC
CCAAGCTGAACCTACCAAAGGGTAATAATGGCCCTGTTGATCAAGCTGCCGCGGAAGCTACCATGAGG
AAGGCTAGAGTCTCCGTTCGAGCCCGGTCTGAGGCGCCCATGATAAGTGATGGATGCCAGTGGAGAAA
ATATGGACAGAAGATGGCTAAGGGTAACCCTTGCCCTCGAGCTTATTATCGTTGCACAATGGCCGTCGG
TTGTCCTGTTCGCAAACAAGTTCAAAGATGTGCAGAGGACAGATCAATACTAATTACAACGTACGAGG
GTAATCACAACCATCCACTACCACCGGCGGCCATGGCGATGGCCTCAACCACAGCAGCGGCCGCTAGC
ATGCTCCTCTCGGGATCAATGCCTAGTGGTGATGCACTAATGAACCCTAATTTTCTAGCAAGGACAATCC
TCCCATGCTCATCAAGCATGGCCACAATATCAGCCTCAGCCCCCTTCCCAACAGTTACCCTAGACTTGA
CCCATTCACCGAACCCTCTCCAACTCCAAAGGGCACCCCATGTACCCCAACAAACTGCCCCCTTCCAAT
TCCCCTTCCCCACACCAAATGGGCCAGTGCCCTCCAACTTTATGGCCTCATTGCCCCAAATATTTGGCCA
AGTCTTCAACAATACTACTAATAGCAACCAATCAAAATTCTCTGGCCTACATACCTCATCCTCCTCTGCC
TCCGCACAAGCGTTGGACGCCGCCCATTTGCAACTACCTAATCAAAACGCTGCGTTTGCTGACACTCTC
AGTGCTGCCACCGCCGCCATCACCTCAGACCCCAACTTCACCGCCGCACTTGCTGCCGCCATCTCGTCC
ATCATCGGTGGCGGGGCTCGGCCAGGCGAAAATAATGTTGCCAATAATGCCCCTGTGAACTCCCCTCAC
GGTGGTGGCGGTGCCACTAGTGGCAATAAAGTCAATTCAAGTAGCTAG

>*HmoWRKY31*

ATGGAAACAACAGGATGGGGACTTGCCCTTGAATCTGATCATCATCATCACCGTCATCACCATCATC
GTATTCCTCCTCCTCATCATCATCACGCTAGTCTCCTCGCCATGAAAGGTGGAGGAAGCAGTAACAATC
GGCTGTTCCCTGGTGTAGACTTCCCCATGAATCTCAACCGTCGAGATTACCATGATCATGATCATCATGA
TGATGATCAGACTGGCCATAAGGTGGTAGTGGGTGAAGTTGATTTCTTCTCTGATCAAAAGAGTGCAAA
GAGGACGTCATCACCTCATCATAGTAGTTATCATGCCAAGGAGGAGGGTTCTCCTCGAGATCACCATCA
TCATCCTGCTCCAGCTGCCCCTGATTCCACCCCTGACCTGCTCCATGTAAATACTGGCCTACATCTCCTC
ACGACAAACATGGGAAGCGATCAGTCTACTGTAGATGACAGTGCCTCTTCAGATGCAGAGGACAAGAG
AGCCAAAAATGAGTTAGCAGCACTTCAAGCCGAGCTGGAGCGAATGAATGCCGAAAACCAGAGGTTA

AAGAACATGCTATCTCAAGTGAGCCACGACTACAGTAACCTTCAAGTGCACTTTGTATCTTTGATGCAA
CAACAACAACATCAAAAGGGTGAAAGCACCCAATCACATGAGGTGATTCAAGGGAAGGGGGAGGGA
AGGAGGAACATAGTGCCGAGGCAGTTCTTAGACATGGGTCCGAGCTCAAACAATGACACCGATGAGCT
GACCTCTCAAACATTATCCGATGATGAGCAAACCCTTGATGGTTCACCCCATAACAAAGAGGGTAATGG
CTCGTACGAGCATGGCAAAGGTGGTAGTCATGGAAGAGAAGATAGCCCTGAAGACGCTGAGTGGGTA
CCTAATAAGGCTCCCAAGCTGAACCTACCAAAGGGTAATAATAGCCCTGTTGATCAAGCTGCCGCGGA
AGCTACCATGAGGAAGGCTAGAGTCTCCGTTCGAGCCCGGTCTGAGGCGGCCATGATAAGTGATGGAT
GCCAGTGGAGAAAATATGGACAGAAGATGGCTAAGGGTAACCCTTGCCCTCGAGCTTATTATCGTTGCA
CAATGGCCGTCGGTTGTCCTGTTCGCAAACAAGTTCAAAGATGTGCAGAGGACAGATCAATACTAATTA
CAACGTACGAGGGTAATCACAACCATCCACTACCACCGGCGGCCATGGCGATGGCCTCAACCACAGCA
GCGGCCGCTAGCATGCTCCTCTCCGGATCAATGCCTAGTGGTGATGCACTAATGAACCCTAATTTTCTAG
CAAGGACAATCCTCCCATGCTCATCAAGCATGGCCACAATATCAGCCTCAGCCCCCTTCCCAACAGTTA
CCCTAGACTTGACCCATTCACCGAACCCTCTCCAACTCCAAAGGGCACCCCATGTACCCCAACAAACT
GCCCCCTTCCAATTCCCCTTCCCCACACCAAATGGGCCAGTGCCCTCCAACTTTTTGGCCTCATTGCCC
CAAATATTTGGCCAAGTCTTCAACAATACTACTAATAGCAACCAATCAAAATTCTCTGGCCTACATACCT
CATCCTCCTCTGCCTCCGCACAAGCGTTGGACGCCGCCCATTTGCAACTACCTAATCAAAACGCTGCGT
TTGCTGACACTCTCAGTGCTGCCACCGCCGCCATCACCTCTGACCCCAACTTCACCGCCGCACTTGCTG
CAGCCATCTCGTCCATCATCGGTGGCGGGGCTCGGCCAGGCGAAAACAATGTTGCCAATAATGCCCCT
GTGAACTCCCCTCACGGTGGCGGCGGTGCCACTAGTGGCAATAAAGTCAATTCAAGTAGCTAG

>*HuWRKY32*

ATGGCCTCTAACCCTGGAATTGCTGTGGATACAGACCCAAAATCAAAACCCCAGGATGAAACAACCCC
AGATTCTAAAATTTCATCTCAACCCACCACCACTTCAACTTCTGATTCAAAGTCTCTAACTTTACCTTCA
ACTTCTAACAACAGTCAAGTGGGTTACCCTTCTTTCTCTAAGCTTCTGGAGGGTGTTATGGCTGCTAGT
TCGACTCTCTTAGGCAAAGATGCTAGCTTTAAGGGACAACTTAGGATGTCTCATCAAGAGGCTTTGGCA
AATGTTACTGCTAAAGCAGCTCAAACTCAAGTGCAGCAGCCTGAAGCTAAGTCTGCGAATTCCTCAAT
TGAGCCGCCACCAAGTGAGCAACTTGAATCATCTGTTCCAAGTCCATCTGCGATTGTACGCGAAGAAA
AACCATCCTTGCAACATAAAGATAATGTGCAAGAGGGAAACAATAAAAAATCTTCCTGCCCTGGCTCG
AAGCCTAGAGATTCTTCACCCTGTAAACCTCTTGCTGCGGAATTGGCTAAGGAGAAAGTTGATAAGCA
AGATTGGAAGCAGGGAAACTCCTGCTATAGTAATGAACAAGCAGATGATATCCGAAGGACACCCAACT
CAGATGGGTATGGCTGGAGAAAATATGGCCAAAAGCAGGTGAAGAATTCTGGGAGTTCTAGAAGCTAT

TATCGATGCACGTTTTATAATTGCTTTGCTAAAAAGAAGGTTCAGCATTTGGACGTATCAGGCAAGATAG

TTGATGTTGTTTATAAGGGTGACCATAACCATGACCCACCTCAAAATCTTAAGGCTTCAAGCCGGAAGA

GGGATGCACCATCAGCTGCACAGGTGAATGATCTTAGTAGCAGTTCAGTGCAAAAGCTCGGTGATCCA

CATACACCAACATCTACATGTAGGAAGGAGGTTCAGCAAGAAGTACCACCACTTACTCGTGAATGTGG

GGAAATAAAACTGAGTACTCCTGCTAGCTCACCAGAAGAGAAAGATGTGGGGGAAGAAAGCATCACG

ACCCCTGATTCAACTCGAAGACTTAAAAGTGAGGAACCAACCTTGTCAGCAGAGGCGGAACATTATAA

ATCAAGTAAATCCAATGAAGTCAGCGTGAAGGAGTTCGAAGTTATCCCTTTGAAGAAAAGAAGGATAA

AAGAAAGTGGTCAAACATGTTCAGAGTCTGTGTATAGGACATTCAAGGAGCCTAAAGTAGTTGTGCAT

GCTTCTGGGGATGTGGCAAGCTCAAGTGATGGATATAGATGGCGAAAATACGGGCAGAAGATGGTCAA

AGGAAACCCAAATCCCAGGAGCTACTACAGGTGTTCATCAGCCGGGTGCCCGGTTAGAAAGCATGTCG

AGAGGGCAACCGACAATTCAACTGCGATCATAGTCACATACGAAGGCATACACGACCATGACAAGCCG

GTCCCCAAGAAGTGTCATGGCTCACCAAAATCCCCAGTCCATGCGAGTTATGCAACTGCAAACAGCAC

CGCGGTTGAGAAGTCAGAAGCCAAGTCCCCACCTCCGAGTCAAGGACCTTCAGCACCGCAATCCAGA

GATGTTGTCAACAGTGATGCCAATGGAGATAAGCCTGGGGAAGCCGGTACTGACAAGGTGTTGGAATC

AGCTCGAACGCTGTTGAGCATTGGAATTGAGCTCAAGTCTTGCTGA

>HmoWRKY32

ATGGCCTCTAACCCTGGAATTGCTATGGATACAGACCCAAAATCAAAACCCCAGGATGAAACGACCCC

AGATTCTAAAATTTCATCTCAACCCACCACCACTTCAACTTCTGATTCAAAGTCTCTAACTTTACCTTCA

ACTTCTAACAACAGTCAAGTGGGTTACCCTTCTTTCTCTAAGCTTCTGGAGGGTGTTATGGCTGCTAGT

TCGACTCTCTTAGGCAAAGATGCTAGCTTTAAGGGACAACTTAGGATGTCTCATCAAGAGGCTTTGGCA

AATGTTACTGCTAAAGCAGCTCAAACTCAAGTGCAGCAGCCTGAAGCTAAGTCTGCGAATTCCTCAAT

TGAGCCGCCACCAAGTGAGCAACTTGAATCATCTGTTCCAAGTCCATCTGCGATTGTACGCGAAGAAA

AACCATCCTTGCAACATAAAGATAATGTACAAGAGGGAAACAATAAAAAATCTTCCTGCCCTGGCTCGA

AGCCTAGAGATTCTTCACCCTGTAAACCTCTTGCTGCGGAATTGGCTAAGGAGAAAGTTGATAAGCAA

GATTGGAAGCAGGGAAACTCCTGCTATAGTAATGAACAAGCAGATGATATCCGAAGGACACCCAACTC

AGATGGGTATGGCTGGAGAAAATATGGCCAAAAGCAGGTGAAGAATTCTGGGAGTTCTAGAAGCTATT

ATCGATGCACGTTTTATAATTGCTTTGCTAAAAAGAAGGTTCAGCATTTGGACGTATCAGGCAAGATAGT

TGATGTTGTTTATAAGGGTGACCATAACCATGACCCACCTCAAAATCTTAAGGCTTCAAGCCGGAAGAG

GGATGCACCATCAGCTGCACAGGTGAATGATCTTAGTAGCAGTTCAGTGCAAAAGCTCGGTGATCCAC

ATACACCAACATCTACATGTAGGAAGGAGGTTCAGCAAGAAGTACCACCACATACTCGTGAATGTGGG

GAAATAAAACTGAGTACTCCTGCTAGCTCACCAGAAGAGAAAGATGTGGGGGAAGAAAGCATCACGA

CACCTGATTCAACACGAAGACTTAAAAGTGAGGAACCAACCTTGTCAGCAGAGGCGGAACATTATAAA

TCAAGTAAATCCGATGAAGTCAGCGTGAAGGAGTTCGAAGTTATCCCTTTGAAGAAAAGAAGGATAAA

AGAAAGTGGTCAAACATGTTCAGAGTCTGTGTATAGGACATTCAAGGAGCCTAAAGTAGTTGTGCATG

CTTCTGGGGATGTGGCAAGCTCAAGTGATGGATATAGATGGCGAAAATACGGGCAGAAGATGGTCAAA

GGAAACCCAAATCCCAGGAGCTACTACAGGTGTTCATCAGCCGGGTGCCCGGTTAGAAAGCATGTCGA

GAGGGCAACCGACAATTCAACTGCGATCATAGTCACATACGAAGGCATACACGACCATGACAAGCCGG

TCCCCAAGAAGTGTCATGGCTCACCAAAATCCCCAGCCCATGCGAGTTATGCGACTGCAACCAGCACC

GCGGTTGAGAAGTCAGAAGCCAAGTCCCCACCTCTGAGTCAAGGACCTTCAGCACCGCAATCCAGAG

ATGTTGTCAACAGTGATGCCAATGGAGATAAGCCTGGGGAAGCCGGTACTGACAAGGTGTTGGAATCA

GCTCGAACGCTGTTGAGCATTGGAATTGAGCTCAAGTCTTGCTGA

>*HuWRKY33*

ATGTCTTCCTCAGTGGCAAGCATAGAGAGCAACAGCTTCTCCTTCACAGGCCTCATGGCGTCCTCCCCC

TTCACTGATTTCTTTGCTCATAATGATGATGAAACCTTGCCAAATGCGTACCAAAACCACGAAATTACTA

ATAACAATACGACCCACGACAACCATAATAGCTGCTGGGGAGGGTTCGAACAGCTGGTTGAAGTGCCC

AAGTTCAAGTCTTTAGCCCCTGCCTCTCTCTCTGTCTATCATCATCATCATGATGATGATCATCATCAATC

TGCTGCTCCTGTTTCTCCCTCTTCCTTCTTGACCACTCCTCATTCTTTTAGCCCTTCCATCTTCTTGGATT

CCCCCCTCCGTAATAGTTTTCCATCAACAGTGGAGGCATTTGGTGGTGAGAGATTCAATTATAGTGGCTC

CAATGGTGAGGCCCAACAGGGTACCGATGATGAAGAAAGAAGCAACAACAATTTCTCTTTCCATGCTC

CAAACATCTCAACGGAAGGATGGAATTTAGATGCACCCAAGAAGCAAACTGATCTGAATTTTAACAAA

GAGCAACCCGGTTCGAAACCGGGGTTCATCCAAAATCAAATAGTACCCCCAAAAATAGCCACGGCACA

ACCAGAGGCTACTATGCAGAGAAGTTACAACCATTCTCAGACTCAGCCTGCTGTGCAGATGAACAGAG

CCCAGAAAAGATCGGAGGACGGGTACTGCTGGAGGAAATACGGGCAAAAACAGGTCAAGGGGAGCG

AAAATCCCCGGAGTTATTACAAGTGCTCGTACCCGAATTGCCCTACAAAGAAGAAGGTGGAGAGGTCT

CCTGAAGGGTATGTCACTGAGATTGTTTATAAGGGGAGTCACAACCACCCCAAGCCTCAGCCCGGCAG

AAGATCATCAGTTCAGAACAATGCATTTGATGGTTCAGAGGGTTCTAATGCATCAGGTGCTCACGCGCA

GACTGAGTACTCTACTTTCACCCCTGAGACATCGTCCGTTTCTCTAGATGACGATGATGAATTTGATCAG

ACTTCTGCTATGAGCAGATCAGGCAAGGATGACGAGAATGAACATGAAGCCAAAAGATGGAAGGGCG

ACAATGAAGGGGAGGTCATATCAGGCTATGGAAGCAAAACTGTCAGAGAACCAAGAGTCATAGTTCAA

ACAACCAGCGAGATTGATATTCTTGATGATGGGTATAGATGGAGAAAATATGGGCAGAAAGTAGTGAAG

GGCAACCCTAATCCTAGGAGTTACTACAAATGCACAAGCTCCGGGTGCCCTGTAAGGAAGCACATAGA

GCGGGCATCCCATGACTTGAAGGCTGTTATCACAACCTATGAAGGGAAACACAACCATGATATTCCTGC

TGCTCGTGGTAGCAGCACCTATAACGCCCCCATTCCAGTTAGGCCATCCGCCATTAGCAGCCGACAAAA

CCAGCCAGTCAGCGTGAAGCATCTCAGTAACAGTACCAGCACGTCTGGACCAGAAGCTCAATCACCCT

TCAGCTTACAGATGCTGCAAGGACATGGTAACTTCGGGTTCTCAGCGTTTGGGAATCCATCTGGAGCCA

AGGATGAGCCAATGGACAACACATTTTTAGACTCATTTCTTCATTGA

>*HmoWRKY33*

ATGTCTTCCTCAGTGGCAAGCATAGAGAGCAACAGCTTCTCCTTCACAGGCCTCATGGCGTCCTCCCCC

TTCACTGATTTCTTTGCTCATAATGATGATGAAACCTTGCCAAATGCGTACCAAAACCACGAAATTACTA

ATAACAATACGACCCACGACAACCATAATAGCTGCTGGGGAGGGTTCGAACAGCTGGTTGAAGTGCCC

AAGTTCAAGTCTTTAGCCCCTGCCTCTCTCTCTGTCTATCATCATCATCATGATGATGATCATCATCAATC

TGCTGCTCCTGTTTCTCCCTCTTCCTTCTTGACCACTCCTCATTCTTTTAGCCCTTCCATCTTCTTGGATT

CCCCCCTCCGTAATAGTTTTCCATCAACAGTGGAGGCATTTGGTGGTGAGAGATTCAATTATAGTGGCTC

CAATGGTGAGGCCCAACAGGGTACCGATGATGAAGAAAGAAGCAACAACAATTTCTCTTTCCATGCTC

CAAACATCTCAACGGAAGGATGGAATTTAGATGCACCCAAGAAGCAAACTGATCTGAATTTTAACAAA

GAGCAACCCGGTTCGAAACCGGGGTTCATCCAAAATCAAATAGTACCCCCAAAAATAGCCACGGCACA

ACCAGAGGCTACTATGCAGAGAAGTTACAACCATTCTCAGACTCAGCCTGCTGTGCAGATGAACAGAG

CCCAGAAAAGATCGGAGGACGGGTACTGCTGGAGGAAATACGGGCAAAAACAGGTCAAGGGGAGCG

AAAATCCCCGGAGTTATTACAAGTGCTCGTACCCGAATTGCCCTACAAAGAAGAAGGTGGAGAGGTCT

CCTGAAGGGTATGTCACTGAGATTGTTTATAAGGGGAGTCACAACCACCCCAAGCCTCAGCCCGGCAG

AAGATCATCAGTTCAGAACAATGCATTTGATGGTTCAGAGGGTTCTAATGCATCAGGTGCTCACGCGCA

GACTGAGTACTCTACTTTCACCCCTGAGACATCGTCCGTTTCTCTAGATGACGATGATGAATTTGATCAG

ACTTCTGCTATGAGCAGATCAGGCAAGGATGACGAGAATGAACATGAAGCCAAAAGATGGAAGGGCG

ACAATGAAGGGGAGGTCATATCAGGCTATGGAAGCAAAACTGTCAGAGAACCAAGAGTCATAGTTCAA

ACAACCAGCGAGATTGATATTCTTGATGATGGGTATAGATGGAGAAAATATGGGCAGAAAGTAGTGAAG

GGCAACCCTAATCCTAGGAGTTACTACAAATGCACAAGCTCCGGGTGCCCTGTAAGGAAGCACATAGA

GCGGGCATCCCATGACTTGAAGGCTGTTATCACAACCTATGAAGGGAAACACAACCATGATATTCCTGC

TGCTCGTGGTAGCAGCACCTATAACGCCCCCATTCCAGTTAGGCCATCCGCCATTAGCAGCCGACAAAA

CCAGCCAGTCAGCGTGAAGCATCTCAGTAACAGTACCAGCACGTCTGGACCAGAAGCTCAATCACCCT

TCAGCTTACAGATGCTGCAAGGACATGGTAACTTCGGGTTCTCAGCGTTTGGGAATCCATCTGGAGCCA

AGGATGAGCCAATGGACAACACATTTTTAGACTCATTTCTTCATTGA

>*HuWRKY34*

ATGTTCCCCTCTTCCATGCCCTCTTTTCCAACTGGAGGGCAGAACCAGCAGCAACATCAACAATCATCA

TGGAGTACAGGAGAGCAGAAGCAACAGGATGGTCATTATCAACCATCCAAATTGGTAAATTCCCAGGG

TCAGACACAGGGTCAATCTGAAAATTATAATATCAAATATGAGGCGGATCAGAGTTCTTCTTACCAAGC

ATCTGATTATGCAAGAGAAAGAGAGCAAAGTAGTAGAAAGTCAGATGATGGGTACAACTGGAGAAAAT

ATGGGCAAAAACAGGTAAAAGGGAGTGAAAACCCTCGGAGTTATTACAAGTGTACATATCCCAATTGT

CCCATGAAGAAGAAGATTGAGACGGCTCTTGATGGCCGAATCACTGAGATTGTTTATAAAGGTAGCCAT

AACCATCCGAAGCCAGTTAATAACCGAAAATCAGCCTTAGCCTCAGCTTCATCATCCTTTCAAGCCTTG

CTAATTCCTCCTTATAATGATTACCAAGATCACTCTTCTTCGGGGGTGTTGCCACAGGGATCATCTGGGC

AAACTGACTCGGTTGCTACCCCAGAGAACTCCTCGATTTCGATTGTTGATGATGAGCAGGACATGGGTT

CTCAGAGGAGTAGATCAGCTGGATCTGGGGACAATGATTTTGATGATGATGATGTTAATAATGAACCTAA

TGCCAAAAGATGGAAAGGGGAAATTGGTGAAAATGAATGCATCTCAACAGCTGTTCTTGGGGCTGGGA

CTGTTAGTGGGAACAGAACAGTCAGAGAACCAAGAGTGGTTGTTCAGACCACAAGTGACATCGATATT

CTGGATGATGGATATAGATGGAGAAAATATGGTCAGAAAGTTGTCAAGGGCAATCCTAATCCAAGGAGC

TACTATAAGTGCACTCATCCGGGTTGCCCAGTGAGGAAACACGTGGAACGGGCTGCTCAAGATCTAAG

GTCAGTGATCACCACTTATGAAGGCAAGCACAACCATGATGTTCCTGCTGCTCGAGGCAGTGGAGTCA

ACAGGGCCAACAACATCCATGGCATGGGCACTGGGGCAGGGGTGGTTCCATCCATGGCCGTAAGGCCA

TCTGCGTTAAATTATATGGGCTCGATCAACCCTAACCCTGACTCTAACCTTAACCTCAACGCTCGGATGC

CCACTTCAAACTACGCAGTTCGAGGTCCCCAAGGCCAAGGGACCGTGCAGTTACCCCCCTTTGCATTA

GAGATGCTGCAGAATAACCCTGGGAGTTTTGGCTTCTCATCAGGGTTAGGATTGATGAATCAAGTTCAA

GCCAAGGAAGAACCAAAAGATCATGACATGTATTTTGATTCTTTTCTGTACTGA

>*HmoWRKY34*

ATGGGGGACAGTGAAGATGCATTGGATGAGTGGAGGGATCTTGTGGTGAGTCCTAAGGGACTATTCTC

AGTCTTTTCACCTGAGCGCTGTGTTACAAAATCAATTGCTGATGTTTTCAGTGATCGGAAGGGGATTTG

GGGGGGTGCTGATGACAAAGAAATGGAGAATTCAGTGTTTGATTCGGGGAAGAAGTTGGAGGATTGT

GAGCAGGGATGGGATTCTTCGATTGTCGAGGATGATTTGGATGTGCCCATGGCAGGAGGGAGCATAGT

GGATACGAGGGCTGCTAAGTTGGGGTTCAATGCTCCTAACACAAGCTCCCCTCCTTTTAGCTCTAGCAG

CCCGTTGCCCTCCCCAGGTTCTCGATCACCCTATCTTATCGCACCTGCTAGTATTAGCCCTACTGGACTG

CTTGACTCTCCACCTTTGCTGCCAAATTCACAGCCTTCTCCAACCACAGGAACTTTTGAATCACCTCCT

GAGGGTGGAAACAATCAGATGGAGATTCCAGCATCTTCTGCAGGCACGGACAAGCACAATGATGGTGG
CACTACAACCATGATTAAGGCCAATGATGCCATTCCTATCACATGTTTCTGTGCCGTAGAAAATGAGGG
GTCCCCTGGTGGTTTGGCTCAGAGTTTGGTTGCTCGTGTTCATGGTCAAAGTATTAACACAATGCCGCT
ACCAGTGAACTTCGAAATTCCAATGCAGTTTCCTAATGAAGTTGCCATGAAAAGTCATGAAATTGGCTC
ACCAAATGATGAACAGTTGTCGGATGACATCACTGTAGACTCCAACCCCACCACTCTGCAACCATGCCA
TTCGACGACTTTGGTATGTGATCAATTGCAGGAAAAGGGCGAACCGTCTCTGGAAGAGGATGCTGATA
CTTATAATGCCCTGGATTCAGAACACAAAGGAACATATGCTCCAACAGGGACAGTGAGAACCACAGAA
GATGGGTACAATTGGAGAAAATATGGACAGAAACAAGTCAAGGGTAGTGAATATCCACGCAGTTACTAT
AAATGCACCCACCCAACCTGTCAAGTGAAGAAGAAGGTAGAACGTTCTCATGATGGCCAAATTACAGA
GATTATATACAAGGGTGGCCATAACCATCCAATGCCCCAACCCTGTAGGAAATCAGCATTTAGTTCATCA
TTACCATTTCGTGAGACATCAGATATATGTGACGGTATTAGCTCTTGCGTCAAAACTGAGAGTGAATCAA
CCTGGAGAAACATGCAGCCAGGGAGTAAAGGTGGCATGCCTGGGTCTGACTGGAGGACTGATGGCTT
GGACAGGGCATCCTCTACCTCTGTTGTGACTGAAGTTTCTGATATCCTGTCCACCAATCAAGAAAAACC
CTTATGCATGCTTGAATCAGCAGGAAGTCCAGAGTTCTCATCTACAATTGCTATTAATGAGGATGATGAA
GAAGATACTACCACCCAAGGGGGTTGTTTACTTGCAGATGATGATGACAATAAATCAGACTCAAAAAG
AAGGAAAAAGGAGTCTTGTTTTTATGAAACAACTTTAGGTTCCAGGGCTGTTCGCGAACCAAGAGTTG
TTATCCAAATAGAGAGTGATATTGATATACTAGATGATGGGTATCGCTGGCGCAAGTACGGGCAGAAGG
TTGTGAAGGGGAATCCAAATCCTAGGAGCTACTACAAATGCACCCGTCCTGGATGCCCTGTCAGGAAG
CATGTGGAAAGAGCCTCACACGACTTGAAATTTGTGATAACAACGTATGAAGGAAAACACAATCATGA
AGTCCCTGCTGCTAGAAATAGCTGTCATGCCAATTC
AAGCAGTGGAGGCTTGCCCCCTCCCACATCCAGTACACAGGCTACCCTGAACTTACCGCGAAACCCTC
ATCTTCCAAAGCCGGAGTCTCATGTACAAGATCTCCCCCCTCATTTTGGCAGAAAATTGGAGTGCAATA
GTGACTTCTTAGGAGCGAACTTTCTAGGGAATTTTGGAGGCGGAGCTTCCTCATTTTATCAAATGAAGT
TTCCAGCCTTACCGAATGCTGTACCGTATGGTCCATTTAGTTTGAGTAATCACCATCCAACTTTCCCCGG
CAATGTGGTGCCAGGCTTCGCAGATCTTTCCCTCCCATTGCCACTCGGATTCCCCCATTCCTCAAATTTA
GCCATTGCCAACTATACCAATCCTGGAAGACCACTTGCTTCAGTCCAGCCCTTCATGGCAGGCCATCAC
CATGAGGAACCTAACTCCAAGATCCTTAGACCTAAGCAGGAACAGGGAGATGACTCATTTTGTGATAC
AGGAATGCATCACATCGACCATACCATGTCTTCATCATCATCTATATACAGCCAAATTGTGTGA

>*HuWRKY35*

ATGGTAATGGATAATAAACGTCGGGCTAGCTCACCGGTCACCACTTCTGATCACGACGGGCCTCAGAGC

TCATCGGAGAATGCACCTGATTCGCATCCTTCAGCTGATCGTGACCCGAGAACCACTAACATGGGCCCC

AGCCACCTTTTGGCTAAGAAAAGGAGCATAAAGAAGAGGGTGGTATCCGTGAAAATAGGAGATAAGGA

TGGAACAAGGAGCAAAGGGAAGGCGTATCCACCGTCTGATGCATGGGCTTGGAGAAAATACGGCCAG

AAACCCATCAAAGGCTCTCCTTATCCCAGGGGATATTATCGGTGCAGTAGTTTCAAGGGCTGTCCTGCT

AGAAAACAAGTCGAGAGGAATCGGGCAGACCCCACCACACTCATCATCACCTATGCTTATGAGCACAA

CCACCCCTTGCCCACCTCCAGCCACCACCACCACGCTGCCGACACTTCAACCGCCTCCACCACTGACA

CTGCCAGTCCAACCACCCCATCCGCCACCACAACCACTGCCTCAACTGATGCTCCTGATGATTCAACTG

ACAATGGGGAGCCGGAGGCAGAGCTATCATCACTTCCTGTTCAAGAAGTTATCCCCTTTCGAGATTCAA

ACCACCTTGAGTTCATCGGAGACTCATCCATACTAGGCTGCTTAGGGGTGGAATACACTTGGGTGTCTG

ACATGCCATTCGAGATGCCATCCCTTGTGTGGCCCAATAGTGGGGATGCTGATGTGTTGGCATACTCAAT

TCGGGAGGAAGATGAACTACTGTTTGGGGACCTAGACGAGCTCCCAGAATGCTCCATTGTCTTTCGAA

AGAGAAATGGAGGCATCAAAAGGTGTCGCACAACTCCCATATATGGCAACACGTAG

>*HmoWRKY35*

ATGGTAATGGATAATAAACGTCGGGCTAGCTCACCGGTCACCACTTCTGATCACGACGGGCCTCAGAGC

TCATCGGAGAATGCACCTGATTCGCATCCTTCAGCTGATCGTGACCCGAGAACCACTAACATGGGCCCC

AGCCACCTTTTGGCTAAGAAAAGGAGCATAAAGAAGAGGGTGGTATCCGTGAAAATAGGAGATAAGGA

TGGAACAAGGAGCAAAGGGAAGGCGTATCCACCGTCTGATGCATGGGCTTGGAGAAAATACGGCCAG

AAACCCATCAAAGGCTCTCCTTATCCCAGGGGATATTATCGGTGCAGTAGTTTCAAGGGCTGTCCTGCT

AGAAAACAAGTCGAGAGGAATCGGGCAGACCCCACCACACTCATCATCACCTATGCTTATGAGCACAA

CCACCCCTTGCCCACCTCCAGCCACCACCACCACGCTGCCGACACTTCAACCGCCTCCACCACTGACA

CTGCCAGTCCAACCACCCCATCCGCCACCACAACCACTGCCTCAACTGATGCTCCTGATG

ATTCAACTGACAATGGGGAGCCGGAGGCAGAGCTATCATCACTTCCTGTTCAAGAAGTTATCCCCTTTC

GAGATTCAAACCACCTTGAGTTCATCGGAGACTCATCCATACTAGGCTGCTTAGGGGTGGAATACACTT

GGGTGTCTGACATGCCATTCGAGATGCCATCCCTTGTGTGGCCCAATAGTGGGGATGCTGATGTGTTGG

CATACTCAATTCGGGAGGAAGATGAACTACTGTTTGGGGACCTAGACGAGCTCCCAGAATGCTCCATTG

TCTTTCGAAAGAGAAATGGAGGCATCAAAAGGTGTCGCACAACTCCCATATATGGCAACACGTAG

>*HuWRKY36*

ATGGGTGAAGTAAGGGAGGAAAATGAGAGGTTGAAGAAGTTGTTAGTGAAAATTGTCAAGGACTATG

AGTCCTTAGAACAGAAAGTTTCTTCGATAACTCGATTTCAACAGTCGGTAGAATCAAATCTAAGTAAGA

ATCTCCCTGACAACGATAATGAACATGATAGTCAAGTGAATCCCGAGTTGGTTTCTTTAAGCCTAGGGA

GCTGGTCTTCGAAAAAGAAGAAACATGATGATATAAAGCAACAGGTCAGTGATGCTTGGAGTAAAACG
AGAAAGGTGGATGGAGGGCTTGAGCTTGGGCTCGAATGTAAATTCAAGAATTATTCCACCACTGTTGG
TGATTCGGTGAAGAATTCAACACTGGATAGCGGGAATGAAGAGATGGGGGAGGAAAACAACAAAACA
AAAGAGAATACAAGCTCTAAGGAAGGGGAGGAAGAAAGCTTGCACCCACCTCAAGTCAAGAAAACT
AGGGTTTGCATAAGATCAAGATGTGACACCCCTACGATGAATGATGGATGTCAATGGAGAAAGTATGGG
CAGAAAATTGCCAAAGGAAATCCCTGCCCTCGAGCTTACTACCGTTGTACGGTTTCGCCTTCATGCCCC
GTGAGGAAACAGGTTCAAAGATGCCCTGATGACATGTCCATCTTGATCACCACCTATGAAGGCACCCA
CAACCACCCCCTCCCTCTCTCGGCCACCACCATGGCCTCCGCCACAACTGCAATTGCCGCCATGCACA
AGTCAGACTCCTTTACCTCCTCCTCACAATCATCCTTCGTGGGCCCTACCTTGACTTCATCTTTTGGCTT
CCATGGCACAAACTTGGTCACTCCCTTCAATCATGCCCCTAGATTAAGTATCTCTAATCCTTCCATGATCT
CTGCATCTCACAATTCTCATCCTACCATTACCCTCGACCTCACCACACCGAGCACCGGCCTCTCCCCTTT
CAACTGGCTATCCTCATCTTCATCTTCTTCCCTAAGAAATTCTTCCAACTTCCTTAACTTCTCTTCCTCTT
CTGCTTCAACTATGACTTCTTTAGACTTCACTACCAAACCAGCAGGTTCTTCTTGGGGGAGTGGGTACT
TGAATTATGGGCCATCATTTCCTTACATTAATAGTAGTAGCATCAGTAATACTACTAGTAGTATAAATATTT
TTGAAAACCCTAACAATCCTCAGCCTTCTCTAGATGAGACAATAACAAAGGCAATTGCAGCAAACCCT
AATTTCCAATCAGTGTTAGCTGCTGCTATCTCATCATATGTTGGAAACAAGACGACTACTACTACTAGCA
ATAATAATAGCGAGCAAAGGAAACCATCGCCTGCTTTGTGTAATTCTCAGACAGAAGCTAATGGACGAA
GCTTATCGTTGTTTCCCACACTTGCATCCAAGAGTACTTCTCATGGAGGTGTTGATAAAAAGGACCACA
GTAGCTGA

>*HuWRKY38*

ATGTTGAAGGGTTCAATTGAACAAAGGGATCTGATGATACAGCAACTAATTGAGGGGATTGAGGCAGC
AAGGCAACTCCAAAAGAGGATTCGAAGTGGCCCATCAAAAATTGATCATCATGGTTGTGATTATGATAA
TCCTCAATTAGGGGAAGATGATCAAGCCCTTAATCACTCATTATTAGATAAGATGGTGGCTTCATTTGAT
AAGACAATCTCAATTGCAAAACTCCTCAAATTGGAGCTCCAACATCAGTCTGATCATCAACAACCCTCC
AATAATTCCCAATATGAGGGGTCACCCCCTCATTCTTTGGCTCTTAGCAGCCCTAGAAGTGAAAACTCC
CATCATCATCTTCATTTGGCCAAGAAAAGGAAAATGATGCCAAGGTGTAGCGTGAAGGTAAAAGCTGA
GGGAGGAACAGGGCTAGAAGGACCTCTCGATGATGGCTACACTTGGAGAAAATACGGACAGAAAGAT
ATCCTTGGAGCTAAGCATCCTAGAGGATATTACAGATGCACCCATCGCCACACACGAAGTTGTGGAGCC
ACAAAGCAGGTCCAACGCACAGATGAAGACCCAACCATCTTCCACGTCATCTATCGTGGCGAGCACAC

TTGTTTTCCTGTGCCCCATTTCTCCCAAGCTCAATCCAAGCCCAAGAAGCAGACCAAAGTAAAAACCG
AGCCCAAACCACAACCACCTCCAAGCCCACAAGCACAACCTCAGATGGCTTACTCGCCGGAGCCAGA
AGTCTATTTGGGCCTGGACCCAGATGGCGCCCTTAAGGTCGAACCAAACGAGACAGAAAGTCCAGCCC
AGTTTTTTAGATCCTTTTCGTTCACCCCGGCCCTGTTAGAGCCTGGGCCCATGGACAACTTCTTATCGGA
CTGCTACCCTACTTTTGGGTCGCCGGCCACCTCCGAGTCCAACTATTTTCCGGTCACCGTGCTCAACAT
ACAAACATCAGAATCGGACCTAAACGGGTCAATATCGGGTCCGAACTCGGTATCCAATTCCCCCATGGG
ATGTGACTTCGAGTTCCCCATTGATCAGCTACTAGATATTGAGGCAGATTTTCCTAATTTTGAGTTTTAA

>*HmoWRKY38*

ATGTTGAAGGGTTCAATTGAACAAAGGGATCTGATGATACAGCAACTAATTGAGGGGATTGAGGCAGC
AAGGCAACTCCAAAAGAGGATTCGAAGTGGCCCATCAAAAATTGATCATCATGGTTGTGATTATGATAA
TCCTCAATTAGGGGAAGATGATCAAGCCCTTAATCACTCATTATTAGATAAGATGGTGGCTTCATTTGAT
AAGACAATCTCAATTGCAAAACTCCTCAAATTGGAGCTCCAACATCAGTCTGATCATCAACAACCCTCC
AATAATTCCCAATATGAGGGGTCACCCCCTCATTCTTTGGCTCTTAGCAGCCCTAGAAGTGAAAACTCC
CATCATCATCTTCATTTGGCCAAGAAAAGGAAAATGATGCCAAGGTGTAGCGTGAAGGTAAAAGCTGA
GGGAGGAACAGGGCTAGAAGGACCTCTCGATGATGGCTACACTTGGAGAAAATACGGACAGAAAGAT
ATCCTTGGAGCTAAGCATCCTAGAGGATATTACAGATGCACCCATCGCCACACACGAAGTTGTGGAGCC
ACAAAGCAGGTCCAACGCACAGATGAAGACCCAACCATCTTCCACGTCATCTATCGTGGCGAGCACAC
TTGTTTTCCTGTGCCCCATTTCTCCCAAGCTCAATCCAAGCCCAAGAAGCAGACCAAAGTAAAAACCG
AGCCCAAACCACAACCACCTCCAAGCCCACAAGCACAACCTCAGATGGCTTACTCGCCGGAGCCAGA
AGTCTATTTGGGCCTGGACCCAGATGGCGCCCTTAAGGTCGAACCAAACGAGACAGAAAGTCCAGCCC
AGTTTTTTAGATCCTTTTCGTTCACCCCGGCCCTGTTAGAGCCTGGGCCCATGGACAACTTCTTATCGGA
CTGCTACCCTACTTTTGGGTCGCCGGCCACCTCCGAGTCCAACTATTTTCCGGTCACCGTGCTCAACAT
ACAAACATCAGAATCGGACCTAAACGGGTCAATATCGGGTCCGAACTCGGTATCCAATTCCCCCGTGGG
ATGTGACTTCGAGTTCCCCATTGATCAGCTACTAGATATTGAGGCAGATTTTCCTAATTTTGAGTTTTAA

>*HuWRKY39*

ATGGAGGGCCTTGAGGAAGTTAACAAAGCTGCTGTGGCGAGTTGTCATAGAGTTTTGAGTATCATATCT
CAACCTCAAGAGCATACCCGGTTTAGGAGTTTAGCTGTTGAGACTGAACAAGCTGTGACTAAGATAAA
GACAGTTGTCTCTCTACTTAGTAATGGTAATGGATTAGGTCATGCAAGGGCTAGGATTGTGAAGAGAAT
TAGGAACAACCCTTTTCCTCAAAGTATTTTCTTAGAGAGCCCAAGTAACCCTAAGGCTGATCAGAATCA
ACCGGTAAAGGCTATTCAGCTGCTCAAAACCAACTTGAATGAGTGCCATGTTCAAGAGAATGGCTCTG

CAACTGGCAAAAGTTGCTTGTCTTTGGGGGGAGATTTGTCACTGGAAATGAGCTCGAGTGCAAAAACT
CCTCTACAACTTGCTCATCCCACTACAAAAGCACATTATCAGTTTCTTCAGCAACAGCAACTACAGCAG
CAGAAGCTTCAACAGGAGTTAAAACTCCGAGCTGAGATGTTTCATCGAATGAGCAACAGCACGATTAG
TCTCAATTTTGATAGCTCTAGTTGCACCCCGACAATGTCATCGACTCGGTCTTTCATTTCTTCAGTGAGC
ATTGATGGAAGTGTGGCTAATTTGGATGGAAATGGGTTCAATTTGATTGGAGGGAGTCCCTCATTGGAC
CAGAACTTGAGTCAAAGCAGGAAGAGGTGCTTGGGGAGAGGCGAAGAGGGAAGTCTTAAATGCAGT
AGCAGTGGAAGATGTCATTGCTCAAAAAAGAGGAAGCATAGGGTGAAGAGATCAATCAAGGTTCCTG
CTGTCAGTAATAAGCTGGCTGATATCCCTCCAGACGAGTGCTCGTGGAGAAAATACGGGCAGAAGCCA
ATCAAGGGTTCGCCTCACCCTAGGGGTTACTATAAGTGTAGCAGCGTGAGAGGCTGCCCAGCAAGGAA
GCATGTTGAGAGATGTGTGGAAGATCCTTCAATGCTTATTGTGACATATGAAGGTGAGCACAATCACGC
TAGCGTACCTTCACAATCCACAAACTCGTGA

>*HmoWRKY39*

ATGGAGGGCCTTGAGGAAGTTAACAAAGCTGCTGTGGCGAGTTGTCATAGAGTTTTGAGTATCATATCT
CAACCTCAAGAGCATACCCGGTTTAGGAGTTTAGCTGTTGAGACTGAACAAGCTGTGACTAAGATAAA
GACAGTTGTCTCTCTACTTAGTAATGGTAATGGATTAGGTCATGCAAGGGCTAGGATTGTGAAGAGAAT
TAGGAACAACCCTTTTCCTCAAAGTATTTTCTTAGAGAGCCCAAGTAACCCTAAGGCTGATCAGAATCA
ACCGGTAAAGGCTATTCAGCTGCTCAAAACCAACTTGAATGAGTGCCATGTTCAAGAGAATGGCTCTG
CAACTGGCAAAAGTTGCTTGTCTTTGGGGGGAGATTTGTCACTGGAAATGAGCTCGAGTGCAAAAACT
CCTCTACAACTTGCTCATCCCACTACAAAAGCACATTATCAGTTTCTTCAGCAACAGCAACTACAGCAG
CAGAAGCTTCAACAGGAGTTAAAACTCCGAGCTGAGATGTTTCATCGAATGAGCAACAGCACGATTAG
TCTCAATTTTGATAGCTCTAGTTGCACCCCGACAATGTCATCGACTCGGTCTTTCATTTCTTCAGTGAGC
ATTGATGGAAGTGTGGCTAATTTGGATGGAAATGGGTTCAATTTGATTGGAGGGAGTCCCTCATTGGAC
CAGAACTTGAGTCAAAGCAGGAAGAGGTGCTTGGGGAGAGGCGAAGAGGGAAGTCTTAAATGCAGT
AGCAGTGGAAGATGTCATTGCTCAAAAAAGAGGAAGCATAGGGTGAAGAGATCAATCAAGGTTCCTG
CTGTCAGTAATAAGCTGGCTGATATCCCTCCAGACGAGTGCTCGTGGAGAAAATACGGGCAGAAGCCA
ATCAAGGGTTCGCCTCACCCTAGGGGTTACTATAAGTGTAGCAGCGTGAGAGGCTGCCCAGCAAGGAA
GCATGTTGAGAGATGTGTGGAAGATCCTTCAATGCTTATTGTGACATATGAAGGTGAGCACAATCACGC
TAGCGTACCTTCACAATCCACAAACTCGTGA

>*HuWRKY40*

ATGGATTATTCAACGTGGTTAAGCTCAGCTTCATTGGATCTGAGTCGTAGCAATGATCACAATCATGGCA

ATGACAATACGCTCCGGCTTTTCGATGATTCTCCTGTTCGGGATGGTCTTAAGACTGCCATCAAAGTTGA
GACGATCCAAACCTGCAGCTCCAATGAAGTTGATCGCCAACCTTTGATCAAAGATGAGGCAGGAGTTC
TGATGGAAGAGTTGAAGAAGGCAAACGAAGAGAACAGGAGGTTAACCGAAATGTTAACGGTCGTATG
TGACAATTACAATGATTTGAAGAGGCAGTTGGCGGATTACATGACTAAAGCTGCAAGTAATGAGTTGGT
GGGCAATGTGGCCAAGAAAAGAAAGATCGAGAATTCCACCAATAGTAATAACAACAACAATAACAACA
ACAGTAACAAGATTATTGGCAGTAATAATGTCGACAGTGGTTCGAGCGATGAAGAAGATTCGTGTGAG
AAACCAAAGGAAGAGGCTATTAAAGCCAAGGTCACCAGAGTTGCTGTTCGAACTGAAGCTTCTGACT
CCACCCTTATCCTGAAGGATGGATATCAATGGAGGAAATACGGGCAAAAGGTGACTAGGGATAACCCTT
GCCCTAGAGCTTACTTCAAGTGCTCCTTCGCGCCTAGTTGTCCGGTTAAAAAGAAGGTTCAAAGAAGT
CTAGAAGACCAATCAATGCTAGTTGCAACCTACGAAGGGGAGCACAACCATGCACCTCCATCCCAACA
GGAGCCAGCACTGGGCCCAAACCGGTCCTTCAGCCTCGGCTCCGTCGCATGTAACGCGACCCTAGCCT
CATCCGGACCCACAGTCACTCTTGACCTAACCAAGCCCAAGCCTAGCACTGCTAAAAATGACACAACT
CCAAAAACATTAAGCAGCAATAACGTCAGTTCTGCCCAACTGTTGAAATTCAACTCCCCTGAATTTCAG
AAGCTTTTGGCAGAACAAATGGCTTCTTCTTTGACAAAAGATCCCAACTTCACAGCTGCTCTTGCTGCT
GCCATTTCTGGGAGATCTGCACAAACCTCAACTGATTAA

>*HmoWRKY40*

ATGGATTATTCAACGTGGTTAAGCTCAGCTTCATTGGATCTGAGTCGTAGCAATGATCACAATCATGGCA
ATGACAATACGCTCCGGCTTTTCGATGATTCTCCTGTTCGGGATGGTCTTAAGACTGCCATCAAAGTTGA
GACGATCCAAACCTGCAGCTCCAATGAAGTTGATCGCCAACCTTTGATCAAAGATGAGGCAGGAGTTC
TGATGGAAGAGTTGAAGAAGGCAAACGAAGAGAACAGGAGGTTAACCGAAATGTTAACGGTCGTATG
TGACAATTACAATGATTTGAAGAGGCAGTTGGCGGATTACATGACTAAAGCTGCAAGTAATGAGTTGGT
GGGCAATGTGGCCAAGAAAAGAAAGATCGAGAATTCCACCAATAGTAATAACAACAACAATAACAACA
ACAGTAACAAGATTATTGGCAGTAATAATGTCGACAGTGGTTCGAGCGATGAAGAAGATTCGTGTGAG
AAACCAAAGGAAGAGGCTATTAAAGCCAAGGTCACCAGAGTTGCTGTTCGAACTGAAGCTTCTGACT
CCACCCTTATCCTGAAGGATGGATATCAATGGAGGAAATACGGGCAAAAGGTGACTAGGGATAACCCTT
GCCCTAGAGCTTACTTCAAGTGCTCCTTCGCGCCTAGTTGTCCGGTTAAAAAGAAGGTTCAAAGAAGT
CTAGAAGACCAATCAATGCTAGTTGCAACCTACGAAGGGGAGCACAACCATGCACCTCCATCCCAACA
GGAGCCAGCACTGGGCCCAAACCGGTCCTTCAGCCTCGGCTCCGTCGCATGTAACGCGACCCTAGCCT
CATCCGGACCCACAGTCACTCTTGACCTAACCAAGCCCAAGCCTAGCACTGCTAAAAATGACACAACT
CCAAAAACATTAAGCAGCAATAACGTCAGTTCTGCCCAACTGTTGAAATTCAACTCCCCTGAATTTCAG

AAGCTTTTGGCAGAACAAATGGCTTCTTCTTTGACAAAAGATCCCAACTTCACAGCTGCTCTTGCTGCT
GCCATTTCTGGGAGATCTGCACAAACCTCAACTGATTAA

>*HuWRKY41*

ATGGAGTTCAATGGCAACAGTAGTAGCTGTGAGCAAGGCTCCTTGGCCAGGGAGCTAATCAAGGGAAT
GGAGCTAACTAGACAGCTAAAAGCTCATATGGGTCTAGAAGTTTCTGATGAAGCTAAAGGAATACTAGT
GGAAGCGATACTGTCTTCCTATGAGAAGGCCCTTTCACTTCTGAACTTGGGTGGATCAATGGAACAGTC
TCAGCAGCATGTGGTTCCATCCACGAATGTGCCTGGATCACCCATTTCCGTCATTGGAAGTCCTAAGAG
CGAGGATCAGAACCCGGGTTTTAATGCTCAACAGGAAGCTATGGATTTCCCAAGGAAGAGAAAGATGA
TGACAACTTGGACAGAGCAGGTGAAAGCGAGCCCAGACAACAGGCTGGAAGCACCTCCTGATGACG
GTTATAGTTGGAGAAAGTACGGCCAGAAGGATATCTTGGGAGCCAAACATCCAAGAAGCTATTACAGAT
GCACATACCGCAACCTTCAAGGATGTTGTGCGATCAAGCAAGTGCAGCGAAATGATGAAGACCCCTCA
GTATTTGACATCACCTACAAAGGCAGGCATACTTGTACACCAGTTCAGCGTTCCAAGTCTGCACCAACA
TCACCAGAAAAACATGAGGGGAACAAACATCATGATCATCCCATGATGCAGCAACAGCCGAATGTGCT
GCTGAATTTTCAGAGAGGACTAAGTGTTGACATACAAAACGTAGAAAGTAAAGGAGCAGGGTATCCCC
TCTCCACAAGGGAATATGGGTACGAAAACCATACATTTACACCATCAATAATGAATGACAATAACTTCTT
TAGCAATTATTCACCATCCTTCATACCATCACCAGCCAACTCCACATCAAATTACTTCTCGAGATCACCC
TGCAATGTGAGCACCTTTGGAGGAGTTCACAGTGGACAACATTATGAATCGGACATTGCAGAGATGATT
TCAGCTGCTTCTTCTGGAACCAATTACCCCATCATTGGCATAGATTCCCCACTTCAGCAATTGGGAATCA
ACCCCTGTTTCCCATTTGATAATCAAGTGTTTTTCCCCAGCTTCTCTCAGCCTAAGGAATAG

>*HmoWRKY41*

ATGGAGTTCAATGGCAACAGTAGTAGCTGTGAGCAAGGCTCCTTGGCCAGGGAGCTAATCAAGGGAAT
GGAGCTAACTAGACAGCTAAAAGCTCATATGGGTCTAGAAGTTTCTGATGAAGCTAAAGGAATACTAGT
GGAAGCGATACTGTCTTCCTATGAGAAGGCCCTTTCACTTCTGAACTTGGGTGGATCAATGGAACAGTC
TCAGCAGCATGTGGTTCCATCCACGAATGTGCCTGGATCACCCATTTCCGTCATTGGAAGTCCTAAGAG
CGAGGATCAGAACCCGGGTTTTAATGCTCAACAGGAAGCTATGGATTTCCCAAGGAAGAGAAAGATGA
TGACAACTTGGACAGAGCAGGTGAAAGCGAGCCCAGACAACGGGCTGGAAGCACCTCCTGATGACG
GTTATAGTTGGAGAAAGTACGGCCAGAAGGATATCTTGGGAGCCAAACATCCAAGAAGCTATTACAGAT
GCACATACCGCAACCTTCAAGGATGTTGTGCGATCAAGCAAGTGCAGCGAAATGATGAAGACCCCTCA
GTATTTGACATCACCTACAAAGGCAGGCATACTTGTACACCAGTTCAGCGTTCCAAGTCTGCACCAACA
TCACCAGAAAAACATGAGGGGAACAAACATCATGATCATCCCATGATGCAGCAACAGCCGAATGTGCT

GCTGAATTTTCAGAGAGGACTAAGTGTTGACATACAAAACGTAGAAAGTAAAGGAGCAGGGTATCCCC
TCTCCACAAGGGAATATGGGTACGAAAACCATACATTTACACCATCAATAATGAATGACAATAACTTCTT
TAGCAATTATTCACCATCCTTCATACCATCACCAGCCAACTCCACATCAAATTACTTCTCGAGATCACCC
TGCAATGTGAGCACCTTTGGAGGAGTTCACAGTGGACAACATTATGAATCGGACATTGCAGAGATGATT
TCAGCTGCTTCTTCTGGAACCAATTACCCCATCATTGGCATAGATTCCCCACTTCAGCAATTGGGAATCA
ACCCCTGTTTCCCATTTGATAATCAAGTGTTTTTCCCCAGCTTCTCTCAGCCTAAGGAATAG

>*HuWRKY42*

ATGGAGATTGAGATTTTGGAAGAACCTAGAATTTTTCCAATGGAACATGAAGAAAATCAAAAGCATGT
GCTCAAGGAAATGGACTTCTTCCAAGGCTCTACTTGTTCTAAGGAGGTTCATACCAAACATGGCGATCG
AGATGGAGATGATGAACAAATCGATACTGGGTTGAATCTTCACATGAGTGGAAATATGGATGCTCTTCA
GAAGTCTACGATTGAAGACGAAGTATCATGGAAAGCATCGGAGGATAATAATCAAATTACCAAGAACA
AGTTGGTAGCCCTAAGGGACGAGATAGCAAGGAAAAACGTGGAAAATCAAAGATTGAAGACTCTACTT
AACCAAGTGATTAGCAGCTACCAAGCCCTGCAAAAGCAAATCTCCACAATTAGACAGCATCAACTTGA
TCAAAACAAAGACAAAGGAGCCCTCCAAACACAGATAGTGAAGGATGACGAGATGACCCAAAAACG
CAAAATGAGACAAGTCTTGAATCTTGGGCAAGCTACCATCAATACGAATGGAGATAATCAAGAGGAGC
TTAACCCTAATTTTCTTGATCATGACAATTACAGTAATGACGAGCACCTAAATAAGAGTGGCGCAAGAG
ATGATCATAGTTCAAACATGGCTAATAAGGAGGCCATGTTATTAGCAGCAAAAAAATCAATATCGAGCA
ATAATACTAACATGGCAAGAGTTGGCATGGGAGCTTCAATGCCAAAGGCAGAGCAGAGCCACGAAGTA
ACGATGAGACAAGCTCGTGTGTCAGTCCGAGCACGATCTGAAGCATCTATGATAAGCGATGGATGCCAA
TGGAGAAAGTATGGGCAAAAAATGGCCAAAGGAAATCCATTCCCTCGAGCTTACTATCGTTGCACCATC
GGCACTTCTTGTCCCGTCCGCAAACAGGTTCAAAGATGTGCGGAAGATAGAAGTATTCTCATAACAACT
TACGAAGGGCAACACAACCATCCATTGCCAACAGCGGCTATGACCATGGCCGCCACCACATCAGCTGC
TGCCTCAATGCTCGTGACGGGCTCAATGTCCAACCAGGTGGATGGGTCAATGAACCCGCCCGTCTTCTC
CCAGACCGCAACCCTCCCGGGCCTACCGAGCCTCGCCACGATCTCAGCCTCAGCCCCATTCCCAACTAT
CACATTGGACCTCACCAGGCCACAGCCACAGGGCAACCCTAACAATAATGGGCTGGGCCTGGTGAGCC
CGCTACAGCAGATCTTCAGTGGGCCCAACAAAGAGAATGGTAATAGCAATCTGCCTTATTTGGGGGAG
CCGGTGAGTGTTGCTAGAGCAGCCCTGGCTGCTGACCCCACCCTTGCTGCAGCCCTAGCAACTGCTATT
TCTTCCATGATTAGTAATAATAAGGGGAACAATGTCAATGTAGAATCCCATAGTAACAATGGTGGTGAAA
GGGTTGTGAGTGGAAAAAATGCCAAGGATAATCTCAACTTGTCAATTCTGTAG

>*HmoWRKY42*

ATGGAGATTGAGATTTTGGAAGAACCTAGAATTTTTCCAATGGAACATGAAGAAAATCAAAAGCATGT

GCTCAAGGAATTGGACTTCTTCCAAGGCTCTACTTGTTCTAAGGAGGTTCATACCAAACATGGCGATCG

AGATGGAGATGATGAACAAATCGATACTGGGTTGAATCTTCACATGAGTGGAAATATGGATGTTCTTCA

GAAGTCTACGATTGAAGACGAAGTATCATGGAAAGCATCGGAGGATAATAATCAAATTACCAAGAACA

AGTTGGTAGCCCTAAGGGACGAGATAGCAAGGAAAAGCGTGGAAAATCAAAGATTGAAGACTTTACTT

AACCAAGTGATTAGCAGCTACCAAGCCCTGCAAAAGCAAATCTCCACAATTAGACAGCATCAACTTGA

TCAAAACAAAGACAAAGGAGCCCTCCAAACACAGATAGTGAAGGATGACGAGATGACCCAAAAACG

CAAAATGAGACAAGTCTTGAATCTTGGGCAAGCTACCATCAATACGAATGGAGATAATCAAGAGGAGC

TTAACCCTAATTTTCTTGATCATGACAATTACAGTAATGACGAGCACCTGAATAAGAGTGGTGCAAGAG

ATGATCATAGTTCAAACATGGCTAATAAGGAGGCCATGTTATTGGCAGCAAAAAAATCAATATCGAGCA

ATAATACTAACATGGCAAGAGTTGGCATGGGAGCTTCAATGCCAAAGGCAGAGCAGAGCCACGAAGTA

ACGATGAGACAAGCTCGTGTGTCAGTCCGAGCACGATCTGAAGCATCCATGATAAGCGATGGATGCCA

ATGGAGAAAGTATGGGCAAAAAATGGCCAAAGGAAATCCATTCCCTCGAGCTTACTATCGTTGCACCAT

CGGCACTTCTTGTCCCGTCCGCAAACAGGTTCAAAGATGTGCGGAAGATAGAAGTATTCTCATAACAA

CTTACGAAGGGCAACACAACCATCCATTGCCAACAGCGGCTATAACCATGGCCGCCACCACATCAGCT

GCTGCCTCAATGCTCGTGACGGGCTCAATGTCCAACCAGGTGGATGGGTCAATGAACCCGCCCGTCTT

CTCCCAGACCGCAACCCTCCCGGGCCTACCAAGCCTCGCCACGATCTCAGCCTCAGCCCCATTCCCAA

CTATCACATTGGACCTCACCAGGCCACAGCCGCAGGGCAACCCTAACAATAATGGGCTGGGCCTGGTA

AGCCCGCTACAGCAGATCTTCAGTGGGCCCAACAAAGAGAATGGTAATAGCAATCTGCCTTATTTGGGG

GAGCCGGTGAGTGTTGCTAGAGCAGCCCTGGCTGCTGACCCCACCCT

TGCTGCAGCCCTAGCAACTGCTATTTCTTCCATGATTAGTAATAATAAGGGGAACAATGTCAATGTAGAA

TCCCATAGTAACAATGGTGGTGAAAGGGTTGTGAGTGGAAAAAATGCCAAGGATAATCTCAACTTGTC

AATTCTGTAG

>*HuWRKY43*

ATGAAGAAGAAGGGGCAAAAGAGAGTAAGGCAACCGCGATTTGCATTCATGACAAAAAGTGAAGTTG

ATCATCTTGAAGACGGATATAGATGGCGAAAATATGGTCAGAAAGCTGTTAAAAACAGCCCATTTCCCA

GGAGCTATTATAGATGCACAAACAGCAAGTGCACAGTGAAGAAAAGGGTGGAACGGTCCTCAGAAGA

TCCAACGGTTGTGATCACCACCTATGAGGGTCAGCACTGTCATCACACCGTTGGATTCCAAAGAAGCA

CTGGATTCATTGGTCATGAAGGCCCTTTCCTAGGGCAGTTTCCTCCTCAGTTAGCCCCCCAGTACTTCTA

CCCGCCACCACCACCTGGTATGCAGTTTTCTCCCCGCGATGATTCAACTAGTACTATTAATGTACAAGCT

TCTGCTACTTCTTCTTTCCAATCTTATCAAGAATCCCCCCATGAAGTGGAAGAATCGCCACCAATGATTC

CTCAGCAATCAAGCCAACAACCGCCACCACCACCACCTCCTACCGATGAAGGATTGCTTGGGGATATC

GTGCCTCCAGGGATGCGAAACCTATGA

>*HmoWRKY43*

ATGAAGAAGAAGGGGCAAAAGAGAGTAAGGCAACCGCGATTTGCATTCATGACAAAAAGTGAAGTTG

ATCATCTTGAAGACGGATATAGATGGCGAAAATATGGTCAGAAAGCTGTTAAAAACAGCCCATTTCCCA

GGAGCTATTATAGATGCACAAACAGCAAGTGCACAGTGAAGAAAAGGGTGGAACGGTCCTCAGAAGA

TCCAACGGTTGTGATCACCACCTATGAGGGTCAGCACTGTCATCACACCGTTGGATTCCAAAGAAGCA

CTGGATTCATTGGTCATGAAGGCCCTTTCCTAGGGCAGTTTCCTCCTCAGTTAGCCCCCCAGTACTTCTA

CCCGCCACCACCACCTGGTATGCAGTTTTCTCCCCGCGATGATTCAACTAGTACTATTAATGTACAAGCT

TCTGCTACTTCTTCTTTCCAATCTTATCAAGAATCCCCCCATGAAGTGGAAGAATCGCCACCAATGATTC

CTCAGCGATCAAGCCAACAACCGCCACCACCACCACCTCCTACCGATGAAGGATTGCTTGGGGATATC

GTGCCTCCAGGGATGCGAAACCTATGA

>*HuWRKY44*

ATGTTGTATTTGTTCAGGATGGAGTTGAAAGACATAGATAGAGTAATCATAGCAAAGCCAGTAGCTTCA

AGGCCTATTTGCACTGGGTTTAGATCCTTCTCTGAGCTCCTTGCTGGTGCCATCGATGCCTCACCATCAT

CAGCTAATCTGTGCTCTGAAATGGCAGTTGCTGCGATTAGGCCTAAGACAATAAGGGTTAAGCCTGCAG

TGAATCACAGCATAGTTTCTCAGGATGAGGCTTCGAGGGCAGCTTCCGTGTCTGACAAGTTTGCAAGA

TCAGAAAATCATTCCACTATGGTATTCAAGCCGTTGGCAAAAGTGGTCTCCCGGGCTACTGTTTCTCTG

TTGGCAAGTATGGGGGATCTCAACATCACTCATCCACAGGCAGCAACACAGGTTGACGTGGGTGATCA

GCATCTGACTCAACACAAGCTTTTTCAGGTCTCGAATCCATGTCCAAGTAATCCACCCCAGGCTGAACC

ATCCCAAGTAGTTGATCCTCCTAAGATGGCACAGCAGATGCTTGATGAAGAACCTAAGATGATAGCGCC

TTCTGCAGCTAATGTTGATCGGCCCTCTTATGATGGATATAATTGGAGAAAATATGGGCAAAAACAAGTC

AAGGGAAGTGAGTATCCAAGAAGTTATTATAAGTGCACAAATCCAACTTGCCCTGTAAAGAAAAAAGT

CGAGAGATCCTTTGATGGGCAGATAGCAGAAATTGTATATAAGGGGGAGCATAACCATCCAAAGCCACA

CCCTCCCAAGCGCAACTTATCTGATGGGATTGGACAAGGTCTTCATAGCACGGAACCCAAGTTGCACG

AAAGTAGTACTTGTGCAAGAAATGAAGGGTTCGAAGGACGGATAGAGAGTCAAGTTGGAGTGGGATT

TTCTAACCCATCAACGGTTCCTGAGAAGGCTTCGGTTTTATGTCATCATCGGACTAAAATACGAAAGCAT

AATGATGGTTGTGCAACTCCTGAGGACTCATTGAAGGGTGATGATAATGTACATAAGAGCAAGAGAAG

GAAAATTGATGAACACAATGAAGCAGGAACATCAAAGGAAGGGATACAAGAACCACGTATTTGGGTG

CAAAACAACACAGAGCCTGAAATTTCAGGGGATGGCTTTCGCTGGAGGAAATATGGACAGAAGGTTG
TTAAGGGAAACCCATACCCCAGAAGCTATTACCGGTGCACTGGCCTGAAATGCAATGTTCGCAAGCATG
TAGAAAGAACATCAGATGATCCAGCTGCTTTCATCACTACATACGAAGGAAGGCACAACCATGAAATTC
CAATGAAGAACACAAAGAATACAACCTCCCCTGAGCCAGAGTCTCAAGCACTTACTTCCAAAGAGAA
ATAG

>*HmoWRKY44*

ATGTTGTATTTGTTCAGGATGGAGTTGAAAGACATAGATAGAGTAATCATAGCAAAGCCAGTAGCTTCA
AGGCCTATTTGCACTGGGTTTAGATCCTTCTCTGAGCTCCTTGCTGGTGCCATCGATGCCTCACCATCAT
CAGCTAATCTGTGCTCTGAAATGGCAGTTGCTGCGATTAGGCCTAAGACAATAAGGGTTAAGCCTGCAG
TGAATCACAGCATAGTTTCTCAGGATGAGGCTTCGAGGGCAGCTTCCGTGTCTGACAAGTTTGCAAGA
TCAGAAAATCATTCCACTATGGTATTCAAGCCGTTGGCAAAAGTGGTCTCCCGGGCTACTGTTTCTCTG
TTGGCAAGTATGGGGGATCTCAACATCACTCATCCACAGGCAGCAACACAGGTTGACGTGGGTGATCA
GCATCTGACTCAACACAAGCTTTTTCAGGTCTCGAATCCATGTCCAAGTAATCCACCCCAGGCTGAACC
ATCCCAAGTAGTTGATCCTCCTAAGATGGCACAGCAGATGCTTGATGAAGAACCTAAGATGATAGCGCC
TTCTGCAGCTAATGTTGATCGGCCCTCTTATGATGGATATAATTGGAGAAAATATGGGCAAAAACAAGTC
AAGGGAAGTGAGTATCCAAGAAGTTATTATAAGTGCACAAATCCAACTTGCCCTGTAAAGAAAAAAGT
CGAGAGATCCTTTGATGGGCAGATAGCAGAAATTGTATATAAGGGGGAGCATAACCATCCAAAGCCACA
CCCTCCCAAGCGCAACTTATCTGATGGGATTGGACAAGGTCTTCATAGCACGGAACCCAAGTTGCACG
AAAGTAGTACTTGTGCAAGAAATGAAGGGTTCGAAGGACGGATAGAGAGTCAAGTTGGAGTGGGATT
TTCTAACCCATCAACGGTTCCTGAGAAGGCTTCGGTTTTATGTCATCATCGGACTAAAATACGAAAGCAT
AATGATGGTTGTGCAACTCCTGAGGACTCATTGAAGGGTGATGATAATGTACATAAGAGCAAGAGAAG
GAAAATTGATGAACACAATGAAGCAGGAACATCAAAGGAAGGGATACAAGAACCACGTATTTGGGTG
CAAAACAACACAGAGCCTGAAATTTCAGGGGATGGCTTTCGCTGGAGGAAATATGGACAGAAGGTTG
TTAAGGGAAACCCATACCCCAGAAGCTATTACCGGTGCACTGGCCTGAAATGCAATGTTCGCAAGCATG
TAGAAAGAACATCAGATGATCCAGCTGCTTTCATCACTACATACGAAGGAAGGCACAACCATGAAATTC
CAATGAAGAACACAAAGAATACAACCTCCCCTGAGCCAGAGTCTCAAGCACTTACTTCCAAAGAGAA
ATAG

>*HuWRKY45*

ATGGTTGCAAAGGAGGAAGGCCAAGTGATTGTAGCTTCTAAAGATAACTCACTGCAGACGCCAAGTCC
TGATTCCATTGTGCATGTTTGCTATACTCACCAAGAAGAGAAAACACCCATTGTCCCAGAGAAACCATT

GGAAGATGGATATAATTGGAGAAAGTATGGGCAAAAAAATGTCAAAGGAAATGAATATATAAGAAGCTA

TTACAAGTGTTCATACCCAAACTGCCAGGTCAAAAAACAAGTGGAGCGCTTGCAGGATGGGCGGATTA

CAGATATTACTTACCTGGGGAAGCATGACCATCCTAAACCTCACTCTGAAAACCAGGAAGCTGTTTCCT

CTTTTTTGTCAGTTCAAGCACGGATAACAGATTGTCCTTCATCAACTGTTGAAGAAGATAAGTCATCCA

AAGTGCATGCCCCCGCAAATGATCATTTTCATCTTCAGCGAATGGATGTGTGTGAGCGTCCAATGGTTG

ACGAGCATGGTAGTACGACACCTTCTCTTGCAGAAACTGATCAAACAAGAGATGTTCATGATGAAAAT

GTGGTCCCAAAGAAACGGAAGAAAAGCATTGACAGTGCTGATATCATGTCAGAAAAACCTGCCCATGA

ATCACGTCTTGTTGTCCATACAGTTAGTGAGGTTGATATTGTCAATGATGGGTACAGGTGGCGCAAATAT

GGACAGAAAATGGTTAAAGGCAATCCTAATCCAAGGAGCTACTATAGGTGTTCTACTGCTGGATGCCCG

GTTAAGAAACATGTCGAGAGAGCCTCTTATGATCCACAAGTTGTCATTACCACATATGAGGGTCAACAT

GATCACAGTATACCTCCCATTAGGACTGTTGAACCCCAGAATGAATCACCATCTGGTGGCAGGATGGAA

TTGAATGATGTCAGTAAATCCAGGTTGCAAGAGTATGATGGTGCTCAGAATGGTTCAACAATATGTGAA

GCAGAGAAAATGGATGGCATTGGCGCCACATCCAAAAAAGAAATCAATACCAAAGATGCGGTCAAGAT

GGAGCCTGAAACTTCACCTAGTGAGCAAAAGATGCAGCCTGGAGCCAGAAGCAATCTAATTGAGCAA

AGGAAGCGTAGAGTTACTAGTAGTGATACACAAATGCAGGATGGTCTCATTTGCGCTGCTTCAAGTTCT

GATAATATTGATCATCAAAGTATCAAGCAAGAAAAAGTGGCCACGGATGAAGAAATGAAGACTGAGGT

GGTACCAACTGACAAACAGATAAAAACTGACAAGTCTCATCTTGAAACAAAATCAAACAAGGTCAAG

CAAGAAAAAGTGGCCACTGATGAAGAAATAATTGATGTTGGTAAGACTCAGGGGGTACCAACTGAGC

AACAGAGGCCTGACGCACGAGCTGACCTGTCTCATCCTGAAACAAAATCAAACGAGGTCAAGCTAGA

AAAAGGGGCCACTGATGAAGAAATGAAGACTGAGGTTGGTGAGACTCAGGTGGTACGAATTGATGTT

GGTAAGACTCGGGGTACCAACTGA

>*HmoWRKY45*

ATGGTTGCAAAGGAGGAAGGCCAAGTGATTGTAGCTTCTAAAGATAACTCACTGCAGACGCCAAGTCC

TGATTCCATTGTGCATGTTTGCTATACTCACCAAGAAGAGAAAACACCCATTGTCCCAGAGAAACCATT

GGAAGATGGATATAATTGGAGAAAGTATGGGCAAAAAAATGTCAAAGGAAATGAATATATAAGAAGCTA

TTACAAGTGTTCATACCCAAACTGCCAGGTCAAAAAACAAGTGGAGCGCTTGCAGGATGGGCGGATTA

CAGATATTACTTACCTGGGGAAGCATGACCATCCTAAACCTCACTCTGAAAACCAGGAAGCTGTTTCCT

CTTTTTTGTCAGTTCAAGCACGGATAACAGATTGTCCTTCATCAACTGTTGAAGAAGATAAGTCATCCA

AAGTGCATGCCCCCGCAAATGATCATTTTCATCTTCAGCGAATGGATGTGTGTGAGCGTCCAATGGTTG

ACGAGCATGGTAGTACGACACCTTCTCTTGCAGAAACTGATCAAACAAGAGATGTTCATGATGAAAAT

GTGGTCCCAAAGAAACGGAAGAAAAGCATTGACAGTGCTGATATCATGTCAGAAAAACCTGCCCATGA
ATCACGTCTTGTTGTCCATACAGTTAGTGAGGTTGATATTGTCAATGATGGGTACAGGTGGCGCAAATAT
GGACAGAAAATGGTTAAAGGCAATCCTAATCCAAGGAGCTACTATAGGTGTTCTACTGCTGGATGCCCG
GTTAAGAAACATGTCGAGAGAGCCTCTTATGATCCACAAGTTGTCATTACCACATATGAGGGTCAACAT
GATCACAGTATACCTCCCATTAGGACTGTTGAACCCCAGAATGAATCACCATCTGGTGGCAGGATGGAA
TTGAATGATGTCAGTAAATCCAGGTTGCAAGAGTATGATGGTGCTCAGAATGGTTCAACAATATGTGAA
GCAGAGAAAATGGATGGCATTGGCGCCACATCCAAAAAAGAAATCAATACCAAAGATGCGGTCAAGAT
GGAGCCTGAAACTTCACCTAGTGAGCAAAAGATGCAGCCTGGAGCCAGAAGCAATCTAATTGAGCAA
AGGAAGCGTAGAGTTACTAGTAGTGATACACAAATGCAGGATGGTCTCATTTGCGCTGCTTCAAGTTCT
GATAATATTGATCATCAAAGTATCAAGCAAGAAAAAGTGGCCACGGATGAAGAAATGAAGACTGAGGT
GGTACCAACTGACAAACAGATAAAAACTGACAAGTCTCATCTTGAAACAAAATCAAACAAGGTCAAG
CAAGAAAAAGTGGCCACTGATGAAGAAATAATTGATGTTGGTAAGACTCAGGGGGTACCAACTGAGC
AACAGAGGCCTGACGCACGAGCTGACCTGTCTCATCCTGAAACAAAATCAAACGAGGTCAAGCTAGA
AAAAGGGGCCACTGATGAAGAAATGAAGACTGAGGTTGGTGAGACTCAGGTGGTACGAATTGATGTT
GGTAAGACTCGGGGTACCAACTGA

>HuWRKY46

ATGGAGAAGATGAAGGGAAGTACTTCAATGGATCAAAGGGCTGTGATGGTCAAGGAGCTAGCTCAGG
GGCTCGAGCTAGTTAAGCGACTCAGAACGCGACTGTCAGTTAGCTCAACTGATGATGATCAAGCTCTTA
ACCACTCATTAGTGGATAAGATGCTCTCTGTCTTTAACAACTCAATTTCTTTGGCCAAGTTCAATTTAAC
TGAGCTTCATAAAGTTCGCACGGACTCGCCGCGTTCTCTGGCTCATAGCTGCCCCGGAAGTGAAGACT
CCTCTCATGATCTTCGACCGTCTAGAAAAAGGAAGAAGATGCCACCTAGGGTTGAATGGGTCCGGGTT
AGCCCGGGCGGCTCGGGTTCTGTTCTCGAAGGTCCTCCTGATGATGGTTATAGCTGGAGGAAGTACGG
ACAGAAACAAATCCTCAAAGCTAAGCATCCACGGGGATATTACCGATGCACACATCGTCACACCCAAG
GATGTTTTGCCAAAAAACAGGTCCAAAAATCCGACCAGGACCCATCCATCTACCAAGTCATCTACAGA
GGCGAGCACACGTGCCCTCAGCAGGCCCATTTGGTCCAGGCCAATGAAAAGCCCAATGAGGCCCAAA
TCAAAGTCCAACCACAGCCACAGCCACAGCCACACTCACAATCACAACCACTCCCAACCCCACAACA
ATGCCAACCCGTTGGGTCCAACCAACAAGAAGTCACTTTCGGCCTGGGCCCAGATGTGGGCTCAAAA
GTTGATCCTACAGGAATGCAGGATGATGCCTACATTTTCGGGTCCTTATCATTAAATTCGGCCCAAAGTG
AACCAGGAATAGTTCAAGACTGCATGCTGAGCTCCTCCCCTGGGTTTTGGCCACTGGCAGCCTCGGAA
TACAACTGTTTTCCGATCTCGCCATGCCAGACCAACGCTTTTGGCCTGGGCCTTAACGCGCAGACATCG

CAAGATCTGAATGAGTTGATATCCGGGCCGAGTTCGGTCTCAAATTCACCGATTGTTGGTGACTTTGAG

TACCCCTTCCACATGATGCCGGATATTGATGTCAACTTCTCTTTTGATTTTGATAATCTCTTACGTCATATG

TAG

>*HmoWRKY46*

ATGGAGAAGATGAAGGGAAGTACTTCAATGGATCAAAGGGCTGTGATGATCAAGGAGCTAGCTCAGGG

GCTCGAGCTAGTTAAGCGACTCAGAACGCGACTGTCAGTTAGCTCAACTGGTGATGATCAAGCTCTTA

ACCACTCATTAGTGGATAAGATGCTCTCTGTCTTTAACAACTCAATTTCTTTGGCCAAGTTCAATTTAAC

TGAGCTTCATAAAGTTCGCACGGACTCGCCGCGTTCTCTGGCTCATAGCTGCCCCGGAAGTGAAGACT

CCTCTCATGATCTTCGACCGTCTAGAAAAAGGAAGAAGATGCCACCTAGGATTGAGTGGGTCCGGGTT

AGCCCGGGCGGCTCGGGTTCCGTTCTCGAAGGTCCTCTTGATGATGGTTATAGCTGGAGGAAGTACGG

ACAGAAACAAATCCTCAAAGCTAAGCATCCACGGGGATATTACCGATGTACACATCGTCACACCCAAG

GATGTTTTGCCAAAAAACAGGTCCAAAAATCCGACCAGGACCCATCCATCTACCGAGTCATCTACAGG

GGCGAGCACACGTGCCCTCAGCAGGCCCATTTGGTCCAGGCCAATGAAAAACCCAATGAGTCCCAAAT

CAAAGTCCAACCACAGCCACAGCCACAGCCACACTCACAATCGCAACCACTCCCAAACCCACAACAA

TGCCAACCCGTTGGGTCAAACCAACAAGAAGTCACTTTGGGCCTGGCCCTAGATGTGGGCTCAAAAGT

TGATCCCACAGGAATGGAGGATGATGCCTACATTTTCGGGTCCTTATCATTAAATTCGGCCCAATGTGAA

CCAGGACTAGTTCAAGACTGCATGCTGAGCTCCTCCCCTGGGTTTTGGCCACTGGCAGCCTCGGAATA

CAACTGTTTTCCGATCTCGCCATGCCGGATCAACGCTTTTGGCCTGGGCCTGAACGCGCAGACATCGCA

ATCCGACCTGAATGAGTTGATATGCGGGCCGAGTTCGGTTTCAAATTCACCGAATGTTGGTGACTTTGA

GTACCCCTTCCACATGATGCCGGATATTGATGTCAACTTCTCTTTTGATTTTGATAATCTCTTACGTCATAT

GTAG

>*HuWRKY47*

ATGGTTAAGGAAATGGACTTCTTGTCAGGCTCTACTCGTTCATCATCAAATAATCATCATCATGCATCTG

TTGATGAAAACAACGATCTTGATGATGATGAACATCTTCAGCTACAAGTAGGGACAGGGTTAAATCTTC

AAACGGGTAGAGATTTGGTTGGTGCTGACAAGTCCATGGTTGACGATGAAGCATTACCAAGAAAATCA

TACAACAATCTAATTATCAAGGATAAGTTACTATTTCTGAGGGATGAGATTGCAAAGATGACAGTTGAA

AATCAAAGGTTGAAGACTTTATTGAATCAAGTAACCAACAGCTACCAAGCTCTTCAATTGCAAATCAAA

ACCATTACACAGCACCAACTTGATCAAAATCAACACGCAAAAGTCCTTCCAAAACAGATGTTCAATAA

CAAGGAAGCAATGGAAGAAGCTGGCAAAAGCCACCGAATGAGACAATTCTTGGGTCTTGGAGACGCT

GCTCATCAAGTGAATAGTAGTGATGAAGGAGATGGAGATGGTGAAGAGGAATCGATGCCGAATTCATG

TGATAACTATAGTAATAGTAATGAAGAGCACTCAAATAAGAGTGATGAAAGAGGAGATCATAGTGCAAA
AATGGCTAGGAAGGAAGCATTACTTGAGAGATCATGGGGATCCAAAAACACAACATCAACTAGTGGTA
ATATGCAAATGCCAAGAGTGGGTGTGCAAGCTCCAACAGGAGAAGCAGTAGAGCAAAGCCATGAAGC
AACTATGAGAAGAGCCCGTGTGTCTGTTCGAGCACGATCTGAAGCATCTATGATAAGTGATGGATGTCA
ATGGAGGAAATACGGGCAGAAAATGGCGAAAGGAAATCCATGCCCACGAGCCTACTACCGTTGTACTA
TGGGAACTGCATGTCCAGTTCGCAAACAGGTGCAAAGATGTGCAGAAGACCGAAGCATACTCATAACA
ACTTATGAGGGGCAGCACAACCACCCCTTACCAAACGCAGCAATAACCATGGCCACCACCACATCAGC
CGCGGCCTCGATGCTCGTATCCGGGTCAATGTCAAGCCCAATGGGCGGGCTGATAAACCCGCCCATATT
CTCTCAGCCCACAACCCTCCCGGGCCTTCCAAGCCTCGCCACGATCTCAGCCTCGGCCCCCTTCCCTAC
TATTACATTGGACCTCACTGGGCCCCACCAGCAGCAACAGGGCACCACCAACATGCCACTAGGGAACC
TTAGTGGGCCGGGCTTGGTAGGCCCAATGCAACAGGCTTTGGGCCAGGCCCTGTGTGGAGTTGACTCG
ACCCAAGTAGGGCCCAATAGGGATGCAATTAGTATGATAAAAGAAGCTCTTACTGAGCCCTCCATTGCT
ACAGCCCTAGCAGCTGCAATTTCATCCATCATGAAAGGCAATGTTGATGGTAGTGAAAATGCCAAGGAT
AAAAATCTTGGCTTGTCAAATTTTGTAGGTAATTAA

>HuWRKY48

ATGGAGGAGATGTTGAAGAAAGAAGATCAGATGAACATGATCAACAACAACAGTAGTAGTAGTAATCA
TTTCTCTGTTTTTCCAGATCATACTGGTAATGAGGTGATTGGTCATGGGAATATCAACTACAGTTGGGGA
TGTGAATATGAATACGATGAACATTATGATGAAATTGGTGGAGTTACGGAAAAGGGATTTGGATTTAATT
ATCTGGGCAGCTTTATGGATATGGTGGGAATCCAAGATTTTCCCCAAAATTCGTTATTCGATTTGTTGTCT
CTTCCTCCTCCTCCTCCAGTATTATTACCATCTTCAGATCCCCAGAATAAGCAGAATAATAATAAGCAAG
ATTGTAAGGAAGAAGGAGAAGATCAGGAGAGTTGTCAACACGACGCCGTCTAGCTCTACTCCGGCCG
CCGGAGACTCGTCGGAGGTCGTCAACACAGCGCCCGCGACACCCAACTCGGTTTCCAACATTTCTTCC
TCCTCATCCGATGCCGCCATTGATCACCCTGACCCAACTCAAAACTCAAGTCCGCATAAGTTCAAAAAT
CAGTTGAAGGTGGTGAAGAAGAAGAAGCAGAAAAGGGTAAAGGAGCCCAGATTTGCGTTCATGACA
AGGACCGAGATTGATCATCTTGATGATGGATATCGGTGGCGCAAGTACGGCCAAAAAGCCGTCAAGAA
CAGCCCCTTCCCCAGGAGCTATTACCGTTGCACCACGGCCGCATGTGGGGTGAAGAAGCGAGTAGAGA
GGTCCTGCGATGATCCCACCATCGTGGTCACCACCTATGAGGGCCAGCACACACACCCATGCCCAGTG
GTGACACGAGGCAGCCTCGGTCTCAGCCTCCTCCCACCACCACTCAATCAGCCCACCGCCACCACTTT
TGGTGATGGAGGTGGTGGTGCATCATCTGCTAGTACTTTCTCTCTCCTCCACTACCACCACCCCCACCA

CCAACTCATCCCAATGAGCACCTACAACGTTCCCAACAATACAATCAACACAGTTGCGCCACCACCTCA
TTCCTCCATGACCTTCACCTCTAGCTGTGGCGGTGGCAGTGGCGATGGTAGTGGCAGAGGCAGTCCATT
TTCTCTCTCCTCAGTGCTTATGGCTAATTACCAGTTAAGAGATGATGGGCTACTCCAGGACATAGTGCCA
TCCGCCCAGGCCAACAACGGCAGCACCACCAGTACCTAG

>*HmoWRKY48*

ATGGAGGAGATGTTGAAGAAAGAAGATCAGATGAACATGATCAGCAACAACAGTAGTAGTAGTAATCA
TTTCTCTGTTTTTCCAGATCATACTGGTAATGAGGTGATTGGTCATGGGAATATCAACTACAGTTGGGGA
TGTGAATATGAATACGATGAACATTATGATGAAATTGGTGGAGTTACGGAAAAGGGATTTGGATTTAATT
ATCTGGGCAGCTTTATGGATATGGTGGGAATCCAAGATTTTCCCCAAAATTCGTTATTCGATTTGTTGTCT
CTTCCTCCTCCTCCTCCAGTATTATTACCATCTTCAGATCCCCAGAATAAGCAGAATAATAATAAGCAAG
ATTGTAAGGAAGAAGGAGAAGATCAGGAGAAGGTTGTCAACACGACGCCGTCTAGCTCTACTCCGGC
CGCCGGAGACTCGTCGGAGGTCGTCAACACAGCGCCCGCGACACCCAACTCGGTTTCCAACATTTCTT
CCTCCTCATCCGATGCCGCCATTGATCACCCTGACCCAACTCAAAACTCAAGTCCGCATAAGTTCAAAA
ATCAGTTGAAGGTGGTGAAGAAGAAGAAGCAGAAAAGGGTAAAGGAGCCCAGATTTGCGTTCATGAC
AAGGACCGAGATTGATCATCTTGATGATGGATATCGGTGGCGCAAGTACGGCC
AAAAAGCCGTCAAGAACAGCCCCTTCCCCAGGAGCTATTACCGTTGCACCACGGCCGCATGTGGGGTG
AAGAAGCGAGTAGAGAGGTCCTGCGATGATCCCACCATCGTGGTCACCACCTATGAGGGCCAGCACAC
ACACCCATGCCCAGTGGTGACACGAGGCAGCCTCGGTCTCAGCCTCCTCCCACCACTACTCAATCAGC
CCACCGCCAACACTTTTGGTGATGGAGGTGGCGGTGCATCATCTGCTAGTACTTTCTCTCTCCTCCACT
ACCACCACCCCCACCACCAACTCATCCCAATGAGCACCTACAACGTTCCCAACAATACAATCAACACA
GTTGCGCCACCACCTCATTCCTCCATGACCTTCACCTCTAGCTGTGGCGGTGGCAGTGGCGATGGTAGT
GGCAGAGGCAGTCCATTTTCTCTCTCCTCAGTGCTTATGGCTAATTACCAGTTAAGAGATGATGGGCTAC
TCCAGGACATAGTGCCATCCGCCCAGGCCAACAACGGCAGCACCACCAGTACCTAG

>*HuWRKY49*

ATGAACATAGAGAACAAGAAGCTTACACAAACGTTGACACGAATGTGTCAGAATTACGATCTTTTACA
AAGTACGCTGATTGATTTGTTTAGTCAAACACCAAAAAAGGACAACGGTGATCATGAGAATCCTAAGA
AGAGGAAGGCTTGTTTTGAGGCTATGGATTGTCATCAAAGTCAGAGAAACTCAGAACACAGTTGCATC
ATCAGCTATGAAGAGTCCTTGAAAGAAGCTAGGCTGCTAAATTGCAAGCCTAAAGTATCCACGATCTTG
ATCCGAACCGAAAAGTATGATACAAAACTGGTTGTGAAGGATGGATATCAATGGAGAAAATATGGGCA
AAAGACTACAACGAATAACCCATATCCAAGAGCTTACTTCAAATGTGCCAGTGCCCCTATTTGTCTCGT

GAAAAAGAAGGTATACGTTTTATTTTCTTAG

>*HmoWRKY49*

ATGAACATAGAGAACAAGAAGCTTACACAAACGTTGACACGAATGTGTCAGAATTACGATCTTTTACA

AAGTACGCTGATTGATTTGTTTAGTCAAACACCAAAAAAGGACAACGGTGATCATGAGAATCCTAAGA

AGAGGAAGGCTTGTTTTGAGGCTATGGATTGTCATCAAAGTCAGAGAAACTCAGAACACAGTTGCATC

ATCAGCTATGAAGAGTCCTTGAAAGAAGCTAGGCTGCTAAATTGCAAGCCTAAAGTATCCACGATCTTG

ATCCGAACCGAAAAGTATGATACAAAACTGGTTGTGAAGGATGGATATCAATGGAGAAAATATGGGCA

AAAGACTACAACGAATAACCCATATCCAAGAGCTTACTTCAAATGTGCCAGTGCCCCTATTTGTCTCGT

GAAAAAGAAGGTATACGTTTTATTTTCTTAG

>*HuWRKY50*

ATGACAACAACATCGACTACTACTACCACCACCACCACCACTTTCAGCTCCTTGGAGTCATCGGGAAG

CGATCAGCATATAGATCTGGGCCTGGACTTTGAACTCTCAGATTTCTTGTCTCTTGAGAATTGGGCAATG

GTGGAGTCAGGATCTATGGGTTTTGGTCAAAATCAAAGTCTTGGGTGCCAAGGAAGTACAGTTTGTGA

GGTTGGAGGAACCAGCAATATCCAAAATGAGGGAGCGAATAGCATGGGGAGCTACATGGACCCAAAA

GGAGCTAGGGAAAGGGTCGCGTTCAGGATGAAGTCAGAGGCCGATGTAGTAGAAGACGGATATAAATG

GCGAAAATATGGGAAGAAGATGGTCAAGAATAGCCCCAACCCCAGGAATTACTACAAGTGTTCGGTAG

ACAATTGTCCTGTTAAGAAGAGAGTTGAACGTGATAGGGAAGATCCAACATATGTAATAACAACATATG

AAGGGCACCATAACCATGAAGCCCCTAAGAAGCAATGA

>*HmoWRKY50*

ATGACAACAACATCGACTACTACTACCACCACCACCACCACTTTCAGCTCCTTGGAGTCATCGGGAAG

CGATCAGCATATAGATCTGGGCCTGGACTTTGAACTCTCAGATTTCTTGTCTCTTGAGAATTGGGCAATG

GTGGAGTCAGGATCTATGGGTTTTGGTCAAAATCAAAGTCTTGGGTGCCAAGGAAGTACAGTTTGTGA

GGTTGGAGGAACCAGCAATATCCAAAATGAGGGAGCGAATAGCATGGGGAGCTACATGGACCCAAAA

GGAGCTAGGGAAAGGGTCGCGTTCAGGATGAAGTCAGAGGCCGATGTAGTAGAAGACGGATATAAATG

GCGAAAATATGGGAAGAAGATGGTCAAGAATAGCCCCAACCCCAGGAATTACTACAAGTGTTCGGTAG

ACAATTGTCCTGTTAAGAAGAGAGTTGAACGTGATAGGGAAGATCCAACATATGTAATAACAACATATG

AAGGGCACCATAACCATGAAGCCCCTAAGAAGCAATGA

>*HuWRKY51*

ATGGAGCAGTTGGTGCACCTCTTATCCCAGCAGCACCAGAATAATGACAATCATCATGATCATCATGAG

AATCATGGAAGCTCAAGTAGAGTGGGTGATGAAGCAGAGTGCAGAGAAGTGACTGAAGCCACAGTTT

CCAAGTTCAAGCGGCTAATCTCTGTCCTCAATCGTACTGGCCATGCCCGATTTCGCCGGGGGCCATCTA

ACCCTAACCCTAATCTTTCTCTTTCCTCTTCATCTTCCTCCTCTGGGTCTCCGCAGCCGAATCTGGTGCTT

GCTCCCGAGCCGCCCCAGAAGCCGGTCATGGCTGCCAGAGTGAGGCAGGAGCCTCAATCTCTCACCTT

AGATTTCACAAACCCTAATAATTTTCTGGCCAAATCACAACCACCATCACAACTACAACCGCAGTCACA

ATCACAATCGCAGCAGATGCCGTTTCTTTCCCTAGGCGTTTCGAAGAACAAGAACAATAATGGTGGTAG

CAACAACAACAATTGCAGCAGCAGTAGCAAATCGGGGGGTGTGGATTTGTCGTCCTTCAGCATATCTTC

CTCATCGTCTTTCATGTCGTCGATCACCGGAGAAGGAAGCGTCTCCGGCGGAAAAGGGCAGGGGATAT

TCATCCCCCCGGCGAGTTCCTCCGGCTCCGGGAAGCCTCCCCTATCTTCTTCGTCGCACAAGAAGAGAT

GCCACGAACACGACCATGACCAGGACGACTCCGACGATCTCTCCGGCAAGCTCTCAGGCTCCGGCGG

CCGCTGCCACTGCTCCAAACGAAGGAAACTGAGGGTGAAGAGAACAATAAGAGTGCCGGCGACAAG

CTCAAAGACGGCGGATATTCCGGCGGATGAGTACTCATGGAGGAAGTACGGCCAGAAGCCCATCAAGG

GCTCTCCCTTCCCGCGAGGATATTACAAGTGCAGCACGGTTCGAGGATGCCCAGCAAGGAAGCACGTA

GAGAAGGCATCGGATGATCCGAGTATGCTTATCGTCACCTACGAAGGAGATCACCGCCATGACATCCAC

CTCCCCGTCCCTCCTCCCCCGTCGCTGCCGCTCTCTCTTCCGATGGGAATGGGGTTTACCTCCTCCGCC

GCTCCGGCTTGGTTGAGTCAGCCTCAGCCTCAGCCTCAGTCAACCGCATAG

>*HmoWRKY51*

ATGGAGCAGTTGGTGCACCTCTTATCCCAGCAGCACCAGAATAATGACAATCATCATGATCATCATGAG

AATCATGGAAGCTCAAGTACAGTGGGTGATGAAGCAGAGTGCAGAGAAGTGACTGAAGCCACAGTTT

CCAAGTTCAAGCGGCTAATCTCTGTCCTCAATCGTACTGGCCATGCCCGATTTCGCCGGGGGCCATCTA

ACCCTAACCCTAATCTTTCTCTTTCCTCTTCATCTTCCTCCTCTGGGTCTCCGCAGCCGAATCTGGTGCTT

GCTCCCGAGCCGCCCCAGAAGCCTGCCATGGCTGCCAGAGTGAGGCCGGAGCCTCAATCTCTCACCTT

AGATTTCACAAACCCTAATAATTTTCTGGCCAAATCACAACCACCATCACAATTGCAACCGCAATCACA

ATCACAATCGCAGCAGATGCCCTTTCTTTCCCTAGGCGTTTCGAAGAACAAGAACAATAATGGTGGTAG

CAACAACAACAATTGCAGCAGCAGTAGCAAATCGGGGGGTGTGGATTTGTCGTCCTTCAGCATATCTTC

CTCATCGTCTTTCATGTCGTCGATCACCGGAGAAGGAAGCGTCTCCGGCGGAAAAGGGCAGGGGATAT

TCATCCCTCCGGCGAGTTCCTCCGGCTCCGGGAAGCCTCCCCTATCTTCTTCGTCGCACAAGAAGAGAT

GCCACGAACACGACCATGACCAGGACGACTCCGACGATCTCTCCGGCAAGCTCTCCGGCTCCGGCGG

CCGCTGCCACTGCTCCAAACGAAGGAAACTGAGGGTGAAGAGAACAATAAGAGTGCCGGCGACAAG

CTCAAAGACGGCGGATATTCCGGCGGATGAGTACTCATGGAGGAAGTACGGCCAGAAGCCCATCAAGG

GCTCTCCCTTCCCGCGAGGATATTACAAGTGCAGCACGGTTCGAGGATGCCCAGCAAGGAAGCACGTA

GAGAAGGCATCGGATGATCCGAGTATGCTTATCGTCACCTACGAAGGAGATCACCGCCATGACATCCAC
CTCCCCGTCCCTCCTCCCCCGTCGCTGCCGCTCTCTCTTCCGATGGGAATGGGGTTTACCTCCTCCGCC
GCTCCGGCTTGGTTGAGTCAGCCTCAGCCTCAGCCTCAGTCAACCGCATAG

>*HuWRKY53*

ATGTATCAGATGGAGAGTAATAGCATGAGTGATTCACTTGAAAAGATGCTAATGGATGAGCTAATTCAA
GGGAGAGATGTTGCTAAGCAGCTTCAATTGATTTTCTTCAAGGATCAACAGCTATCTCCTACTACCTTTT
CAAATGAGGCACATGCACAAGACTTGATGATCCAAAAGATCTTGGCTTGCTTCGACAAATCAATTACAA
TTTTGCAGTCCGATTCTGTTAATCAACTATCCCAGCAGATAGCCGTTGGAATTGCCGATTCTCCCACTTC
ATTATGTGGAAGCCCTAAAAGTCAAGATTCTGACGGAGATTTCAAAGATCCAGACCAGAAAGATGAAC
AAAAGAAAAGGCAACTTTTCCCTTTGGAGAGGAGAAAGTTGCCAGGTTGTTCAAGCAGTGGACTTGA
ACAGCCAATTGAAGATGGCTACAATTGGAGGAAATATGGGCAAAAGGACATCCTTGGAGCCAAATATC
CAAGGGCATATTACAGGTGCACTCTAAGAAAAATGCAAGAATGTTTAGCCACAAAACAAGTGCAAAGA
TGTGATAAAGACCCTACCATGTTTGAAGTCACATACCGCGGAAAACACACCTGTGCTCAAGCTGCTGC
TTCCTCCTCCAACCAGCACCCGCACCCTCATCCTAACCCTAACCCTAACCCTAACCCTAACCCGAGCCT
TGACATTAAACTCGAACCCATGGAAACCCTATCTTATCCTCACCACCAACAACAACCATCACATGAGAT
CATTTTGAGCTTCCAAATAGACCAACAAAAAGACATCAAACCCTTTAACCCTAACAATAATTATCCTAAT
AACTCGCCTTCCTTTTCCTTCCCCTCCTCTTCTAATCCTAGGGTTACCACTCACCCAGCTCCGACAACTG
TTCTTCATAATAATAATAACAACTTCACTAATCACACTAACTTTTCTCCTAATACATATGCGTGTTCTTCG
AGTGCCATGATGACCACTTTCGATCCACAAGGTCATGCTTCTTCCTCCGCGGCGAACATCAACTCTCCT
ATGGCAACAACTCCTGAGTTTCCATTTGATCAAGGCCCAATCCTCTACTCCCGCTCCTGCGCCCACAAG
CGATGGGACAAGTATCGATCAAGGGATTGCATATGTGTTAATGCTGGTGGCATTGGCATTGACGTACCTT
ATCCACTGAAGGTGCAACCGTGGAAGAGGCTTCACCTTGAGCTTTATCACCATTCTAACAAAAGTGCTT
GTGATTTTTGCATCGTTTTGTTGTTGCTCTTTTCAATTAATGGGTACTCATTCGAGTGGGGTTACATAACC
ATTGCCAATTTCCTCAATTGA

>*HuWRKY54*

ATGGATGAGAAGAAAATGATAAGAGAGGAACTTGTGCAAGTGCTGGAGAGAGGGAAAGAGTCAGCAA
TCCAGATAAGGAGACTCATTGATCATCATCATCCTCATCATCAAGATGATCATGATGTTGATGATGGGTG
TTTGAAGTTGAAGTTGAAAGGGCAGGTTGATGCTCATTCTCTGCAGGTCTTGACCTCATTTGCCACTGC
CCTTTCTATGATTCACTACAACTTTCCCTCTTTTGATGATCATCATGATCATGATCATCAAGGTCCGATCA

AAAGACCTAATCCAACTCTCAAGTCTCGTGTGTTTGGGGATTGGGATGCGCCCAACACTCCTAAGGATG
CAAGAAGAAGATCCTGCAAGAGGAGGAAGGCTGAACAATCAATGGAAATAGAAACCTCAAATGTGGC
CGATGGCTATGCTTGGAGAAAATATGGCCAAAAAGTTATCCAAAACACAAAACATCCAAGGAATTACTA
CAGATGCACACACAAAACTGAGCAAGGATGTCAAGCAACCAAACAAGTCCAACAAATCTCTGACAAT
CCTCTCAAGTACAAGATCATTTACCATGGCCAGCACAGATGCAAAACCCCCCTTAAAACCCCTCCTATG
TATAATCTTGATCTCATTGATGGTCAAGATGACTCCTCAAAGTTGCTCAGCTTTGAGAAATTACCAAACA
ACCCCAAACATGGACTTAATCCCATCCTTTTCCCATCATCATCATCATCCTCAAAAGCAACACCATCATC
ATCAAGTTCTCCTATGTGTAATGCTATGGTGGAAAAGGAGTACAAGCCCCGCATGCATGAGATCCCATC
AGAACCGTCAGATCATCATCATGAGGCCTACATCCAGTGCCCATCATCATCATCATCCGATCTCACAACT
ACTACCTTTGCTTCTGATGATGTGATCTCGTTGGCTGAGGTTTATTCCTCTGCGCTTAGTCCCTACAATGT
GGAGGTAGACTATAGCGACATGATGGCTGCTGAGTACATTGATTTCAGTCAAGTGTTTCAAGACTATGA
TCGTCTATGTTCTTAG

>*HmoWRKY54*

ATGGATGAGAAGAAAATGATAAGAGAGGAACTTGTGCAAGTGCTGGAGAGAGGGAAAGAGTCAGCAA
TCCAGATAAGGAGACTCATTGATCATCATCATCCTCATCATCAAGATGATCATGATGTTGATGATGGGTG
TTTGAAGTTGAAGTTGAAAGGGCAGGTTGATGCTCATTCTCTGCAGGTCTTGACCTCATTTGCCACTGC
CCTTTCTATGATTCACTACAACTTTCCCTCTTTTGATGATCATCATGATCATGATCATCAAGGTCCGATCA
AAAGACCTAATCCAACTCTCAAGTCTCGTGTGTTTGGGGATTGGGATGCGCCCAACACTCCTAAGGATG
CAAGAAGAAGATCCTGCAAGAGGAGGAAGGCTGAACAATCAATGGAAATAGAAACCTCAAATGTGGC
CGATGGCTATGCTTGGAGAAAATATGGCCAAAAAGTTATCCAAAACACAAAACATCCAAGGAATTACTA
CAGATGCACACACAAAACTGAGCAAGGATGTCAAGCAACCAAACAAGTCCAACAAATCTCTGACAAT
CCTCTCAAGTACAAGATCATTTACCATGGCCAGCACAGATGCAAAACCCCCCTTAAAACCCCTCCTATG
TATAATCTTGATCTCATTGATGGTCAAGATGACTCCTCAAAGTTGCTCAGCTTTGAGAAATTACCAAACA
ACCCCAAACATGGACTTAATCCCATCCTTTTCCCATCATCATCATCATCCTCAAAAGCAACACCATCATC
ATCAAGTTCTCCTATGTGTAATGCTATGGTGGAAAAGGAGTACAAGCCCCGCATGCATGAGATCCCATC
AGAACCGTCAGATCATCATCATGAGGCCTACATCCAGTGCCCATCATCATCATCATCCGATCTCACAACT
ACTACCTTTGCTTCTGATGATGTGATCTCGTTGGCTGAGGTTTATTCCTCTGCGCTTAGTCCCTACAATGT
GGAGGTAGACTATAGCGACATGATGGCTGCTGAGTACATTGATTTCAGTCAAGTGTTTCAAGACTATGA
TCGTCTATGTTCTTAG

>*HuWRKY55*

ATGGAGGAGGAAAGAAATGAGATAGTGAATCTACTCCTCCATGGATGTAGCCTGGCAAGAGATCTCGA
AGCAAATCTTCAAAACTTACCTGCTGGTAACCACGCTATGATGATCTCCCATCGATGTGATGAGATAATA
GGAGCGTTTGTTCAGGCGAGGAACCGGCTTAGCTCCCTGCAGCCTACACCACCAGCTCATCACCAGCA
AGGAAAAGGAAAGGAAGTTGCCGAAAGCTCTGTGAGGGCTGTAGATGGTGGAGGCCAAGAATTGTCG
CGTAGTGAGGCTGGTGGGAGCTTGACCTCCCCCCGACAGAGAAACCCTAGGAGATCAACCAGGAATG
ATGATCAAGGTCAGGCAGAGAGGCGAACAATAAGGAGGGTGTCGGCGCCACTGATAGGGAACACGGA
GGTGCCACCGGAGGATGGATTTACATGGAGAAAATACGGTCAGAAAGAGATCCTTAATGCCAATTACCC
AAGGAGCTATTATCGATGTACTCATCAGAAATTATATGGGTGTCCAGCCAAGAAACAAGTCCAACGTCT
CAACAGTGATCCCTTCATATTTGAGATCGTGTACCGCGGGGAGCACACTTGCCACATGAGCGCCACTGC
CCCATCAATCCCACCACCCACATCAGATCCAGGCAAGATGATCCTGGCCCATCAGGATCAAATCTCAGC
CGTCCACCATCCTCAACTCCCTTCTTGGATCCCACTGGACATCTCTCGACAATCAGGTGAGGGGGGAA
GCAGCATATTCACCCACCAACAGGAGCTCAGTAGCATCCTAGGGAGTACCAATGCACCCGCGGGCCCA
TCAGCCGTTGGATCTCATCATGATCAAAGGAGGGACGGCGTAGATTTCCTCAATCAGAATCCAGTGCTT
GATGACCACTTTGAATTGGGTGATCTGCCTGATCATATGATGATGTTTAATTCAGGGAACAACGGAAGT
AATGGCATGGAAGTTATGTTCCCTCCCTCTGATGATAAATGGGATCAGCCCGAGGATAGAAAAGATTAA

>*HuWRKY56*

ATGGATGACCACCAAGAAGCCCCACAAACTCAACCTATCCCACATCTACCATGTTCATCTCATGATCATG
ATCATGATCATCAACCATTGAACCAAACAAGCCTCAACCCTACGATTGTTCCAAATGACGACTTTGATT
GGTCCGCCCTTCTTAGCTCCCCAACTTCAGGGTTCACTTATAACCCTAATCCTATGGATAATCATGATCAT
CATCAACGACATGGCCAACAGAGTTCAATGCTTGATTATGATGGTGATAAGGGGTGTATACTAGCAGCT
GGGATGACAAACAATGGAATTACTAATTTTCATGAGGATAAGTTACGGGATAAGAGGAAGGAGATGACT
AGTTCGAGGTCAAGAAAGACTACGGTTCCTCGGTTTGCTTTCCAAACGAGAAGTTCTCAGGATATTCTT
GATGATGGGTATCGTTGGAGGAAATATGGCCAAAAATCCGTCAAAAATAGCATCTATCCTAGGAGCTAC
TATAGGTGCAGTCATCTGACATGCAGTGTAAAGAAACAGGTGCAGAGACTATCCAAAGACTCGAGCAT
GGTCGTGACAACATATGAAGGAGTCCATAATCACCCTTGTGAGAAGTTGATGGAAGCCCTTTCTCCGCT
TCTTGACCAGATGAAATTTCTTTCTAGTTTAGGCTAA

>*HmoWRKY56*

ATGGATGACCACCAAGAAGCCCCACAAACTCAACCTATCCCACATCTACCATGTTCATCTCATGATCATG
ATCATGATCATCAACCATTGAACCAAACAAGCCTCAACCCTACGATTGTTCCAAATGACGACTTTGATT

GGTCCGCCCTTCTTAGCTCCCCAACTTCAGGGTTCACTTATAACCCTAATCCTATGGATAATCATGATCAT
CATCAACGACATCGCCAACAGAGTTCAATGCTTGATTATGATGGTGATAAGGGGTGTATACTAGCAGCT
GGGATGACAAACAATGGAATTACTAATTTTCATGAGGATAAGTTACGGGATAAGAGGAAGGAGATGACT
AGTTCGAGGTCAAGAAAGACTACGGTTCCTCGGTTTGCTTTCCAAACGAGAAGTTCTCAGGATATTCTT
GATGATGGGTATCGTTGGAGGAAATATGGCCAAAAATCCGTCAAAAATAGCATCTATCCTAGGAGCTAC
TATAGGTGCAGTCATCTGACATGCAGTGTAAAGAAACAGGTGCAGAGACTATCCAAAGACTCGAGCAT
GGTCGTGACAACATATGAAGGAGTCCATAATCACCCTTGTGAGAAGTTGATGGAAGCCCTTTCTCCGCT
TCTTGACCAGATGAAATTTCTTTCTAGTTTAGGCTAA

>*HuWRKY57*

ATGAGCAATCCGGGTAAACCCGACCCAGACGAGTCGAGCTGGTCAGTGCCGGATTCAGATAACCAGCT
CTACTTCTTCCACCATCATCATCATCAAATGCTCGCCGAGTTAGGCTGGAATTATACTGATCATCAGCTG
GAGCAGAGGCTCCCCGAATTTCCACCATCATCTCCCCTTGCCGACGGGGATCAATACCATCCATCCGAT
GCTCCGCCACATACGGTTACCACTAGCACTTCCTCTCAGTTACCACCTGATCGGAGCTCCGACCCGGCT
CCTTCAACTTCGAATCCCTCAGTATCCTCCAGCTCGTCCGAAGATCCGGCTGAGAAATCGGGCGTCCGT
ACGGCTCCCACACCCGAGATTTCGACTAAGGAGAAGAAGAAGGTTCAGAAAAGGGTAAGGCAACCCA
GGTTTGCGTTCATGACAAAGAGTGAGGTTGATCATCTTGAAGATGGTTATAGATGGAGAAAATATGGCC
AAAAAGCTGTTAAAAACAGTCCATTTCCCAGGAGCTATTACAGATGCACAAACAGCAAATGCACAGTG
AAGAAAAGAGTGGAACGGTCCTCTGAAGATCCAACCATTGTGATAACCACATATGAAGGGCAGCACTG
TCATCATTCAGTGGGGTTGCATAGAAGTGGTGCCGGATTCCTCGGTCGCCGTGAAATCGGTGCCATTAA
TGTAGGGCAGTTTGGTGCAGCTTCTCCAACTTCAACATCCCAGTTTTTGATTTATCCAGGAATGATGCAA
TTCTCTCATTATGAGCAACATACTTCAGAAAGCATTGCACAAGGCTCAGTTGGTGCTACCACCACCACC
ACTACTACTCAGTATTCTCATCAGCAATTAGCTGCCACTGGTGATGAAGAGAAAGAAACCCTGCAATCA
AGCACTGGTGAAGGCTTGCTTGGAGATATTGTGTCTCCTGGAATACGAAATCTATGA

>*HmoWRKY57*

ATGAGCAATCCGGGTAAACCCGACCCAGACGAGTCGAGCTGGTCAGTGCCGGATTCAGATAACCAGCT
CTACTTCTTCCACCATCATCATCATCAAATGCTCGCCGAGTTAGGCTGGAATTATACTGATCATCAGCTG
GAGCAGAGGCTCCCCGAATTTCCACCATCATCTCCCCTTGCCGACGGGGATCAATACCATCCATCTGAT
GCTCCGCCACATACGGTTACCACTAGCACTTCCTCTCAGTTACCACCTGATCGGAGCTCCGACCCGGCT
CCTTCAACTTCGAATCCCTCAGTATCCTCCAGCTCATCCGAAGATCCGGCTGAGAAATCGGGCGTCCGT
ACGGCTCCCACACCCGAGATTTCGACTAAGGAGAAGAAGAAGGTTCTGAAAAGGGTAAGGCAACCCA

GGTTTGCGTTCATGACAAAGAGTGAGGTTGATCATCTTGAAGATGGTTATAGATGGAGAAAATATGGCC

AAAAAGCTGTTAAAAACAGTCCATTTCCCAGGAGCTATTACAGATGCACAAACAGCAAATGCACAGTG

AAGAAAAGAGTGGAACGGTCCTCTGAAGATCCAACCATTGTGATAACCACATATGAAGGGCAGCACTG

TCATCATTCAGTGGGGTTGCATAGAAGTGGTGCCGGATTCCTCGGTCGCCGTGAAATTGGTGCCATTAA

TGTAGGGCAGTTTGGTGCAGCTTCTCCAACTTCAACATCCCAGTTTTTGATTTATCCAGGAATGATGCAA

TTCTCTCATCATGAGCAACATACTTCAGAAAGCATTGCACAAGGCTCAGTTAGTGCTACTACTACTCAGT

ATTCTCATCAGCAATTAGCTGCCACCGGTGATGAAGAGAAAGAAACCCTGCAATCAAGCAGTGGTGAA

GGCTTGCTTGGAGATATTGTGTCTCCTGGAATACGAAATCTATGAAAGGGCAATTCCAGCACACTGGCG

GCCGTTACTAGTGGATCCGAGCTCGGTACCAAGCTGGCGTAATCATT

>*HuWRKY58*

ATGGTGGACAACGACGGTGCACCGTCAACAAGGCCGCAGAAGCCGACGATAACGCTGCCGCCTCGAC

CTGGGCTGGAGTCCCTCTTCATCGGGTCTGGCTTCGGGCTGAGCCCGGGCCCGCTGACTTTGGTCTCG

AGCTTCTTCTCCCCCTCCGATGCCCGCGATCCTTATTCTTCCTCCGACTGCCGCTCTTTCTCTCAGCTCC

TCGCCGGCGCTATGGCCTCGCCGGGGCCCACCGGTTCAGCGGAGCCGGACCAGGTTCGGTTCAAGCA

GAACCGGCCTGCTGGTCTGATGATTTCTCCACAACAGCAGCAAGGGGTTTTCATGATTCCCCCTGGACT

TAGTCCCACCAGCTTGCTTGATTCTCCCGGTTTATTTCCTGGCCAGGGCCCATTTGGGATGTCCCACCAG

CAGGCGCTAGCTCAGGTTACTGCTCAAGCTCAAGCTAACTTGCAAATGCAAGGTAGTTTTCCATCTCCA

TTAGGACTAGAGTTCCAAGCATCAGCTCCATTTCCTTGTTTCACTACTGATCCAATGCAACAGAATATGG

TGCCATCTGCGCCAGATTCTAAAGAACAAAAAGATCCATCCCAATTTTCTGGTTCTGATCAAATATCTCA

ACCCTCCTCGATGATTGTTGATAAGCCTGCTGATGATGGTTACAACTGGAGGAAATATGGGCAGAAACA

AGTAAAAGGAAGTGAGTTTCCACGAAGCTATTACAAATGCACGCACCCTAGTTGTCCTGTCAAGAAGA

AGGTGGAGCGCTCTCTTGATGGCCAAGTGACTGAGATAATCTACAAGGGCCAGCATAATCATCAACCA

CCTCTGAAACGTTCAAAAGATAGTGGAAATTCTAATGGCAATGTCGGAACTCAAGGAAATCCTGAGCC

TGGGTTGCAGTCTCAAGCTGGCAACATCAATGGCAGAAGAGACCAGGAAACTAGCCAAGTTACAGAA

GAGCATCTGTCCAGTGATGGGGAGGAAGTGGTTGATGATGGAGCTAGAGAATCTGAAAGAGATGAAGA

TGAACCTTATGCCAAGAGGAGGAATACGGAATTCAGGGCGTCAGAGCGAGCTTCATCACACAGAACAT

TAACAGAACCCAAGATTATTGTCCAAACAACTAGTGAAGTCGATCTTTTGGATGATGGTTACAGGTGGC

GCAAATATGGACAAAAAGTTGTCAAAGGAAATCCATATCCAAGGAGCTATTATAAATGCACTACCCCAG

GATGCAATGTCCGAAAGCATGTTGAGCGTGCGGCCTCAGACGCTAAAGCAGTCGTAACGACATATGAA

GGCAAACATAATCATGATGTTCCGGCTGCTAGAACGAGTAGCCATAGTACAGCCAACAGTGCAGCACA

ACTGAAAACTGCAAATAACAACAGCTATGCTGGGAAGACGGATTTATGAAAAACAATCAACAGCCTA

GAGCACTTTTACAATTAAAGGAAGAAAGAATAACATAG

>*HmoWRKY58*

ATGGTGGACAACGACGGTGCACCGTCAACAAGGCCGCAGAAGCCGACGATAACGCTGCCGCCTCGAC

CTGGGCTGGAGTCCCTCTTCATCGGGTCTGGCTTCGGGCTGAGCCCGGGCCCGCTGACTTTGGTCTCG

AGCTTCTTCTCCCCCTCCGATGCCCGCGATCCTTATTCTTCCTCCGACTGCCGCTCTTTCTCTCAGCTCC

TCGCCGGCGCTATGGCCTCGCCGGGGCCCACCGGTTCAGCGGAGCCGGACCAGGTTCGGTTCAAGCA

GAACCGGCCTGCTGGTCTGATGATTTCTCCACAACAGCAGCAAGGGGTTTTCATGATTCCCCCTGGACT

TAGTCCCACCAGCTTGCTTGATTCTCCCGGTTTATTTCCTGGCCAGGGCCCATTTGGGATGTCCCACCAG

CAGGCGCTAGCTCAGGTTACTGCTCAAGCTCAAGCTAACTTGCAAATGCAAGGTAGTTTTCCATCTCCA

TTAGGACTAGAGTTCCAAGCATCAGCTCCATTTCCTTGTTTCACTACTGATCCAATGCAACAGAATATGG

TGCCATCTGCGCCAGATTCTAAAGAACAAAAAGATCCATCCCAATTTTCTGGTTCTGATCAAATATCTCA

ACCCTCCTCGATGATTGTTGATAAGCCTGCTGATGATGGTTACAACTGGAGGAAATATGGGCAGAAACA

AGTAAAAGGAAGTGAGTTTCCACGAAGCTATTACAAATGCACGCACCCTAGTTGTCCTGTCAAGAAGA

AGGTGGAGCGCTCTCTTGATGGCCAAGTGACTGAGATAATCTACAAGGGCCAGCATAATCATCAACCA

CCTCTGAAACGTTCAAAAGATAGTGGAAATTCTAATGGCAATGTCGGAACTCAAGGAAATCCTGAGCC

TGGGTTGCAGTCTCAAGCTGGCAACATCAATGGCAGAAGAGACCAGGAAACTAGCCAAGTTACAGAA

GAGCATCTGTCCAGTGATGGGGAGGAAGTGGTTGATGATGGAGCTAGAGAATCTGAAAGAGATGAAGA

TGAACCTTATGCCAAGAGGAGGAATACGGAATTCAGGGCGTCAGAGCGAGCTTCATCACACAGAACAT

TAACAGAACCCAAGATTATTGTCCAAACAACTAGTGAAGTCGATCTTTTGGATGATGGTTACAGGTGGC

GCAAATATGGACAAAAAGTTGTCAAAGGAAATCCATATCCAAGGAGCTATTATAAATGCACTACCCCAG

GATGCAATGTCCGAAAGCATGTTGAGCGTGCGGCCTCAGACGCTAAAGCAGTCGTAACGACATATGAA

GGCAAACATAATCATGATGTTCCGGCTGCTAGAACGAGTAGCCATAGTACAGCCAACAGTGCAGCACA

ACTGAAAACTGCAAATAACAACAGCTATGCTGGGAAGACGGATTTATGAAAAACAATCAACAGCCTA

GAGCACTTTTACAATTAAAGGAAGAAAGAATAACATAG

>*HuWRKY59*

ATGGATAGAAACTACCAAACCACCACTCCAATGACTACTACCACTTTTAGCTCCCTAGAATCCTCAGGG

AGTGATCAGTATATCGAGGGCACAGACTTCGAGGTCTCAGAGTTCTTCTCGAATTTCGAGTGGGGGAC

GGCAGAGACAGAATCCATGGATTTTATTGGGCAGAGCCAGGATTTTGGATACCAAGGAAATGAGTTCTA

TGACCAATTTGGAGGAAGTAGCAACCAGCAACAAAATGTGGAAGACAGTAGCAGAGGCAGCAGAAAT

AGTAAGGAGAAGAAGGATGATAGGGGAAGGATTGCATTCAGAATGAGATCGGAGATTGATGTTGTAGA
AGATGGTTACAAATGGAGAAAATACGGGAAAAAGATGGTCAAGAATAGTCCAAACCCAAGGAACTACT
ACAGGTGTTCAGTAGAAGGCTGTCCAGTGAAGAAAAGGGTCGAAAGAGACAAGGAGGATCCACACTA
CGTAATAACAACGTATGAAGGCATCCATAGCCATGACGCTCCAAGGCAATGA

>*HmoWRKY59*

ATGGATAGAAACTACCAAACCACCACTCCAATGACTACTACCACTTTTAGCTCCCTAGAATCCTCAGGG
AGTGATCAGTATATCGAGGGCACAGACTTCGAGGTCTCAGAGTTCTTCTCGAATTTCGAGTGGGGGAC
GGCAGAGACAGAATCCATGGGTTTTATTGGGCAGAGCCAGGATTTTGGATACCAAGGAAATGAGTTCTA
TGACCAATTTGGAGGAAGTAGCAACCAGCAACAAAATGTGGAAGACAGTAGCAGAGGCAGCAGAAAT
AGTAAGGAGAAGAAGGATGATAGGGGAAGGATTGCATTCAGAATGAGATCGGAGATTGATGTTGTAGA
AGATGGTTACAAATGGAGAAAATACGGGAAAAAGATGGTCAAGAATAGTCCAAACCCAAGGAACTACT
ACAGGTGTTCAGTAGAAGGCTGTCCAGTGAAGAAAAGGGTCGAAAGAGACAAGGAGGATCCACACTA
CGTAATAACAACGTATGAAGGCATCCATAGCCATGACGCTCCAAGGCAATGA

>*HuWRKY60*

ATGGATTACTCAAATTCAACAACGAATTGGTTGATGAGTTCAGCCTCCTTGGAATTGGAGCTCAGCAGC
AGCAATGGCGGAAGCACTCTCCCACTCTTCGACGAGTCTCCTGTTCGCGATAACTCCTCCAGGAAGCT
CGGCGTTCAGCACAGCGTCTCCCCCTCGATTGAAGTCGCCAGCCAACTCTGGCTCAACAAGGATGAGG
CGGGAGCTTTGGTGGAAGAGTTGAACAGGGTGAACGCGGAAAATAAGAGGCTAACGGAAATGCTAAC
GGTCGTGTGTGAGAATTACAACGAATTAAAGAGGAAGTTGACGGATTACATGAGCAACGGTGCTACTA
GCATCAATAACGAGACTGAGAGTAGCAACCCGGTCAAGAAAAGGAGGACAGAGAATCTCTCTAATGG
CAACAATAAAAATAGCAATGTTGAGAGTATCTCGAGCGATGAAGAAGATTCGTATGGAAAACCAAAGG
AGCTAGAGACCGTGAAGGCGAAGATCACTAGGGTTGCTGTTCGAACGGAAGCTTCTGACTCAAGCCTT
ATACTTAGAGATGGATATCAATGGAGGAAATACGGGCAAAAGGTGACTAGGGACAATCCTTGCCCTAGG
GCTTACTTCAAGTGCTCATTTGCTCCTACTTGCCCTGTAAAGAAGAAGGTTCAAAGAAGTCTAGAAGA
CCAATCCATGCTGGTAGCGACTTACGAAGGAGAGCACAACCATCCACCCCCAGTTCAACCAGAGGCAA
CTCCAGGTTCAGGCGTAAGCCGAAGCGTCGCCCCAACCCTAGGCTCGGTCCCCACCTTAGGCTCGGCC
GTGCCCACGGTCACCCTTGACCGAGCAAAGCCCAAGCCCAACAACATTACAAATGACACACCCAACC
CCACCAAAATGCTGACCAACACAAGTACTGGTAGCGAGGCCCAAGCCCAGCCAATGAAATTCAACTCC
TCAGAATTCCAGAAGTTCTTGGCAGAGCAAATGGCCTCCTCCTTGACAAAAGATCCCAACTTCACCGC
CGCCCTTGCCGCTGCCATTTCCGGGCGGTTTGCCCAGCGGAGTTCAAGTGATTAG

>HmoWRKY60

ATGGATTACTCAAATTCAACAACGAATTGGTTGATGAGTTCAGCCTCCTTGGAATTGGAGCTCAGCAGC

AGCAATGGCGGAAGCACTCTCCCACTCTTCGACGAGTCTCCTGTTCGCGATAACTCCTCCAGGAAGCT

CGGCGTTCAGCACCGGGTCTCCCCCTCGATTGAAGTCGCCAGCCAACTCTGGCTCAACAAGGATGAGG

CGGGAGCTTTGGTGGAAGAGTTGAACAGGGTGAACGCGGAAAATAAGAGGCTAACGGAAATGCTAAC

GGTCGTGTGTGAGAATTACAACGAATTAAAGAGGAAGTTGACGGATTACATGAGCAACGGTGCTACTA

GCATCAATAACGAGACTGAGAGTAGCAACCCGGTCAAGAAAAGGAGGACAGAGAATCTCTCTAATGG

CAACAATAAAAATAGCAATGTTGAGAGTATCTCGAGCGATGAAGAAGATTCGTATGGAAAACCAAAGG

AGCTGGAGACCGTGAAGGCAAAGATCACTAGGGTTGCTGTTCGAACGGAAGCTTCTGACTCAAGCCTT

ATACTTAGAGATGGATATCAATGGAGGAAATACGGGCAAAAGGTGACTAGGGACAATCCTTGCCCTAGG

GCTTACTTCAAGTGCTCATTTGCTCCTACTTGCCCTGTAAAGAAGAAGGTTCAAAGAAGTCTAGAAGA

CCAATCCATGCTGGTAGCGACTTACGAAGGAGAGCACAACCATCCACCCCCAGTTCAACCAGAGCCAA

CTCCAGGTTCAGGCGTAAGCCGAAGCGTCGCCCCAACCCTAGGCTCGGTCCCCACCTTAGGCTCGGCC

GTGCCCACGGTCACACTTGACCGAGCAAAGCCCAAGCCCAACAACATTACAAATGACGCAGCCAACC

CCGCCAAAATGCTGACCAACACAAGTACTTGTAGCGAGGCCCAAGCCCAGCCAATGAAATTCAACTCC

TCAGAATTCCAGAAGTTCTTGGCAGAGCAAATGGCCTCCTCCTTGACAAAAGATCCCAACTTCACCGC

CGCCCTTGCCGCCGCCATTTCCGGGCGGTTTGCCCAGCGGAGTTCAAGTGATTAG

>*HuWRKY61*

ATGCCTGATCCAACGTCGAAATCTTCTGATCATGCAGGAGAGAACAGTGATGAAACTCACCGAAAAAG

GGCTGAGGCTGACCCTAATTCTCCAAACAATACCACCAACGATGATGAAATGCAGAAGGTGCAGGCTG

GAGAACTTGAATCAGCAAAAGCTGAAATGGGTGAAGTGATGGAGGAAAACCAAAGGCTTAAGCAATA

TCTAGATCAAATCATGAAGGATTATCAAGCACTACAAATGAAATTTTATGAAATCACCCAACAAGCAGC

AGACTCCAAGAACCCTAACAAAACCAACTTGATCATCGCAAATTCTTCCCCTCTTCCTTCTCGAGCAAT

AATGGAAGATGATCAAGACGAAGCAGAACTTGTTTCCCTTAGCCTAGGGCGAACTTCGAGTCTCGATT

CAAAGAAAAGCATTAGCAACAAGTCAAACATCCAAGCTGATAAGCTGGTAAACAAAGAAAAAGATGT

AGCTAATGGGAGTAAGAGTTATAATGTAGAGGGTCCTATGGAGCGAAGCCTGGATCTTGGATTAGACTG

TAAGTTCGAAACATCAAGAACAACATCGCTTGACCATCGGCAAGCGCTAATGCTGAGTTCAAGCCCAG

ATAATAGTTTAGATGACATGAAGGATGGGGAAGCTGGTGAAAAACAGACAAGTACAGCACTTAAGAGA

AGTGGCAGTGGTGATGATGAAGTGGTGCAACAACCCCCTGTGAAGAAGGCTAGGGTTTCTGTCAGAG

TTAGATGTGATACCCCTACCATGAATGATGGATGTCAATGGAGAAAATATGGGCAGAAAATTGCAAAGG

GAAATCCATGCCCGCGAGCATACTATCGTTGCACTGTTGCTCCATCCTGTCCTGTGAGGAAACAGGTAC

AAAGATGCGCAGAAGACATGTCAATCTTGATAACAACGTATGAAGGAACTCACAATCATCCACTTCCA

GTCTCAGCAACTGCTATGGCTTCCACAACCTCGGCTGCTGCTTACATGTTGATGTCTGGGTCATCAAGC

TCTGGTCATCAAGGATCCAACTCGACCACGACGACGACACTAGCTACCACAGGAGCTGAGCTCCATAG

TGGCCTTAACTTCAATATACCCGATAGCTCGAAACTAAAACCATTGTACTTACCCAACTCATCCTCTACA

TTTTTGCCTGCTCCAACCCACCCAACCATAACGCTTGACCTTACTTCCACACCCTCATCTTCTGCTACCC

AATTCAACAGGGCAACTCCTAGTTTTGCACCTAGATACCCCTCCACAAACCTCAACTTCAATTCCTCAA

ATAATCCCAACAATAACCCTTCCATCTCTTGGCCTAATATTGGCCTTCTTAACTATGCCACTCAACCACCT

AACAACAAAAGCCAATTTAGCCCACTAGACTTTGGCTCATCAAGACACCCTACTCTTCAAGATGCATCC

TCCTTCAACTTCCAAACTTTCTTGCAAAAAGGCAACCCTAATAATCCCTCTAATCCACCACCTGTACCC

GACACAATTGCCCAAGCAGCCAAAGTACTTACTTCGGATCCTAGTTTCCAAGCAGCTCTAACTGCAGCC

CTCTCATCCTTCATTAGCAGCAGCAATGGCAACGTTGGTGCTGGGATTCTAGGAGGTGACATTAACATT

AGTGATCTTGCACAGAAGATTAAGTGGGGTGGTGTTCAAACATCTTCAAATGTTCACTCATCAACATCT

CAATCCACAAACAAGGGGACAAATAATGGATGTGGATCGAGATTATTGAGCATGGCTTCTTCTGCAACA

AATAATTCTCAACCTAATGGAAGCTTGATGTTCCTCCCTCCTCCATTGCCTTTACCTGGATCTAAAAGCA

CCTCTACTTCTCCAGGTGATAGTAGAGACAATGCTAACTGA

>*HuWRKY62*

ATGGATGAGAAAGTGACGAGAGAGAAGCTTGTGCAAGTGTTGGAGAGAGGCAAAGAGTCAGCAACC

CACATCAGGAGACTAATTGATCATCATGATAGTGGGTTCTTGAAGGTCTTGAAAGGCCAGCACCTTGAT

GCTCATTGTTTGGATGTCTTAAACTCATTTGCTACTGCCCTTTCTATGATTCCCTACTACTTTCCCTCTTTT

AATGATCATCATGATCATGATCATGATCATCAAAGTCCGATCAAAAGGCCTAATCCAATCCTGAAGTCCC

AAGTGGCTGAGGAGTACTTGGAGGCTCCTAATGCCCCAAAGGATGCAAGAGGGTCCTACAAGAGGAG

GAAGACAGAACGAGCAGTGGAAATAGAAACCCAACATGTGGGCGATCGCTATGCTTGGAGAAAATATG

GGCAGAAATTTATCCAACACACAAAGCACCCAAGGAACTACTACAGATGCACACACAAAACGGAGCA

AGGGTGCCAAGCAACAAAGCAAGTCCAACAAATCTCAGACAATCCTGCAAAGTACAAGATCATCTACT

ATGGGCATCACACTTGCAAAAGCCCCCTTAAAAACCCTCCTATGTATCTTGATCTCATTGACGACCAAC

ATAAAAATTCCTCCATTTTGCTCAGCTTCGAAGCACCAAACAACCCCAAACATGGCCTTAACCCCTTCA

TGCTTTCATCATCATCGTTGTCGTCTTCATCATCAACAACAACATCAGCTGCTCCCATGAGTACAGTGGA

TCTCATTGATGAACTAGAATGTACACCCATCAAGGATATGATCCCATTGGAGCAATTGGATCAGCATCAA

GGAGCCAATATCCAGTGCCCATCATCGTCCGATTTTCTTTCTACCACTTCTGATCTCACAAGTACTAGCT
TTGTGTCTGATGATGTGATCTCATTAGCTGAGATTTATTACTCTGCGCTTAGCCCCTACGGTGTGGGGGA
AGACTATAGCTACATGAATTATGAGTCCTTTGATTTCAGCCAAGTGTTTCAAGATAGTTACCTTCTTCGT
GCATGA

>*HuWRKY63*

ATGGGGAATGTGGACGTACCACCTGAGGATGGCTATACTTGGAGAAAATATGGCCAAAAAGAGATTCTT
CATGCCAAGTATCCCAGAAGTTACTACAGATGTACACATCAAAAGTTATACAATTGCCCAGCTAAGAAG
CAAGTCCAACGACTAAACGACGACCCTTTTACGTTCGAGATCCTCTACAGAGGGGAACACATTTGCCA
CAAAGCCTCCACTGCTCCGATGGCCCCACCACAACGATCTCAAACCGCCAAAGACATCATCTCTCCTC
ACAAGCCAATCGCAACCGTTCATTCTCTGCTAAATCAGTGGTTACCAGTGGACTTTCCCACCCCGCTCA
CTGAAGGCGCGAGCACCGTAACGCAGCCCACCAAGGAGCCTTCTGCATTGGGTTTGACCAGATGCATC
AGTAACCCATGGAACTACAATGCATCCCCGGGACCCTCCACCGTCGGATCTCATCATGATGGAATGGAC
GTCGCTGATTTTCTTAATCATACGGTGGAGGACTTTGTTGCTCATGTCAATGTGATGCTCAATTCTGGCA
GTAATTGTAGCAGCAGTAGCATGGAGGTCAATTTTCCTGCCTTCGATAACAAATGA

>*HuWRKY64*

ATGATCTCCCATCGATGTGATGAGATAATAGGAGCGTTTGTTCAGGCGAGGAACCGGCTTAGCTCCCTG
CAGCCTACCCCACCACTCATCACCAGCAAGGAAAAGGAAAGGAAGTTGCCGAAGCTCTGTGAGGGCT
GTAGATGGTGGAGGCCAAGATTGTCGCGTAGTGAGGCTGGTGGGAGCTTGACCTCCCCCCGACAGAG
AACCCTAGAGATCAACACGGCAGAGAGGCGAACAATAAGGAGGGTGTCGGCGCCACTGATAGGGAAC
ACGGAGGTGCCACCGGAGGATGGATTTACATGGAGAAAATACGGTCAGAAAGAGATCCTTAATGCCAA
TTACCCAAGGAGCTATTATCGATGTACTCATCAGAAATTATATGGGTGTCCAGCCAAGAAACAAGTCCA
ACGTCTCAACAGTGATCCCTCCATATTTGAGATCGTGTACCGCGGGGAGCACACTTGCCACATGAGCGC
CACTGCCCCATCAATCCCACCACCCACATCAGATCCAGGCAAGATGATCCTGGCCCATCAGGATCAAAT
CTCAGCCGTCCACCATCCTCAACTCCCTTCTTGGATCCCACTGGACATCTCTCGACAATCAGGTGAGGG
GGGAAGCAGCATATTCACCCACCAACAGGAGCTCAGTAGCATCCTAGGGAGTACCAATGCACCCGCGG
GCCCATCAGCCGTTGGATCTCATCATGATCAAAGGAGGGACGGCGTAGATTTCCTCAATCAGAATCCAG
TGCTTGATGACCACTTTGAATTGGGTGATCTGCCTGATCATATGATGATGTTTAATTCAGGGAACAACGG
AAGTAATGGCATGGAAGTTATGTTCCCTCCCTCTGATGATAAATGGGATCAGCCCGAGGATAGAAAAGA

TTAA

>*HuWRKY65*

ATGGAGGGCATCACAAGCTGCTTCACTACTACTACTGCTACTACTGCTATTACTGATCATCATCGCTTGTA
TCAATTGGAGGAGAGAGAAAACTCGTCGGATAACAGTGGAGATAACTCTCCATCCTCCACTGCCTTCG
CATCCTCGATCCCCTCCCCTAAGAGAAGTAGGCGAGCTATACAAAAGAAGGTGGTCACGGTCCCTATCA
AGGATGTGGAGGGTTCGAGGTTTAAGGGTGATTGTACGCCACCGTCTGATTCATGGGCTTGGAGGAAG
TACGGCCAGAAACCTATCAAGGGCTCTCCTTATCCTAGGGGGTACTATAGATGTAGCAGCTCCAAGGGG
TGCCCAGCAAGGAAGCAAGTAGAGAGAAGCCGTGCAGATCCAACCATGCTTGTCATCACTTACTCTTA
CGAACACAACCACGCCTGGCCCACTTCTCGAAACCGAGACAAATCGGCTGCCACCACCACCACCAAC
GCATCCGCTACCGCCACAGCTGCCACCACGAACACCCCCAATGCCACCGCCATCGCCACCGCCGTCCC
AACCTCTCTTCCCAAAATAGAGATCAACCACAGTAACCACCCTCACCAACTCGGCGGCCACGATCAGC
CCTTGATGGTCCCCACCGACGAGCTGACCTGGCTCACCTCCGACTCCAACATGTCGTCGTTCTTCTCAA
ATTGCGCGTTTGGAGACCTTGACATGGCGAAGATATTTCCTTTGGGGGAAGAAGAAGCCCTCTTT
GCCGACCTCGGTGAGCTGCCCGAGTGCTCCGTCGTCTTCGGGCAGCCACCCACGGGCATGAGAGAGA
AAGAGGAGCAGCAGCAACAAGAGTGCGGCTTCAACTGGTGA

>*HuWRKY66*

ATGGATAGTTACTCTAGTCCTGAGAATCTGGAATCACGCAGATGCAGATCCAAGGAAGCCCAGTCAGG
GAATGAGGCCTCCAAGAAAAGGAAGCAGGAGTTGTTTGAGAATGAAAAGAGACTGGTGAGGGTTAA
GGTTGAAGCAGTAACAAATGGTGAAGGTGATGCTGATCATCACAAACAGAAACACAAGAGTGAACTA
GGGTCCCCTCCTCCACCTCCGGATTCATGGTCCTGGCGGAAATATGGTCAAAAACCCATCAAAGGATCT
CCTTATCCCAGGGGTTACTACAGGTGCAGCACAGGCAAGGGATGTTTGGCCAAGAAACAAGTAGAAG
GGTGCAAAGAAGATGCTTCCGTCTTCATCATCACTTACACCTCTCCCCACAACCACAACCGTATTGGTC
ATGCAGGGCCAATGGCCAACTCGAGCACCCAAAAGCATCGACCTCATTCTCCTGACAAACGAACCCAC
AGCAGCAATAATAAGCACACATGTGTTGAGCATCAGCACAGCAGCTCAAATGCAATGCCAATGGCAAA
ACAGGAGGCACAAGAGCCAGAGCCTTATGTGGTGAGCCTCGATGGTATTACCCCTCTGCCTCTCCCTCT
ATCTCATCATGATCAACAGCACCCCTTTGCTTCATATGATCTCAAGAGTCATCCCGCATCACAAGGAAAC
TAG

>*HmoWRKY66*

ATGGATAGTTACTCTAGTCCTGAGAATCTGGAATCACGCAGATGCAGATCCAAGGAAGCCCAGTCAGG
GAATGAGGCCTCCAAGAAAAGGAAGCAGGAGTTGTTTGAGAATGAAAAGAGACTGGTGAGGGTTAA
GGTTGAAGCAGTAACAAATGGTGAAGGTGATGCTGATCATCACAAACAGAAACACAAGAGTGAACTA
GGGTCCCCTCCTCCACCTCCGGATTCATGGTCCTGGCGGAAATATGGTCAAAAACCCATCAAAGGATCT
CCTTATCCCAGGGGTTACTACAGGTGCAGCACAGGCAAGGGATGTTTGGCCAAGAAACAAGTAGAAG
GGTGCAAAGAAGATGCTTCCGTCTTCATCATCACTTACACCTCTCCCCACAACCACAACCGTATTGGTC
ATGCAGGGCCAATGGCCAACTCGAGCACCCAAAAGCATCGACCTCATTCTCCTGACAAACGAACCCAC
AGCAGCAATAATAAGCACACATGTGTTGAGCATCAGCACAGCAGCTCAAATGCAATGCCAATGGCAAA
ACAGGAGGCACAAGAGCCAGAGCCTTATGTGGTGAGCCTCGATGGTATTACCCCTCTGCCTCTCCCTCT
ATCTCATCATGATCAACAGCACCCCTTTGCTTCATATGATCTCAAGAGTCATCCCGCATCACAAGGAAAC
TAGAGGATCAAAACTACGACTTCTTTGATGAGCTTGAGTTGGACCTTTAA

>*HuWRKY67*

ATGATCGGAGGTACCAAAATGCAACAAGTGACAATCAGTACTGTTGGACTAACACATCAAGATGGCTAT
TCATGGAGAAAATATGGTCAAAAGCCAATTGTCAATGCAAAGCATCCAAGGTATATAAGTGTAAGGACG
ATGCACATTGACTAA

>*HmoWRKY67*

ATGATCGGAGGTACCAAAATGCAACAAGTGACAATCAGTACTGTTGGACTAACACATCAAGATGGCTAT
TCATGGAGAAAATATGGTCAAAAGCCAATTGTCAATGCAAAGCATCCAAGGTATATAAGTGTAAGGACG
ATGCACATTGACTAA

>*HuWRKY68*

ATGGCCGAGAAGTACAACAAAACTCAGCCTGAATCAAATTCAAATCCAACTTTGATGGAGATGAACAA
CAACATCAGCGATTACCAGACGATGTTCGACGCTCCGTTTCAGTTGGGTATATTCGACAACTACACCAA
GCCCGGCTACTCATCCGGGTTCGGGTTCATGGATATGCTCGGAATCCAAGATTTCGCCGCCCCTACTTCC
TTGTTCGATCTCCTTCCGCCGCCGCCGGAGCCTCCGTTGCAGCCGCCTCGTCCGGATCCGTCTCCCAGT
AGCTCGGGTCACCCGGGAGATAATCAGTCCTCCGAAGCGGAGGTCGTCAACACCCCAGCCAACTCCAT
CTCCGTCTCTTCCTCCTCTTCGGAAGCCGCCGATGATAAGGCGGCCACACGCGCCGCCGCTAGTGGCG
GAGGTGATGGAGATGGAGATGGTGAAGAGGAGGTGGAGCACCCGAATCTTCCAGGAGAAGATAACAA
GTCTAAGAAGCAGTTAAAGGTGGCAAAAAAGAAAAATCAGAAAAGGCAGAAAGAGCCGAGATTTGC
GTTCATGACTAAGAGCGAAGTTGATCACCTTGATGATGGCTACCGTTGGCGTAAGTATGGCCAAAAAGC
CGTTAAGAACAGCCCCTTTCCCAGGAGCTACTACCGTTGCACCACAGCAGCATGCGGGGTGAAGAAG

AGGGTGGAACGGTCCTCAGACGACCCAACCATCGTGGTGACCACCTACGAGGGGCAGCACACCCATC

CGTGCCCGGTGGTCACCCGTGGTGGCATAGGCCTCAACCTAGGCCTAGGGACAGGACTTGTCCCCTCA

CCATCACCACTGTCGCCTTTTCTGGATCAATGCAGCACCACTCCTACTATAGGAGGCAGTGCTGCATCAT

CCGCTCTCTTCCTCCACCACCACCATCACACATTCCCTGGTTATTTCATAACCCCAACCCACTCTCCCTT

CTCCTTAACTGGCGACGGTGGTGGTAGTGGTGGTGCTGTGCAGCCTGCTCAAAATCAAAATCAAGGGA

ACTTGATAATACAAAACATTAGCCTCCAGCAGAGACAAATGATATTGAGGGCTCCATGTTTGGCTTCTTC

TTTGCACCAGCAACAAGTTAGAGATGATGGGCTTCTTCAGGACATCGTGCCATCGCAAATGCGCAACC

AAGCCACAGCAAAAGAAGAGTAA

>*HmoWRKY68*

ATGGCCGAGAAGTACAACAAAACTCAGCCTGAATCAAATTCAAATCCAACTTTGATGGAGATGAACAA

CAACATCAGCGATTACCAGACGATGTTCGACGCTCCGTTTCAGTTGGGTATATTGGACAACTACACCAA

GCCCGGCTACTCATCCGGGTTCGGGTTCATGGATATGCTCGGAATCCAAGATTTCGCCGCCCCTACTTCC

TTGTTCGATCTCCTTCCGCCGCCGCCGGAGCCTCCGTTGCAGCCGCCTCGCCCGGATCCGTCTCCCAGT

AGCTCGGGTCACCCGGGAGATAATCAGTCCTCCGAAGCGGAGGTCGTCAACACCCCAGCCAACTCCAT

CTCCGTCTCTTCCTCCTCTTCAGAAGCCGCCGATGATAAGGCGGCCACACGCGCCGCCGCTAGTGGCG

GAGGTGATGGAGATGGAGATGGTGAAGAGGAGGTGGAGCACCCGAATCTTCCAGGAGAAGATAACAA

GTCTAAGAAGCAGTTAAAGGTGGCAAAAAAGAAAAATCAGAAAAGGCAGAAAGAGCCGAGATTTGC

GTTCATGACTAAGAGCGAAGTTGATCACCTTGATGATGGCTACCGTTGGCGTAAGTATGGCCAAAAAGC

CGTTAAGAACAGCCCCTTTCCCAGGAGCTACTACCGTTGCACCACAGCAGCATGCGGGGTGAAGAAG

AGGGTGGAACGGTCCTCAGATGACCCAACCATCGTGGTGACCACCTACGAGGGGCAGCACACCCATC

CGTGCCCGGTGGTCACCCGTGGTGGCATAGGCCTCAACCTAGGTCTAGGGACCGGACTTGTCCCCTCA

CCATCACCACTGCCGCCTTTTCTGGATCAATGCAGCACCGCTCCTACTATAGGAGGCAGTGCTGCATCA

TCCGCTCTCTTCCTCCACCACCACCATCACACATTCCCTGGTTATTTCATAACCCCAACCCACTCTCCCT

TCTCCTTAACTGGCGACGGTGGTGGTAGTGGTGGTGCTGTGCAGCCTGCTCAAAATCAAAATCAAGGG

AACTTGATAATACAAAACATTAGCCTCCAGCAGAGACAAATGATATTGAGGGCTCCATGTTTGGCTTCT

TCTTTGCACCAGCAACAAGTTAGAGATGATGGGCTTCTTCAGGACATCGTGCCATCGCAAATGCGCAA

CCAAGCCACAGCAAAAGAAGAGTAA

>*HuWRKY69*

ATGGTTATGGAAAATAAATATCGGAGTAGCTCCTATTTCACCGATCAAGACGACCCACAAAGCTCGTCT

GAGAATCGGCCCGACTCCCCCCTCTCCGGTGATGACTCCCGAGCTACCGCCACCGCCCCTTCCCCTAAG

AGGAGGAGAAGCATCCAGAAGAGAGTGGTGCATGTACCAATAGGAGATGGTGACGGATCAAGGACCA
AAGGGGAGGCGTATCCACCGTCGGATTCATGGGCGTGGAGAAAGTACGGTCAGAAACCAATCAAGGG
CTCTCCATATCCCAGGGGATACTACCGATGTAGCAGCTCCAAAGGGTGCCCTGCCAGAAAACAAGTGG
AGAGGAACCGTGTGGACCCCACCGTGCTTATTATTACCTACGCTTGTGAGCACAACCACCCCCTGCCTA
CCTCCAAGCACCACCATAGTCACCAGGTTGCCACCGCAACCACCACCCCAACTGAAAACGCCACCGCT
TCTCCTGCCACGACCACCACGTCGGCTGACGCTGATCAGGCCAATGACGGCGGTGACGAGCTGTTGGT
TGAGATGAATCCAAACCCGTTTGGTGACCCGCCCCAACTCGAGTTCGTCCGGGACGCCGTGATGTTGG
GCGTTGAATACAGTTGGATATCCGACATGATGTCCTTCAGTACGACATCGTTGCTTGTGGGGCCGGCGT
CCGCGTGTGATGACGTGGAGATGTCTACTAATTTTGCAATGAGAGAAGAAGACGAGTCATTGTTTGGA
GACCTTGATGAACTGCCGGAATGCTCTCTGGTTTTTCGGAGAAATCAAGGAAACATTGTTGAAAGGTG
TAATGCCACTGCTCCACTTTGTGGTAGCACGGGATGA

>*HmoWRKY69*

ATGGTTATGGAAAATAAATATCGGAGTAGCTCATATTTCACCGATCAAGACGACCCACAAAGCTCGTCG
GAGAATCGGCCCGACTCCCCCCTCTCCGGTGATGACTCCCGAGCTACCGCCACCGCCCCTTCCCCTAAG
AGGAGGAGAAGCATCCAGAAGAGAGTGGTGCATGTACCAATAGGAGATGGTGACGGATCAAGGACCA
AAGGGGAGGCGTATCCACCGTCGGATTCATGGGCGTGGAGAAAGTACGGTCAGAAACCAATCAAGGG
CTCTCCATATCCCAGGGGATACTACCGATGTAGCAGCTCCAAAGGGTGCCCTGCCAGAAAACAAGTGG
AGAGGAACCGTGTGGACCCCACCGTACTAATTATTACCTACGCTTGTGAGCACAACCACCCCCTGCCTA
CCTCCAAGCACCACCATAGTCACCAGGTTGCCACCGCAACCACCACCCCAACTGAAAACGCCACCGCT
TCTCCTGCCACGACCACCACGTCGGCTGACGCTGATCAGGCCAATGACGGCGGTGACGAGCTGTTGGT
TGAGATGAATCCAAACCCGTTTGGTGACCCGCCCCAACTCGAGTTCGTCCGGGACGCCGTGATGTTGG
GCGTTGAATACAGTTGGATATCCGACATGATGTCCTTCAGTACGACATCGTTGCTTGTGGGGCCGGCGT
CCGCGTGTGATGACGTGGAGATGTCTACTAATTTTGCAATGAGAGAAGAAGACGAGTCATTGTTTGGA
GACCTTGATGAACTGCCGGAATGCTCTCTGGTTTTTCGGAGAAATCAAGGAAACATTGTTGAAAGGTG
TAATGCCACTGCTCCACTTTGTGGTAGCACGGGATGA

>*HuWRKY70*

ATGGATCATCTAATTATAGCCCAAATCATTGAAAATTTAGAGAGAGGCAGAGACTCTGTTCATCACATCA
GAGACCTCCTGAACCAACGGCCAGGATTAACCACTGATCACGATGATCAGGGCTACTCCAAAGGCCTT
GATGATCATTCAATGGTGGCCCTGAATGCACTGGATTATGCCCTCTCTATGCTCCATAACCCTGATAATTT
CAGCTCCGAGGATAACCAATTTGGGAATTCTAACTCTCATGATTCGGCTGCTGAGACTCCTAGGAAAGA

TAGGAGAGGCTGCTACAAAAGAAGGAGGAATGTTGAATCAAGAGTAGTGGATACCCCAACTCTAACCG

ATGATGGGTTTGCTTGGAGAAAATATGGCCAGAAAGTGATCCTTAATACTACTCATCCAAGGAATTACTA

CAGATGCACACACAAGATAGATCAGGGGTGCCAAGCAACAAAACAGGTGCAGCAAATCTCAGAAAAT

CCCACAAAGCACAGGATAATTTACCATGGCAACCACACCTGCAAAAACCCTCTTAAAATCCCTTCTGTT

TTTATCAACTCCACTACCCCAGAAAACCCAGATTCCTCTGTTTATCTCAGCTTTGAGGCAAACACTCCC

ACATCCCAACAGGGCCTTAACCATCCAACCCCCTCAAATTTCTTCTTCCCCAACAATATGGTGAAGCAG

GAGTACGGACCCTTCAAGGCGGAGACGCCATCAGCACCGTCTGATCATCATCAGGACCATTCATCACCA

TCATCCCCTGATCGAGCCACCACTACCCCTGGTTCTGAGATCTCATCAGGAGAGGTGCACTCCCTGGGG

ACTGTGGCGAGTCCCCAGGGAATGGACATCGCGGGGGACTATTACTGCTACATGATGGCTGCTGAGCC

TGGTGGTTTGGACCAAATCTTGCAGAGTTTGATCTGTGCATGA

>*HmoWRKY70*

ATGGATCATCTAATTATAGCCCAAATCATTGAAAATTTAGAGAGAGGCAGAGACTCTGTTCATCACATCA

GAGACCTCCTGAACCAACGGCCAGGATTAACCACTGATCACGATGATCAGGGCTACTCCAAAGGCCTT

GATGATCATTCAATGGTGGCCCTGAATGCACTGGATTATGCCCTCTCTATGCTCCATAACCCTGATAATTT

CAGCTCCGAGGATAACCAATTTGGGAATTCTAACTCTCATGATTCGGCTGCTGAGACTCCTAGGAAAGA

TAGGAGAGGCTGCTACAAAAGAAGGAGGAATGTTGAATCAAGAGTAGTGGATACCCCAACTCTAACCG

ATGATGGGTTTGCTTGGAGAAAATATGGCCAGAAAGTGATCCTTAATACTACTCATCCAAGGAATTACTA

CAGATGCACACACAAGATAGATCAGGGGTGCCAAGCAACAAAACAGGTGCAGCAAATCTCAGAAAAT

CCCACAAAGCACAGGATAATTTACCATGGCAACCACACCTGCAAAAACCCTCTTAAAATCCCTTCTGTT

TTTATCAACTCCACTACCCCAGAAAACCCAGATTCCTCTGTTTATCTCAGCTTTGAGGCAAACACTCCC

ACATCCCAACAGGGCCTTAACCATCCAACCCCCTCAAATTTCTTCTTCCCCAACAATATGGTGAAGCAG

GAGTACGGACCCTTCAAGGCGGAGACGCCATCAGCACCGTCTGATCATCATCAGGACCATTCATCACCA

TCATCCCCTGATCGAGCCACCACTACCCCTGGTTCTGAGATCTCATCAGGAGAGGTGCACTCCCTGGGG

ACTGTGGCGAGTCCCCAGGGAATGGACATCGCGGGGGACTATTACTGCTACATGATGGCTGCTGAGCC

TGGTGGTTTGGACCAAATCTTGCAGAGTTTGATCTGTGCATGA

>*HuWRKY71*

ATGTCTAGTAATTCAAGGAAGGATGATATCCCATACAACCTCCTCGACTCATCACCACCACCATTAACCC

ATGAAGATCTCTTCTTCCCCTTTCCGAACAACATCTACACTAACCCTACTGCGCAAGCCCTCCAAGAATT

AGGGGGAGGTTTTGATGACCATAATAGTAACACCAATAATTACATGACCTCCTTCAATGAGTACTTACAT

GGATCTATGGACTTCAACACCCTCACCAGGAGTCTAGGGTTGTCTTCCTCCGTCGTCGTCAATGGCGGT

GAGAAGCTGGTGGAGATGGAGGTGCAGGCTGCCACCGGAAGCGGGGAGAACCCCGGCACCCCGAAC
TCGTCGATCTCCTCCTCCTCCACCGAGGCTGCCGAGGATTTGGGTGGGAAGAGCGTGGAGAAGGGTGG
TAAGGTACAATCTAAAGAAGCATCTATTGAGCAAGGTGGAGAAGAGTCAAAGAAAGGGAACAAGGGA
AAGAACAAGAAGGGAGAGAAGAAGCAAAGGGAACCAAGATTTGCATTCATGACAAAGAGTGAGATA
GATCATCTCGAGGATGGGTATCGATGGAGAAAATATGGGCAAAAGGCCGTCAAGAACAGTCCTTATCC
GAGGAGCTACTATCGCTGCACAACACAAAAGTGCACGGTGAAGAAGAGAGTAGAGCGATCGTTCCAA
GACCCCTCCATCGTGATCACAACCTACGAGGGCCAACACAACCACCCAGTTCCGGCCACACTACGTGG
CCATGCAGCCTTTCTGCCGCATTACTCCATGCTAACAACACCAACACCAGCATTAGCACCATCTTCGAG
CTTTCCTCAGGAGTTACTAGCTCAACTAGGGCATGGTCGAGGAGGTATTAACACCGGTGCTTCTTTCTC
TTTTCAACAGCTCCCCCACTTCCAACAACATCAGCTTCATCATCGTCGTGCTCAACAACTTATGCCAAA
TGATGACTACGGGCTTTTGCAAGATGTTGTGCCTTCCTTCCTTCTGAAGCAAGAGCACCCTTAG

>*HmoWRKY71*

ATGTCTAGTAATTCAAGGAAGGATGATATCCCATACAACCTCCTCGACTCATCACCACCACCATTAACCC
ATGAAGATCTCTTCTTCCCCTTTCCGAACAACATCTACACTAACCCTACTGCGCAAGCCCTCCAAGAATT
AGGGGGAGGTTTTGATGACCATAATAGTAACACCAATAACTACATGACCTCCTTCAATGAGTACTTACAT
GGATCTATGGACTTCAACGCCCTCACCAGGAGTCTAGGGTTGTCTTCCTCCGTCGTCGTCAATGGCGGT
GAGAAGGTGGTGGAGATGGAGGTGCAGGCTGCCACCGGAAGCGGGGAGAACCCCGGCACCCCGAAC
TCGTCGATCTCCTCCTCCTCCACCGAGGCTGCCGAGGATTTGGGTGGGAAGAGCGTGGAGAAGGGTGG
TAAGGTACAATCTAAAGAAGCATCTATTGAGCAAGGTGGAGAAGAGTCAAAGAAAGGGAACAAGGGA
AAGAACAAGAAGGGAGAGAAGAAGCAAAGGGAACCAAGATTTGCATTCATGACAAAGAGTGAGATA
GATCATCTCGAGGATGGGTATCGATGGAGAAAATATGGGCAAAAGGCCGTCAAGAACAGTCCTTATCC
GAGGAGCTACTATCGCTGCACAACACAAAAGTGCACGGTGAAGAAGAGAGTAGAGC
GATCGTTCCAAGACCCCTCCATCGTGATCACAACCTACGAGGGCCAACACAACCACCCAGTTCCGGCC
ACACTACGTGGCCATGCAGCCTTTCTGCCGCATTACTCCATGCTAACAACACCAACACCAGCATTAGCA
CCATCTTCGAGCTTTCCTCAGGAGTTACTAGCTCAACTAGGGCATGGTCGAGGAGGTATTAACACCGGT
GCTTCTTTCTCTTTTCAACAGCTCCCCCACTTCCAACAACATCAGCTTCATCATCGTCGTGCTCAACAAC
TTATGCCAAATGATGACTACGGGCTTTTGCAAGATGTTGTGCCTTCCTTCCTTCTGAAGCAAGAGCACC
CTTAG

>*HuWRKY72*

ATGGACGCTAATCGAGAGACCCGTAGCGTCAACAATGGAGCCAAAGAAGAAAAAGATGTCGAAAGCG

TCAGCCATGATAATGACTACAAAGAAGAGACGAAGGTGAGATTCAAAGATGAGCATGATAAGAGCTCC
AAAACCTCTTCCCCAAAACATGAAAATCCAAGGATTTGCAAAGAGGAGGATGAACTTGAATCTGCAAA
AGAAGAAAATGAAAGGCTAAAAAAGATGCTGGAACAAGTTGAGAATGATTACCAATCCCTGCAAAGG
CGCTTCTTCAACATCTTTCAACATGAACCTCCCCTGCAATCAGCACAAAAGTCTGTGAGCAATGATACT
CAAAAGGAAGAAGAAGAACCCGAAGTTTCGCTTTGTCTTGGGTTACAGACTCCAACGAAACATGATA
ACACTGGCAGCTCCAGCAAAGACAAAGTGGATGCTGAGCTGATGGAGGAAGAGCTATCTCTTGGACTA
GACTTGAAATCCTCACCTAAAGTTGATTTAACAGAGACAGGATCGGAAAAGAGTGATGGCATTGAAGA
TATGAGAGAAGATGAACGAGGAGAGACTTGGCCTCCCAGTAAAGTTTTGAACACAATGAAAAAAAGC
AGAAAGGGCGAAGAGCTTCCAGAACCAAACAATGCAAAAAGAGCAAGAGTTTGTGTACGAGCGAGG
TGCGATACACCAACAATGAATGATGGTTGCCAATGGAGAAAATATGGGCAGAAAACTGCCAAAGGAAA
TCCTTGTCCTCGTGCATACTATCGCTGCACGGTAGCACCATCATGTCCAGTCAGGAAACAGGTGCAGAG
ATGTTCAGAGGACATGTCTATTTTAATAACCACCTATGAAGGGACACACAACCACCCACTTACTGTAGC
AGCCACAGCCATGGCTTCCACAACCTCAGCTGCAGCATCCATGCTCTTATCTGGCTCATCAACCTCAAA
AGTCCTTAGTTCCTCTGCTCCCATAACTTCAACTACTAATCTTAATGGATTGAACTTCACTGTCTACAAC
AATGCAAGAACAGGGCAATTCAACTTACCTAGTTCTTCTTCACCAGCACCACCAACAATCACCCTGGAT
CTCACTGGCCCTCATTCCTCGCATTTCAGCAGGCAATTCTCAAATATTTTTCCATCCAAGTACTCGTCCA
GAAGCCTCGATTTCTCTTCTTCAGAAGCAAACACGTTTGCAACTGCTTGGAACAATCACAGTACTTACA
TAAATTACGGTCCATTGCCCGCTACTAAAACTCCTACTGGATCCCTTAGTTTCGGAAAACCAACCCTAC
AACAAATAAGCTACCCTTACATGGAAAGGACCAATCAAGTTGCCACTTCTTCGTCTTCTTCGTTTGCTT
CTCAACAGCTCTTGACAGAGACACTGACCAAGGTACTTAGTTCAGACCCAAGTTTCCAGTCAGCCGTA
GCAGCTGCAATTTCATCAATGGTGGGTGGAAATAATACCCTCAATGCAATGAATCAGGTCAATGCGAAT
AGTCTGGGTCCAAACTTGAAATGGGGGGATCCAGTTCAGGGGATTTCAATCAACTCATTGCCTGAGAA
TGGCAACCCATATACACCAAATTACTCAAACAAGCTGCCATTACCATCAACTTCCAATTCTCCAGCCGT
GGGAAGCTTCATGCTTCTTCAACCTCAATTTCCATTTTCTCTATCTAAGAGCCCTTCTACCTCAGATCTTA
GAGAGTAG

>*HuWRKY74*

ATGGAGATTGAAATAGCCAACAAAGTGGCTGTGGAGAGTAGCCACAGGGTTCTCTCTCTCATTTGCCA
AAAACAAGGCTCTCAGTCCCAAAACCTAATGGCACAAACTGGGGATGCTGTTTGCAAGTTCAAGAGA
GTAGTCGATCTCCTAGAGAACAGGTGTGGTCATGCCAGGATGAGAAGATTGAGGAAGTTACCCCCACC

TTTGCCCCAAAAAACTCTGCTACAAACCCCACATTCTAACCCAGAATCTTCACCCAAACCTCTGCAATT
ACTCCCATCAGGTTTTCTTAGGCACCCAGTTTCAGAAATTGATCAAAAATTTCAGAATCCGGCCAACCA
AAGTGCCCACAAGATGTTTGGTGAAAGACCAGGCATTGATATTAGTCTATCAGTTAAACCTCCTGGCCA
GATTCCTACCCAACAAAAGCCTTCTTATGACATCCAATTCTTCCAGCAGCAACAGATGCACCAGCAGAT
GCTGCAGAGGTTGCAATTTCAGCAGCAAATGAAGTTTCACCAGGCTGAGACGATGAACTCCTCCAGGA
GTGGAAGTGCCCTTAATTTGAAGTTTGATGGGTCAAGTTGTACTCAAACCATGTCATCCAATAGGTCTT
TTATGTCATCTTTAAGCATAGATGGTAGCATAGCGAATCCAAGTGGGAATTCATTCCAATTGATTGGTCT
GCCTCAGTCATCTGACCCCAATTCGCAGCATCTTAGGAGGAAGTGCCTTGTCAAGGGTGAGGATGGGA
GTGTCAAATGTGGGAGCAGTGCCAAATGCCATTGCTCAAAGAAGAGGAAACTGAGGATTAAGAGGTC
GATCAAGGTGCCAGCGATAAGTAACAAGGTTGCAGACATCCCCCCAGATGAATACTCATGGAGAAAGT
ACGGGCAGAAGCCAATTAAGGGGTCACCTCATCCCAGGGGATACTACAAGTGCAGTAGCATGAGAGGC
TGCCCAGCAAGGAAGCATGTGGAGAGGTGCCTCGAGGATCCTTCGATGCTGATTGTCACATACGAGGG
TGAGCACAATCATGCCAAGTTAGTAGCATCTCATTCCGCTCACACCTGA

>HmoWRKY74

ATGGAGATTGAAATAGCCAACAAAGTGGCTGTGGAGAGTAGCCACAGGGTTCTCTCTCTCATTTGCCA
AAAACAAGGCTCTCAGTCCCAAAACCTAATGGCACAAACTGGGGATGCTGTTTGCAAGTTCAAGAG
AGTAGTCGATCTCCTAGAGAACAGGTGTGGTCATGCCAGGATGAGAAGATTGAGGAAGTTACCCCCAC
CTTTGCCCCAAAAAACTCTGCTACAAACCCCACATTCTAACCCAGAATCTTCACCCAAACCTCTGCAAT
TACTCCCATCAGGTTTTCTTAGGCACCCAGTTTCAGAAATTGATCAAAAATTTCAGAATCCGGCCAACC
AAAGTGCCCACAAGATGTTTGGTGAAAGACCAGGCATTGATATTAGTCTATCAGTTAAACCTCCTGGCC
AGATTCCTACCCAACAAAAGCCTTCTTATGACATCCAATTCTTCCAGCAGCAACAGATGCACCAGCAGA
TGCTGCAGAGGTTGCAATTTCAGCAGCAAATGAAGTTTCACCAGGCTGAGACGATGAACTCCTCCAGG
AGTGGAAGTGCCCTTAATTTGAAGTTTGATGGGTCAAGTTGTACTCAAACCATGTCATCCAATAGGTCT
TTTATGTCATCTTTAAGCATAGATGGTAGCATAGCGAATCCAAGTGGGAATTCATTCCAATTGATTGGTCT
GCCTCAGTCATCTGACCCCAATTCGCAGCATCTTAGGAGGAAGTGCCTTGTCAAGGGTGAGGATGGGA
GTGTCAAATGTGGGAGCAGTGCCAAATGCCATTGCTCAAAGAAGAGGAAACTGAGGATTAAGAGGTC
GATCAAGGTGCCAGCGATAAGTAACAAGGTTGCAGACATCCCCCCAGATGAATACTCATGGAGAAAGT
ACGGGCAGAAGCCAATTAAGGGGTCACCTCATCCCAGGGGATACTACAAGTGCAGTAGCATGAGAGGC
TGCCCAGCAAGGAAGCATGTGGAGAGGTGCCTCGAGGATCCTTCGATGCTGATTGTCACATACGAGGG
TGAGCACAATCATGCCAAGTTAGTAGCATCTCATTCCGCTCACACCTGA

>*HuWRKY75*

ATGGAGAATAATCCCATGGCTCCCCCCTACTTATCACCACCATTAAGTAATTTCCCTTTCTCAACAAACT
TGTTATTTTCTCATGATAATAACACTAACAATTCCCCCATAATACCGTCTTTTCATCAACATGATCATCAC
CTTCTAGGGTTGAAGAGAGAGAATAGTAATGAGGCCGGATCGTCGAGTGGTGTAATCAAAGAAGAAGA
GAAGAAAGAGGGCAAAGAGAAGAAGGTCCGAAGGCATCGATACGCCTTTCATACGAGAAGTCAGGTT
GATATACTTGATGATGGATACCGATGGAGGAAGTACGGCCAGAAAGCTGTCAAGAACAACAAGTTCCC
TAGAAGCTATTATCGATGCACACATCCGGGTTGTAATGTGAAGAAGCAAGTGCAAAGATTAACCAAAGA
TGAAGAGGTAGTGGTGACAACCTATGAAGGGGTTCATACACACCCAGTTGAGAGATCCACTGATAATTT
TGAGCATATCTTGAAACAAATGCAGATTTATGTACCTCCTTGA

>HmoWRKY75

ATGGAGAATAATCCCATGGCTCCCCCCTACTTATCACCACCATTAAGTAATTTCCCTTTCTCAACAAACT
TGTTATTTTCTCATGATAATAACACTAACAATTCCCCCATAATACCGTCTTTTCATCAACATGATCATCAC
CTTCTAGGGTTGAAGAGAGAGAATAGTAATGAGGCCGGATCGTCGAGTGGTGTAATCAAAGAAGAAGA
GAAGAAAGAGGGCAGAGAGAAGAAGGTCCGAAGGCATCGATACGCCTTTCATACGAGAAGTCAAGTT
GATATACTTGATGATGGATACCGATGGAGGAAGTACGGCCAGAAAGCTGTCAAGAACAACAAGTTCCC
TAGAAGCTATTATCGATGCACACATCCGGGTTGTAATGTGAAGAAGCAAGTGCAAAGATTAACCAAAGA
TGAAGAGGTAGTGGTGACAACCTATGAAGGGGTTCATACACACCCAGTTGAGAGATCCACTGATAATTT
TGAGCATATCTTGAAACAAATGCAGATTTATGTACCTCCTTGA
