# Supplementary material for: Genome-Wide Identification of WRKY Gene Family in Pitaya Reveals the Involvement of HmoWRKY42 in Betalain Biosynthesis
Source: Int J Mol Sci. 2022 Sep 12;23(18):10568. doi: 10.3390/ijms231810568 (PMC9502481; doi:10.3390/ijms231810568)
Supplement: Supplementary file 1 [file ijms-23-10568-s001.zip › Supplementary Text S2. Nucleotide sequences of HmoCDOPA5GT1 promoter.pdf]

Supplementary Text S2. Nucleotide sequences of *HmocDOPA5GT1* promoter. W-box (T/C)TGAC(T/C) is indicated in box. Specific primers for promoter cloning are highlighted in yellow. The downstream primer is the reverse complementary sequence. Translation start site (ATG) is shown in red.

>HmoCDOPA5GT1 promoter

TGACTT**GGTCAA**AACGCTAAACGCTATACAAAGGGGGTGTTTGGTAACAACAGCTGTTTGGGATAAAA
TAACTGTTTGGCAAAATTTTAGCTATTTTGACAA**AGTCAA**CAGCTATTTGGCTACAAAAAACTCTTTTT
GAAAAAGTTATTTCCATTAGCCTTTTGAGAATAACTTTTTGTAAAAATGATATAATTGTCCATAAGTTTGA
AAAAATTACACACATCTCATTAAAAATTAATTCATAAATAAGGACATAAATGAAAATTTTACCAACATAT
TTTTGTAAAAACAACTAATTTTTACCAAACACTTTTTATAAAAACAACTAATATAAACAGCTAATTCAAA
CAGCCAGCTAACAGCTAACACAAACAGCTAACAACTAACAGCTATTTATCAAATAGGGCCAAAATACT
GTTGCCGAACACGACCTTAATGATTTATTTAATAGAATGTGTAAACTTTTATAAATGCTCGCATAGTGCAA
TAATCACGATGTTAATTTGATAAGATAACACACTAAGTACGATCGTCAATGAAATATAAGTGATTCAAAT
AACGTTCATCTATGCGAATTTATCAACAATGTAATTTTTCTCATCAAATTATGGTTCGGATTATAAATTATT
AAGAACAATTTTGAAAAATTGATGTGTCATGAATTAAGGATGATAAATAATATGAAA**GGTCAA**TTTGAT
TCTATGGCACGTGAATAACAATTTTGTTAGCACCTCAAAATATATAAATTTGTAAAAACAGATAACTTGT
GATTAGTGTATCTCCACAGCTTATATCATACACCCGATAGTGTATACTTGTAGAAGTCACATAATTGAAAT
ACATATTAATAACCGCTCATATGGCAAATACACATTGAAAAAGTATATATATGTGACAATTGAAGTACATG
CATCCAGGAGTATGTAATAATTTGCTGCCTACTCCCTAGTAAAAATGAGCCCAAATCTTTTTTCATATGTT
TATGGCCTGTTCTTTTCATGGGTAAATTTCAAAATACTAACATCAATCTACTTCAACCATTTTCAATCCAT
TTCAACACACTTTCACTATTTTAACCAAGTTTAATTTATCACATAAAAAATTCAACATACTTTAACCCACT
TCAGCTCACATCAACCTACTTCAACCCACTTCATTATTTTGCCCACCTCGATAGCTCAATTTACCCACGA
AAAAAATAGACCCTTAGATTTGGTATTGACTCTCCTATTAGTAGAACCGAGCCCATTTTGGTTTTCCCTA
GTAAAAAAAAAAAAAAATGGCTGGGAAATTCCAAAAATGGGGCTTTCCCTTGTTTTTTTGTTTTTAACA
CTCATTAGCACGGCAATAGTTAGAGTTTAGATTATTTGTAAATCATATTTATTTTTCTTTTCAATTTTTAAG
AGTCATTCTTTTTTTTACCTTAAGAAAAGGAGTTTTAAAGTGATGAGGAATTAAGTTGTAAGAAGTTGTA
TTTGCAATTATAATATATAGTTACATTTTAAAAAAGACGCGTGCTCAAAACATTTGCATTGATCAATTAAA
CACAAGCTTACGTTTCTCAATTAGAATTTATATAAGCATGCAAATCATCCAATACCATGACCATTGTGTAA
TTGGACGTACTCTTATGAGAAGAAGCGCCTGACTTGAATTGTACTTCATTTACCATTATAAAAAAAAATA
AAATGGCCACTATGGTAAATGGTGCATCATCAATACAAACTCTTACTTCAAATCACTTGCCAATGACAAT
GAGTTTGTTCACGGACAACTATTTGGTTGGGATAAGTATGAAGCATTATGGCTACTTCCTAAATTCTACT

ATGGCAACTTCTTACAAAATCACCGCCCAAACGCAACAATAATTAATATGGCTTACAAAATCAAATTGA

CAAATTCTATGGTAACTGTGGATTATTTGACGACACAAATTAGACCTAAAAATACAACCGCTTTTTCACA

GAGAAGAGAAGAAAGAGAAAGTGAGAGAAAGAGAGA**ATG**
